# L-3,4-Dihydroxyphenylalanine Recovers Circadian Rhythm Disturbances in the Rat Models of Parkinson's Disease by Regulating the D1R-ERK1/2-mTOR Pathway

**DOI:** 10.3389/fnagi.2021.719885

**Published:** 2021-08-19

**Authors:** Shuyuan Yang, Ying Wan, Na Wu, Lu Song, Zhihua Liu, Jiahao Zhao, Ying Liu, Zhenguo Liu, Jing Gan

**Affiliations:** Department of Neurology, Xinhua Hospital Affiliated Shanghai JiaoTong University, School of Medicine, Shanghai, China

**Keywords:** Parkinson's disease, Circadian rhythm, L-dopa, 6-OHDA, BMAL1, mTOR

## Abstract

**Objective:** Patients with Parkinson's disease (PD) frequently experience disruptions in the 24-h daily profile of both behavioral and biological markers. However, whether L-3,4-dihydroxyphenylalanine (L-dopa) influences these markers associated with circadian rhythm or not is still an open question. This study aims to explore the L-dopa effects on the rhythmic expression of core clock proteins [brain and muscle Arnt-like protein-1 (BMAL1) and circadian locomotor cycle kaput (CLOCK)], in the striatum of the rat model of PD and its underlying molecular mechanisms.

**Methods:** Unilateral 6-hydroxydopamine (6-OHDA)-lesioned rat models were used in this study. L-dopa administrations were adopted to investigate the changes of circadian rhythm in PD. The behavioral tests and the measurements of the blood pressure (BP) and temperature were evaluated. The striatum was collected at intervals of 4 h. Western blot was used to examine the expressions of clock protein and the molecular protein of the D1R-ERK1/2-mTOR pathway. The rhythmic expressions of symptom parameters and circadian proteins were analyzed using the Cosinor model and/or the coefficient of variability (CV) that was used to describe the variability of the 24-h rhythm.

**Results:** The circadian rhythms of BP and temperature were disrupted in 6-OHDA-lesioned PD rats compared with the sham group, while this process was reversed mildly by L-dopa treatment. The expressions of BMAL1 and CLOCK protein were rhythmic fluctuated without significant phase alterations when 6-OHDA or L-dopa was applied. Furthermore, the expressions of striatal BMAL1 protein in the 6-OHDA-lesioned group were significantly lower than those in the sham group at 04:00, 08:00, and 12:00, and the CLOCK protein was decreased at 04:00, 08:00, 12:00, 16:00, and 20:00 (all *p* < 0.05). The CV of the expressions of both BMAL1 and CLOCK was decreased in the 6-OHDA group; this process was reversed by L-dopa. Moreover, the CV of BMAL1 and CLOCK was elevated in the L-dopa rats. The phosphorylation levels of ERK1/2, S6K1, and 4E-BP1 in 6-OHDA-lesioned striatum were increased by L-dopa or D1 receptor agonist SKF38393 (*p* < 0.05, respectively), not by the combination of L-dopa and D1 receptor antagonist SCH23390, which was similar to the expressions of BMAL1 and CLOCK.

**Conclusion:** L-dopa recovers the circadian rhythm disturbances in PD rats by regulating the D1R-ERK1/2-mTOR pathway.

## Introduction

Parkinson's disease (PD) is one of the most common neurodegenerative disorders characterized by the progressive death of dopaminergic neuron projection from the substantia nigra to the striatum and the formation of Lewy bodies (Poewe et al., 2017). Patients with PD frequently experience disruptions in the 24-h daily profile of behavioral and biological markers, also known as circadian rhythm disturbances (CRDs). The CRD includes diurnal rest/activity rhythm, sleep–wake cycles, hormonal and body temperature rhythms, and dysregulation of the pattern of blood pressure (BP) (Li S. et al., 2017; Leng et al., 2019). The expression of circadian clock molecules is altered in different brain regions (Leng et al., 2019).

The circadian rhythm, which is an endogenously generated rhythm that roughly repeats every 24 h, is modulated by external cues (Hood and Amir, 2017) and regulated by clock genes/proteins expressed in all brain tissues (Kondratov et al., 2006). At the molecular level, the characteristics of mammalian circadian rhythm are based on the changes in the expressions of certain clock genes and their proteins, thus forming a transcriptional–translational feedback loop (TTFL) to keep a period of 24 h. The cell molecular clock of mammals is modulated by two interlocking TTFL. The core TTFL consists of two activators (i.e., CLOCK and BMAL1) and two repressors (i.e., cryptochrome and period), as well as other kinases and phosphatases. First, CLOCK and BMAL1 form the heterodimeric basic helix-loop-helix-PER/ARNT/SIM (PAS) transcription factor CLOCK:BMAL1 in the nucleus that activates the transcription factor transcription of the repressor Period (*Per*) and cryptochrome (*Cry*) genes (Huang et al., 2012). Then, PER and CRY proteins translocate and heterodimerize to form a complex in the cytoplasm, which then again translocate to the nucleus to interact with CLOCK:BMAL1, thus further inhibiting transcriptional activation. When the PER and CRY degrade, the repression on CLOCK:BMAL1 is relieved, and the cycle begins again with ~24-h periodicity (Busino et al., 2007). The alteration of expression patterns of clock molecules has been observed in PD (Wang et al., 2018).

L-3,4-Dihydroxyphenylalanine (L-dopa) is still the most effective treatment for PD. The parkinsonian typical motor symptoms can be significantly ameliorated by L-dopa. However, whether L-dopa influences the circadian rhythm remains unclear. Some studies showed that L-dopa treatment might aggravate the circadian dysfunctions of patients with PD, and some symptoms of CRD were regarded as a side effect of L-dopa therapy (LeWitt and Fahn, 2016). A recent study showed that altered plasma cortisol and melatonin secretion levels and the expression profiles of some clock genes in the specific brain region of 6-hydroxydopamine (6-OHDA) models were more severe after the long-term L-dopa administration (Li S. Y. et al., 2017). However, different conclusions were reported by other researchers. For example, Bellante et al. suggested that L-dopa treatment has a positive effect on CRD (Boulamery et al., 2010; Bellante et al., 2016). Boulamery et al. found that L-dopa treatment improves or recovers the circadian rhythmicity of temperature and heart rate (HR) (Boulamery et al., 2010). In addition, there were some hypotheses that some CRD was present in drug-naïve patients with PD, even before motor symptoms appear. This evidence implies that the circadian disturbance in patients with PD may be caused by neurodegeneration progression rather than L-dopa replacement therapy. Unfortunately, the link between L-dopa treatment and CRD in PD is still poorly understood.

Our previous study indicated that L-dopa therapy induces the activation of dopamine D1 receptor (D1R) that in turn mediates the mammalian target of rapamycin (mTOR) signaling pathway (Santini et al., 2009; Wu et al., 2018). Interestingly, the mTOR, which is an atypical Ser/Thr kinase, exhibits circadian rhythms in a variety of brain regions, such as the striatum. It also has a key role in regulating the synchrony of the central circadian clock in the suprachiasmatic nucleus (SCN) (Lipton et al., 2017). The activation or inhibition of mTOR could alter the circadian rhythmicity of peripheral clock models (Ramanathan et al., 2018). *In vitro* experiments demonstrated that if mTOR is constitutively activated, the expression of core clock proteins, including BMAL1 and CLOCK, is increased (Ramanathan et al., 2018). Therefore, it is believed that the hyperactivation of the D1R-ERK1/2-mTOR pathway induced by L-dopa might be involved in the alternations of BMAL1 and CLOCK protein.

In this study, we explored the effect of L-dopa treatment on the rhythmic expression of core clock proteins (i.e., BMAL1 and CLOCK) in the striatum of the rat models with PD through the regulation of the D1R-ERK1/2-mTOR pathway.

## Methods

### Animals

The adult, male, 6-week-old Sprague–Dawley (SD) rats, weighing 180–220 g, were used for the study. All of them were purchased from the Shanghai Laboratory Animal Corporation (SLAC, Shanghai, China). All the animals were housed in an environment with a temperature of 22 ± 1°C, relative humidity of 55 ± 5%, and a strict light/dark cycle of 12/12 h and had free access to food and water. All animal studies were analyzed in compliance with the regulations and guidelines of the Shanghai Jiao Tong University, School of Medicine Institutional Animal Care and conducted according to the guidelines of the National Institutes of Health (publication No. 80–23). All efforts were made to minimize the number of used animals and the distress they suffered.

### 6-Hydroxydopamine Models

Animals were acclimated for at least 1 week before the 6-OHDA injections. A 6-OHDA-lesioned rat model was established as previously described (Gan et al., 2014). In brief, pentobarbital [50 mg/kg body weight, intraperitoneal (i.p.)] was used to anesthetize all rats by i.p. injection. After being placed onto a stereotaxic frame (Narishige, Tokyo, Japan), 32 μg of 6-OHDA (Sigma–Aldrich Co., St Louis, MO, USA) (dissolved 8 μl of 0.9% saline containing 0.2% ascorbic acid, 4 μg/μl) was injected into two points of the right medial forebrain bundle of the rats, 4 μl per point, at a flow rate of 1 μl/min. Two coordinates were as follows: at anteroposterior (AP) −3.7 mm, mediolateral (ML) −1.7 mm, dorsoventral (DV) −7.8 mm; and at AP −4.4 mm, ML −1.2 mm, DV −7.8 mm. The tooth bar was set to −2.4 mm. The sham group rats underwent the same procedure with an injection of a saline solution into the targeted sites. Twenty-one days after unilateral 6-OHDA injection, the lesioned rats were tested by contralateral rotations. The rats exhibiting rotational behaviors (≥7 turns/min) following apomorphine injection (Sigma–Aldrich Co.) at a dose of 0.5 mg/kg were considered successful rat models of PD.

### Experimental Protocol and Drug Treatment

This experimental design was divided into two parts. In Part I, the influence of L-dopa on circadian rhythms at the molecular level was investigated in the PD model. The rats were divided into three groups: (1) 6-OHDA-lesioned group: PD rats received 0.9% saline i.p. administration once daily for 21 days; (2) 6-OHDA-lesioned+L-dopa group: PD rats were administrated with the L-dopa solution (25 mg/kg L-dopa and 6.25 mg/kg benserazide, i.p.) (Sigma–Aldrich Co.) once daily for 21 consecutive days; (3) sham group of saline-lesioned rats: PD rats were given saline once per day for 21 days instead of L-dopa and benserazide intraperitoneally.

**Figure 1:**
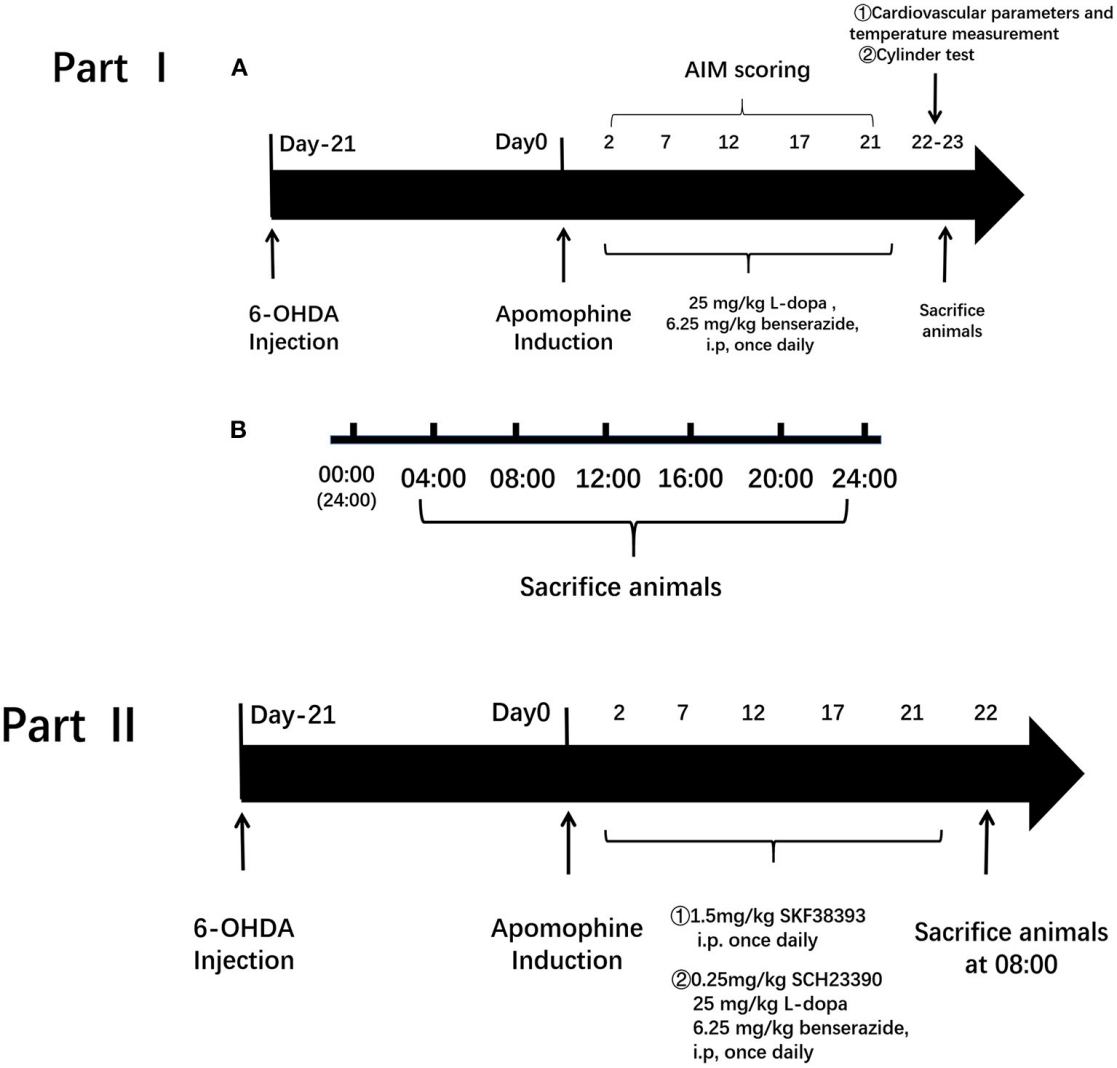
Experimental protocol. The experimental design was divided into two parts. Part I is to investigate the influence of L-3,4-dihydroxyphenylalanine (L-dopa) on circadian rhythms at the molecular level in the Parkinson's disease (PD) model. Group: (1) 6-hydroxydopamine (6-OHDA)-lesioned group; (2) 6-OHDA-lesioned+L-dopa group; (3) the sham group. Part II is to determine whether the D1R-ERK1/2-mTOR pathway was involved in the alterations of core clock protein induced by L-dopa. Group: (1) 6-OHDA-lesioned group; (2) 6-OHDA-lesioned+L-dopa group; (3) SKF38393 group; (4) SCH23390 group (SCH23390+L-dopa+benserazide); (5) the sham group.

In Part II, we determined whether the D1R-ERK1/2-mTOR pathway was involved in the alterations of core clock protein induced by L-dopa. The rats were divided into five groups: (1) 6-OHDA-lesioned group: the same as mentioned earlier; (2) 6-OHDA-lesioned+L-dopa group: the same as mentioned earlier; (3) SKF38393 group: PD rats were treated with the selected D1R agonist, SKF38393 (1.5 mg/kg, i.p.) once daily for 21 days; (4) SCH23390 group: PD rats were coadministered with L-dopa and benserazide and the selected D1R antagonist, SCH23390 (0.25 mg/kg, i.p.), once daily for 21 days; (5) the sham group was the same as mentioned earlier. The design and timeline of the experiments are shown in Figure 1.

## Behavioral Assessment

### Cylinder Test

The cylinder test was used to evaluate the functions of the forelimbs of the rats. On the 22nd day, the 6-OHDA-lesioned rats, the L-dopa group, and the sham group were put into a glass cylinder (diameter of 20 cm and height of 30 cm), and their body behaviors were observed for 5 min to count the number of wall contacts performed by the left and the right forelimbs. The rate of their left forelimbs was calculated as follows: the number of left forelimb contacts/the total number of left and right forelimb contacts.

### Abnormal Involuntary Movement (AIM) Assessment

The AIM was assessed in L-dopa-treated 6-OHDA-lesioned rats as previously described (Wu et al., 2018). In brief, the assessment of AIM consisted of four types: (1) axial AIM scores; (2) limb AIM scores; (3) prolingual AIM scores; (4) rotation AIM scores. The total scores of axial, limb, and orolingual movements were counted. Each type of the AIM score was rated from 0 to 4 according to the time the abnormal movements were present during the observation period. The AIM assessments were performed on day 2, 7, 12, 17, and 21 after L-dopa injection; scores were made every 20 min during the 120 min of the drug treatment.

### Measurement of BP and Temperature

The measurements of BP and temperature were performed on the 22nd day, every 2 h since 8:00. The non-invasive BP tail-cuff method was used to measure the cardiovascular parameters using a sphygmomanometer for rodents (BP2010A; Softron, Japan). The tails of rats were passed through the optical sensor and the compression cuff. Every rat was measured three times, and the average values were recorded. The body temperatures (°C) of rats were measured through the rectum by using a small thermometer, taking an average of three measurements.

### Striatal Protein Extraction

After the behavioral assessment, the rats in experimental Part I were sacrificed at the six time points (04:00, 08:00, 12:00, 16:00, 20:00, 24:00), and the lesioned striatal tissues were immediately removed. The rats in experimental Part II were sacrificed at 08:00. The method of extraction was previously described (Gan et al., 2015). The striatal tissues were homogenized in radio immunoprecipitation assay (RIPA) buffer containing 1 mM phenylmethanesulfonyl fluoride (PMSF). After being quantified to equal concentrations using the bicinchoninic acid (BCA) protein assay kit (Beyotime Biotechnology, China), the samples were boiled for 10 min in loading Buffer (Beyotime Biotechnology, China).

### Western Blot

The equal amount of protein samples was separated by sodium dodecyl sulfate-polyacrylamide gels (SDS–PAGE; Beyotime Biotechnology, China) and transferred onto polyvinylidene fluoride (PVDF) membranes (Millipore, Billerica, MA, USA) in a transfer buffer (EpiZyme, China) with 300 mA current. Then, membranes were blocked in 5% no-fat milk in tris-buffered saline+tween (TBST) (Beyotime Biotechnology, China) for 1 h at room temperature and incubated overnight at 4°C with primary antibodies against tyrosine hydroxylase (TH; 1:1,000, ab41528, Abcam, UK), BMAL1 (1:1,000, ab3350, Abcam), CLOCK (1:1,000, ab3517, Abcam), ERK1/2 (1:1,000, 4695S, Cell Signaling, USA), p-ERK (1:1,000, 4370S, Cell Signaling, USA), mTOR (1:1,000, 2983S, Cell Signaling), S6K1; 1:1,000, A16968, ABclonal, China), p-S6K1 (1:1,000, AP0502, ABclonal), 4E-BP1 (1:1,000, 9644T, Cell Signaling), p-4E-BP1 (1:1,000, AP0030, ABclonal), β-actin (1:1,000, AA128, Beyotime Biotechnology), or glyceraldehyde-3-phosphate dehydrogenase (GAPDH) (1:1,000, AF1186, Beyotime Biotechnology). Then, the membranes were subsequently washed with TBST (0.1% Tween 20, Beyotime Biotechnology, China) and incubated with horseradish peroxidase (HRP)-labeled secondary antibodies (1:2,000, Beyotime Biotechnology, China) at room temperature for 1 h. The protein bands were detected using an ECL assay kit (Millipore, Billerica, MA, USA) and measured by a Tanon 4,600 imaging system (China). The intensity of protein bands was analyzed by the ImageJ software (National Institutes of Health, NIH).

### Statistical Analysis

The data were expressed as mean ± SEM. The ANOVA was followed by the least significant difference (LSD) *post hoc* test for comparisons among various groups. The coefficient of variability (CV) was used to describe the variability of the 24-h rhythmic fluctuation of BP, the temperature, and the expressions of striatal BMAL1 and CLOCK protein. CV was expressed as a percentage and was calculated by the ratio of standard deviation (SD) to their average values (Mean) of these parameters at different time points in 24 h (CV = SD/Mean × 100%) (Schmidt et al., 2009). The rhythmic nature of the expression of BMAL1 and CLOCK protein was analyzed by the Cosinor model (Sundaram et al., 2020). The Cosinor analysis was performed with R version 4.0.5 (R Foundation for Statistical Computing, Vienna, Austria; http://www.R-project.org/) using the CircaCompare package. The statistical analyses were performed by using SPSS software version 17.0 (SPSS Inc., Chicago, IL, USA). The *p*-values < 0.05 were considered statistically significant.

## Results

### The Changes of the Behavioral Parameters and the Expression of TH in the Model Rats

**Figure 2:**
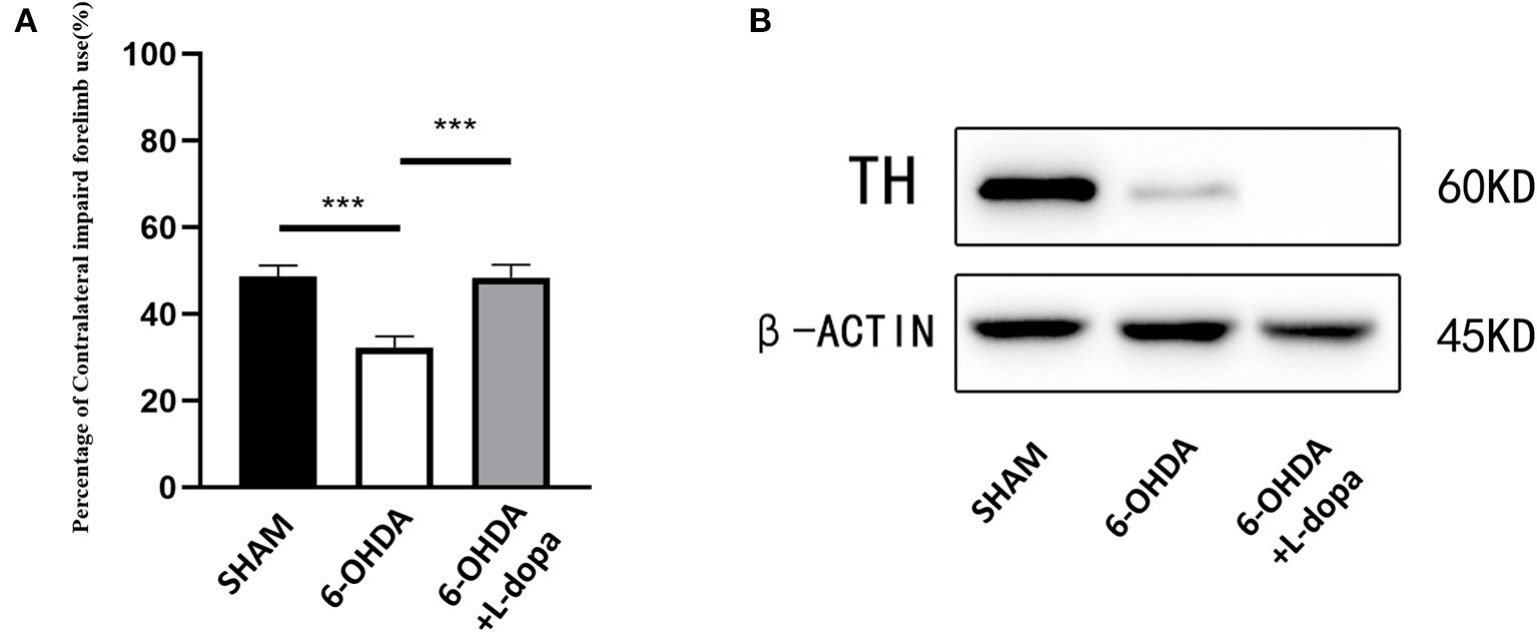
Cylinder test and examination of TH. Motor behavior assessments and the expressions of TH were compared among three groups. **(A)** The data of the cylinder test. The 6-OHDA injections reduced the percentage of left forelimb use, while after chronic L-dopa administration, the percentage of left forelimb use was improved (*p* < 0.001). *n* = 18–24 for each group. **(B)** Western blot was used to examine the expressions of TH in the different groups. *n* = 5 for each group. ***p < 0.001.

The cylinder test was performed to test the motor function of the 6-OHDA-lesioned group and the 6-OHDA-lesioned+L-dopa treatment group, compared with the sham group. As shown in Figure 2A, the 6-OHDA-lesioned rats showed decreased forelimb activity (32.28 ± 1.16%) compared with the sham group (48.65 ± 1.13%) (*p* < 0.001). The impaired forelimb activity was improved after L-dopa treatment (48.35 ± 1.36%) compared with 6-OHDA-lesioned rats (*p* < 0.001).

In addition, the rats of the 6-OHDA-lesioned+L-dopa group were further evaluated with the AIM scores. The total scores of the axial, limb, and orolingual (ALO) AIM increased during the period of L-dopa treatment from 5.54 ± 0.28 (day 2) to 11.21 ± 0.31 (day 21) (*F* = 34.27, *p* < 0.05). The axial AIM scores increased from 1.85 ± 0.11 to 3.93 ± 0.03 (*F* = 19.287, *p* < 0.05), the limb AIM scores increased from 1.56 ± 0.19 (day 2) to 3.69 ± 0.12 (day 21) (*F* = 17.633, *p* < 0.05), the orolingual AIM scores increased from 1.8 ± 0.20 (day 2) to 3.60 ± 0.20 (day 21) (*F* = 13.062, *p* < 0.05), and the rotation AIM scores increased from 1.67 ± 0.07 (day 2) to 3.81 ± 0.57 (day 21) (*F* = 26.53, *p* < 0.05) (Supplementary Figure 1).

Western blot showed the expression of TH protein dramatically decreased in the 6-OHDA-lesioned striatum (Figure 2B), which suggested that the model was successfully established and demonstrated that 6-OHDA caused striatal dopamine depletion and consequent motor dysfunctions. L-dopa administration improved the function of the impaired forelimbs.

**Figure 3:**
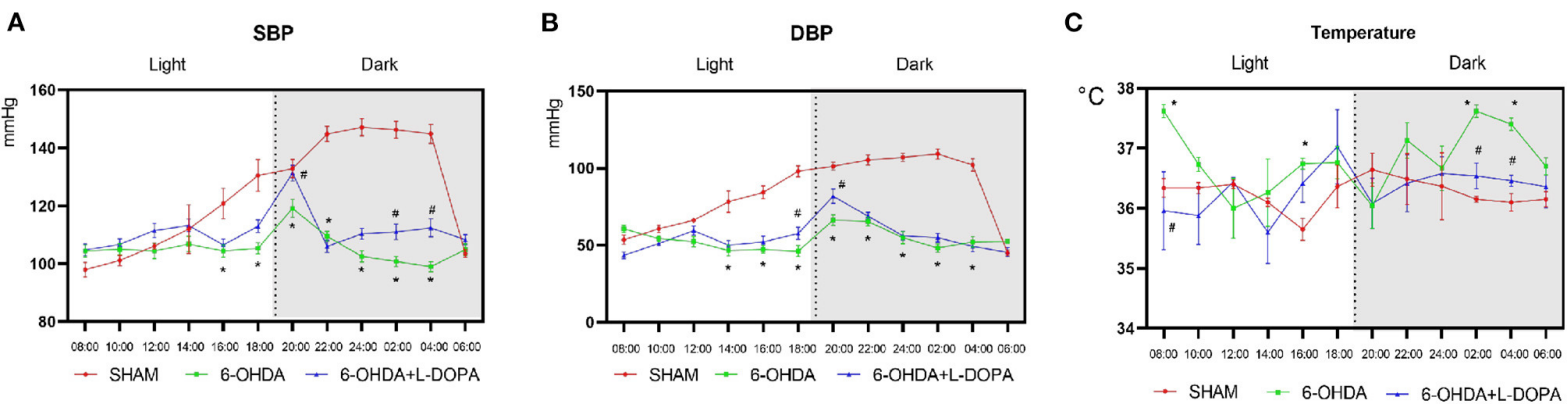
The measurement of BP and temperature. The BP and temperature were measured every 2 h since 8:00 on the 22nd day. The measurement of circadian rhythm-related parameters: **(A)** The curve of the systolic blood pressure (SBP); **(B)** The curve of the diastolic blood pressure (DBP); **(C)** The curve of the temperature. *p < 0.05, sham group vs. 6-OHDA-lesioned group; #*p* < 0.05, 6-OHDA-lesioned group vs. 6-OHDA+L-dopa group. *n* = 18–24 for each group.

Then, the parameters about circadian rhythm were monitored for 24 h, such as systolic BP (SBP), diastolic BP (DBP), and temperature. Compared with the sham group, 24-h mean SBP and DBP significantly decreased in the 6-OHDA-lesioned group and slightly increased after L-dopa treatment (Table 1). In the sham group, the BP was higher in the dark cycle than in the light cycle (Figure 3); SBP and DBP reached a peak between 24:00 and 02:00 and had the lowest level in the period from 06:00 to 08:00. The amplitude of SBP or DBP was almost 50 mmHg (Supplementary Table 1). In the 6-OHDA-lesioned group, the BP was significantly lower than that of the sham group, especially in the dark cycle; the peak of SBP and DBP was observed at 20:00, the difference between the highest level and the lowest level was smaller than that observed in the sham group (Figure 3; Supplementary Table 1). Similarly, the CV of SBP/DBP in this group was significantly decreased compared with the sham group, respectively (Table 2). After L-dopa treatment, the SBP increased at most time points in the dark cycle compared with the 6-OHDA-lesioned group (Supplementary Table 1), but the shapes of the curve of BP were similar. The CV of SBP/DBP in the 6-OHDA-lesioned+L-dopa group partially recovered to that in the sham group (Table 2).

**Table 1:** The average systolic blood pressure (SBP), diastolic blood pressure (DBP), and temperature over a 24-h period in the sham group, the 6-hydroxydopamine (6-OHDA)-lesioned group, and the 6-OHDA-lesioned+L-dopa group.

	**Sham** **(mean ± SEM)**	**6-OHDA** **(mean ± SEM)**	**6-OHDA+L-dopa** **(mean ± SEM)**	***F*-value**	***p*-value**
average SBP	124.00 ± 5.58	105.52 ± 1.46^*^	111.26 ± 2.01#Δ	7.192	0.003
average DBP	84.28 ± 6.63	53.90 ± 2.01^*^	55.22 ± 3.32#	14.979	0.000
average Temperature	36.26 ± 0.07	36.81 ± 0.16^*^	36.31 ± 0.11Δ	6.405	0.004

*The data were performed for group differences with ANOVA and then followed by the least significant difference (LSD) post hoc test. L-dopa, L-3,4-dihydroxyphenylalanine*.

* *: p < 0.05 by post hoc comparisons of sham group vs. 6-OHDA group*;

*#: p < 0.05 by post hoc comparisons of sham group vs. 6-OHDA+L-dopa group;* Δ: *p < 0.05 by post hoc comparisons of 6-OHDA group vs. 6-OHDA+L-dopa group*.

**Table 2:** The coefficient of variation (CV) of the SBP, DBP, and temperature in the sham group, the 6-OHDA-lesioned group, and the 6-OHDA-lesioned+L-dopa group.

	**Coefficient of variation**		
	**Sham (%)**	**6-OHDA (%)**	**6-OHDA+L-dopa (%)**
Systolic blood pressure (SBP)	15.60	4.79	6.26
Diastolic blood pressure (DBP)	27.26	12.94	18.98
Temperature	0.68	1.50	1.04

*L-dopa, L-3,4-dihydroxyphenylalanine*.

The temperature was significantly different among the three groups (Table 1). The temperature increased at 08:00, 16:00, 02:00, and 04:00 in a 6-OHDA-lesioned group compared with the sham group, while L-dopa significantly decreased the temperature at 08:00, 02:00, and 04:00 elevated by 6-OHDA (Figure 3C; Supplementary Table 1). These data indicated that 6-OHDA could induce abnormal BP and temperature rhythmicity, which could be reversed partially by L-dopa treatment.

### Changes of the Expressions of Striatal BMAL1 and CLOCK Protein and Their Rhythmicity in the Model Rats

**Figure 4:**
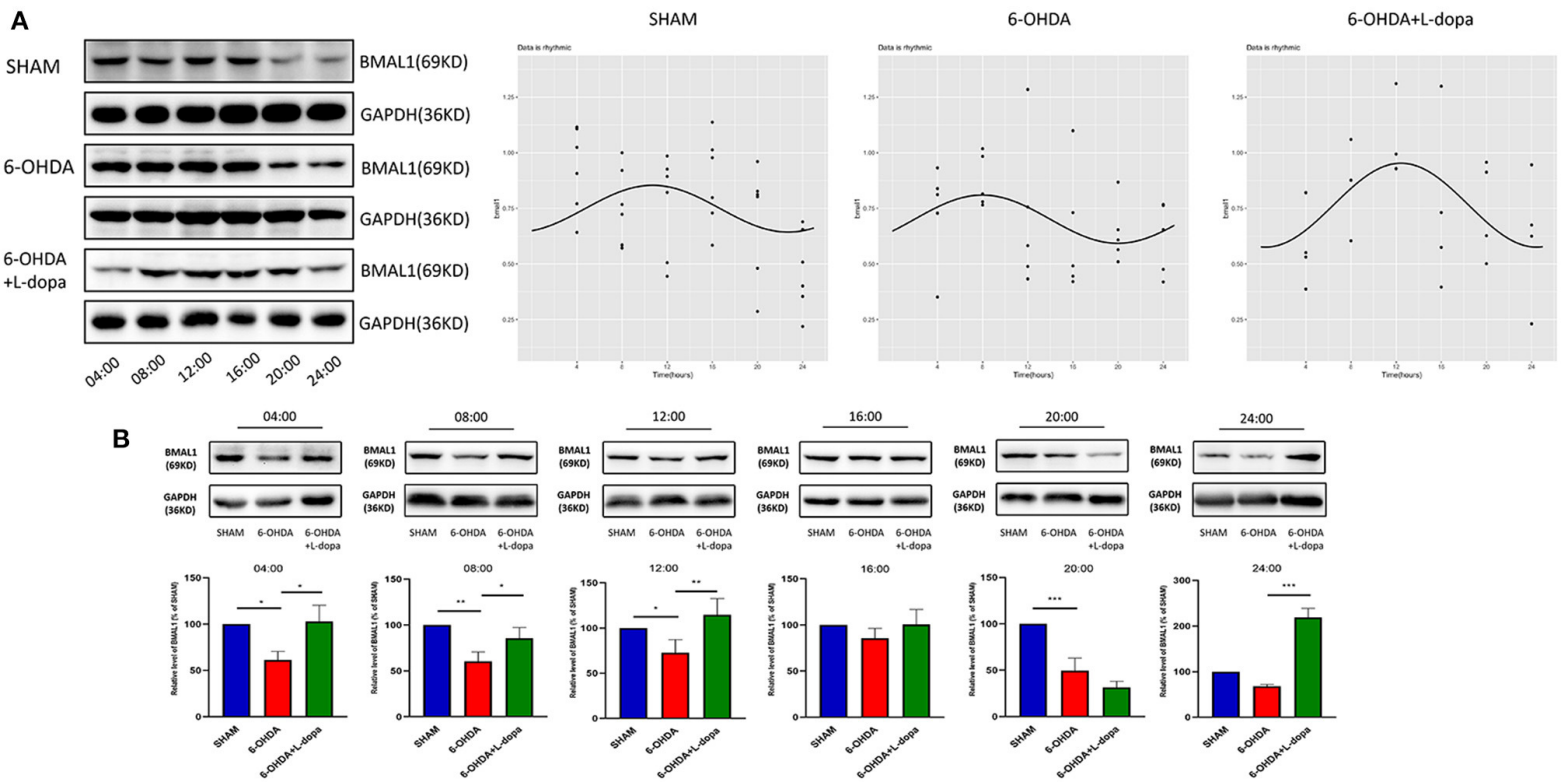
Western blot and the Cosinor analysis of BMAL1 protein. **(A)** Western blot and the Cosinor analysis of BMAL1 protein in sham, 6-OHDA-lesioned, and 6-OHDA-lesioned+L-dopa groups. The expressions of BMAL1 protein among the sham group, 6-OHDA-lesioned group, and 6-OHDA+L-dopa group at different time points (every 4 h since 04:00). All the fitted curves were rhythmic fluctuated. **(B)** The expression of BMAL1 protein at six time points during a day in sham, 6-OHDA-lesioned, and 6-OHDA+L-dopa groups. *p < 0.05. **p < 0.01, ***p < 0.001; *n* = 3–5 at each time point per group.

First, we explored the circadian rhythm alternations of BMAL1 protein in 24 h among different groups. The Cosinor analysis revealed the oscillations in the expressions of circadian BMAL1 protein in the striatum (Figure 4A; Supplementary Table 2). The acrophase in the sham group, the 6-OHDA group, and the 6-OHDA+L-dopa group were 2.80 ± 0.53, 2.09 ± 0.52, and 3.25 ± 0.38, respectively. The peak time of circadian rhythm corresponding to the acrophase was 10.7 (h) in the sham group, 8.0 (h) in the 6-OHDA-lesioned group, and 12.4 (h) after the L-dopa treatment (Table 3); however, without statistical significance (*p* > 0.05). Moreover, the circadian phases had no significant shift (*p* > 0.05, respectively), which indicates that 6-OHDA or L-dopa treatment did not alter the phase of the rhythm of striatal BMAL1 protein (Table 3). In addition, the CV of the 6-OHDA-lesioned group was 13.60%, which was smaller than that of the sham group (24.00%). After L-dopa administration, the CV was 22.10% (Table 4).

**Table 3:** The acrophase and the corresponding peak time of BMAL1 and CLOCK protein expression by using the Cosinor model.

**BMAL1**			***p*** **-value of acrophase**		
Group	Acrophase (estimates ± SE)	Peak time (h)	Sham vs. 6-OHDA	6-OHDA vs. 6-OHDA+L-dopa	Sham vs. 6-OHDA+L-dopa
Sham	2.8036 ± 0.5333	10.7	0.3442	0.0834	0.4947
6-OHDA	2.0881 ± 0.5207	8.0			
6-OHDA+L-dopa	3.2535 ± 0.3823	12.4			
**CLOCK**			***p*** **-value of acrophase**		
Group	Acrophase (estimates ± SE)	Peak time (h)	Sham vs. 6-OHDA	6-OHDA vs. 6-OHDA+L-dopa	Sham vs. 6-OHDA+L-dopa
Sham	2.4799 ± 0.2111	9.5	0.4949	0.1016	0.1344
6-OHDA	2.1530 ± 0.4446	8.2			
6-OHDA+L-dopa	3.5475 ± 0.7178	13.6			

*L-dopa, L-3,4-dihydroxyphenylalanine*.

**Table 4:** The CV of the relative level of BMAL1 and CLOCK during 24 h in the sham group, the 6-OHDA-lesioned group, and the 6-OHDA-lesioned+L-dopa group.

**BMAL1**		**CLOCK**	
**Group**	**CV (%)**	**Group**	**CV (%)**
Sham	24.00	Sham	29.70
6-OHDA	13.60	6-OHDA	18.90
6-OHDA+L-dopa	22.10	6-OHDA+L-dopa	19.63

*L-dopa, L-3,4-dihydroxyphenylalanine*.

Then, the expressions of striatal BMAL1 in the 6-OHDA-lesioned group, the 6-OHDA-lesioned+L-dopa group, and the sham group were compared at the same time point. The Western blot analysis demonstrated that the expressions of BMAL1 significantly differed among these three groups (*p* < 0.05). As shown in Figure 4B, at all time points, except 16:00 and 24:00, the expressions of BMAL1 protein in the 6-OHDA-lesioned group were significantly lower than those of the sham group (*p* < 0.05). With the L-dopa administration, the expressions of BMAL1 protein recovered at 04:00, 08:00, and 12:00, compared with those of the 6-OHDA-lesioned groups. BMAL1 abundance in the 6-OHDA-lesioned side was reduced to 61.37 ± 5.30% of the sham saline-lesioned side at 04:00, 60.45 ± 5.95% at 08:00, and 65.88 ± 5.64% at 12:00, while in the 6-OHDA-lesioned+L-dopa group, striatal BMAL1 protein level increased to 102.8 ± 10.13% at 04:00, 85.52 ± 6.83% at 08:00, and 114.6 ± 10.43% at 12:00, respectively (*p* < 0.05).

**Figure 5:**
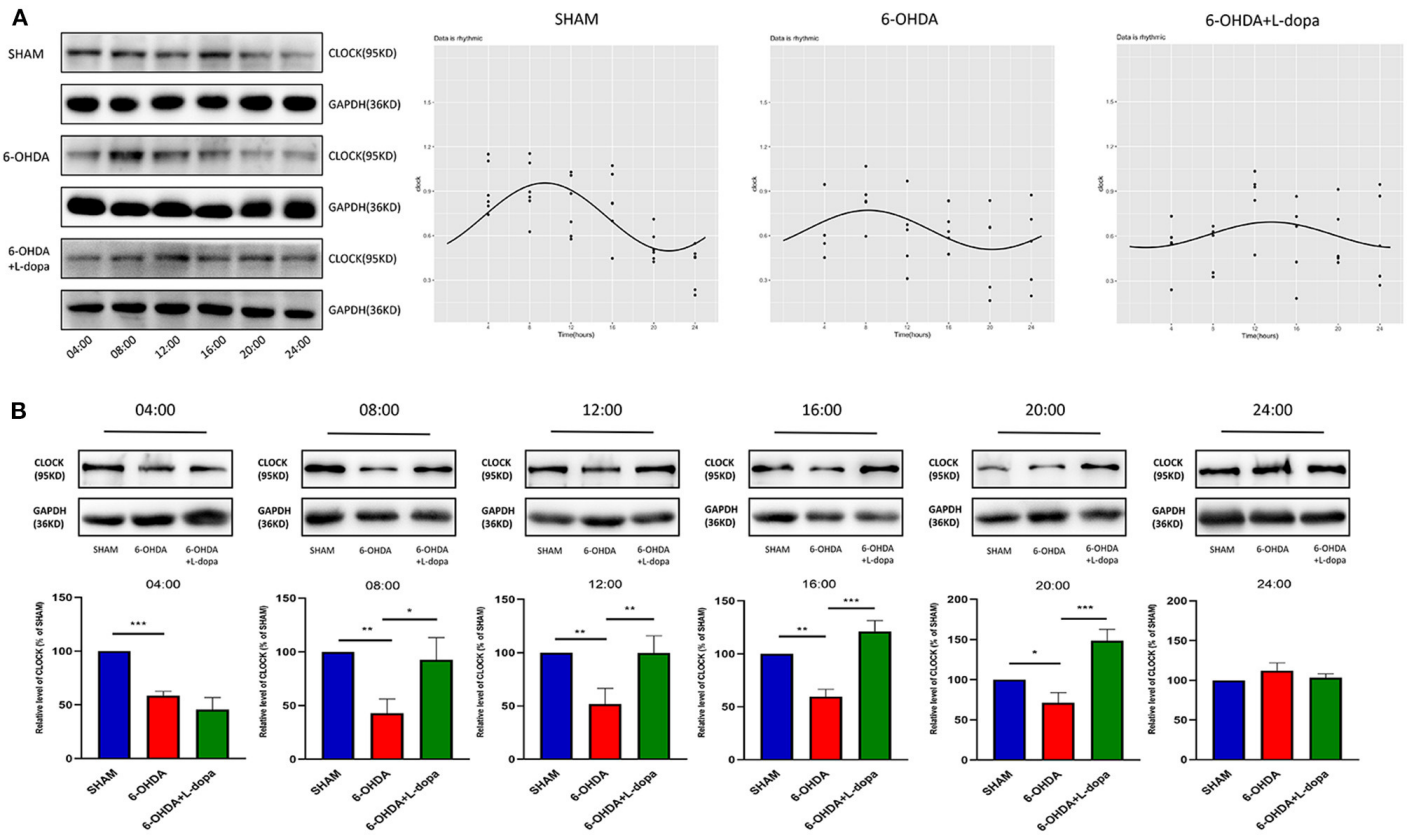
Western blot and the Cosinor analysis of CLOCK protein. **(A)** Western blot and the Cosinor analysis of CLOCK protein in sham, 6-OHDA-lesioned, and 6-OHDA-lesioned+L-dopa groups. The expressions of CLOCK protein among the sham group, 6-OHDA-lesioned group, and 6-OHDA+L-dopa group at different time points (every 4 h since 04:00). All the fitted curves were rhythmic fluctuated. **(B)** The expression of CLOCK protein at six time points during a day in sham, 6-OHDA-lesioned, and 6-OHDA+L-dopa groups. *p < 0.05. **p < 0.01, ***p < 0.001; *n* = 3–5 at each time point per group.

Then, the circadian rhythm alternations of the CLOCK protein in 24 h were explored among different groups. The expression of striatal CLOCK protein had its own time-dependent rhythm according to the Cosinor analysis (Figure 5A; Supplementary Table 2). The acrophases and the corresponding peak times of the three groups are shown in Table 3, and no statistical difference of the acrophases was found among these groups. The phase of the circadian rhythm of CLOCK protein was not altered by 6-OHDA or L-dopa administration. The data of variability showed that the CV was 29.70% in the sham group and was reduced to 18.90% by 6-OHDA intervention. L-dopa administration altered the CV to 19.60% (Table 4). Furthermore, we compared the expressions of CLOCK protein in these three groups at the same time points. Compared with sham saline-lesioned side, the expressions of striatal CLOCK protein significantly decreased to 58.63 ± 2.28% at 04:00, 42.95 ± 7.65% at 08:00, 51.58 ± 8.66% at 12:00, 59.55 ± 4.02% at 16:00, and 71.40 ± 7.19% at 20:00 in 6-OHDA-lesioned side (all *p* < 0.05). L-dopa treatment increased the expression of CLOCK at 08:00, 12:00, 16:00, and 20:00. CLOCK abundance recovered to 92.55 ± 12.01% at 08:00, 99.64 ± 9.38% at 12:00, 121.00 ± 6.00% at 16:00, and 148.4 ± 8.28% at 20:00 (all *p* < 0.05, Figure 5B).

These data suggested that 6-OHDA alters the circadian rhythms of core clock protein (i.e., BMAL1 and CLOCK) in the striatum of rats, predominantly by reducing the expressions rather than altering the phase. These could be partially reversed by L-dopa intervention.

### Role of the D1R-ERK1/2-mTOR Pathway on Changes of the Expressions of Striatal BMAL1 and CLOCK Protein

**Figure 6:**
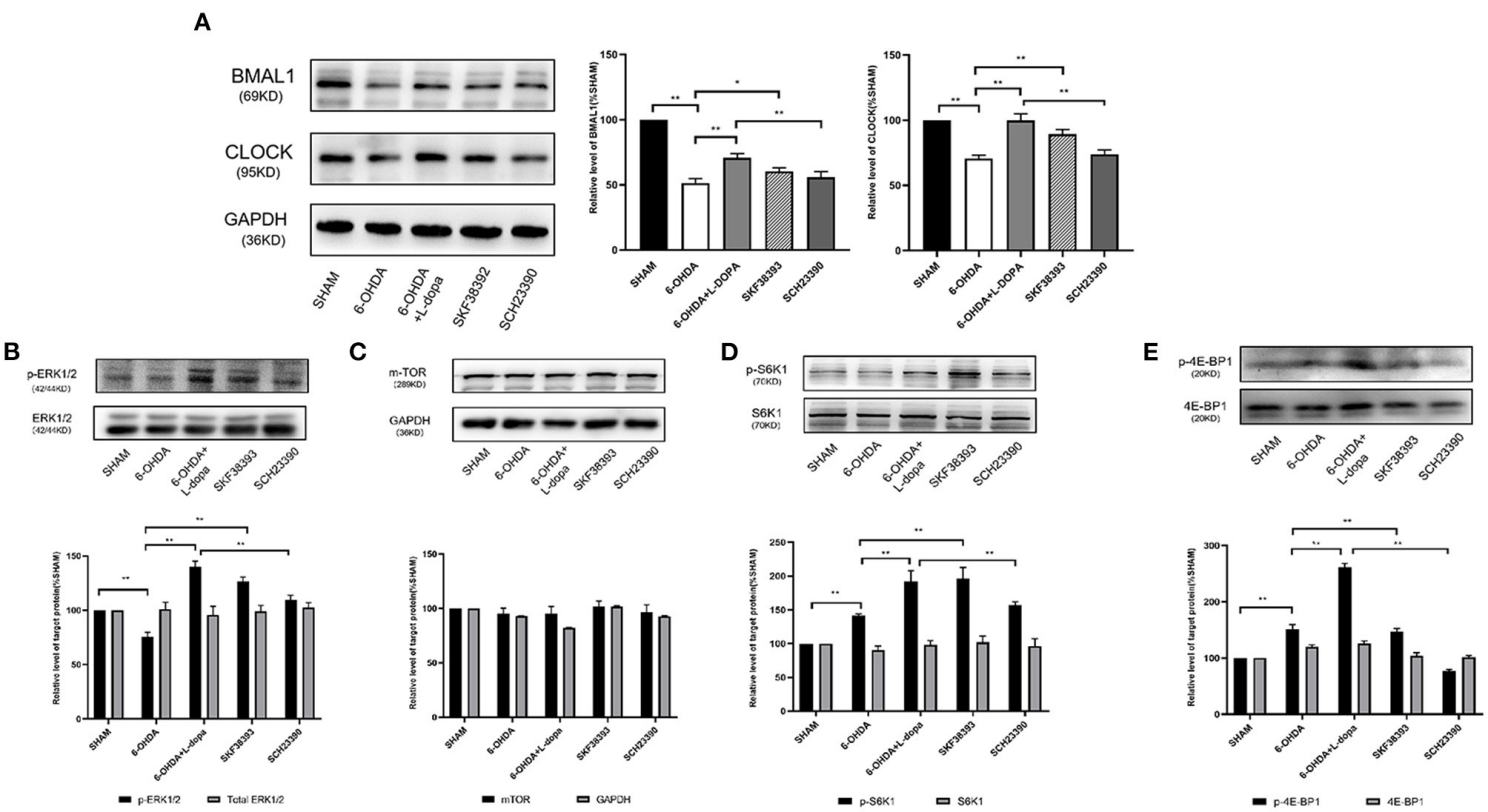
The effect of selective D1R agonist/antagonist on clock protein and downstream signaling. **(A)** The expressions of BMAL1 and CLOCK protein among five groups. Statistical analysis of the relative level of BMAL1 and CLOCK of sham saline-lesioned side. **(B–E)** The expression of ERK1/2, downstream effector of mTOR, and its phosphorylation levels among five groups. Western blot and statistical analysis of total ERK1/2 and phosphorylation of ERK1/2, mTOR, total S6K1 and phosphorylation of S6K1, as well as total 4E-BP1 and phosphorylation of 4E-BP1. *p < 0.05, **p < 0.01; *n* = 3–5 each group.

To determine whether the changes of the core clock protein induced by L-dopa were associated with the D1R-ERK1/2-mTOR pathway, experimental Part II was designed. Since there was no significant difference in the acrophase of core clock protein rhythm among sham, 6-OHDA-lesioned, and 6-OHDA+L-dopa groups, the striatum was simultaneously collected. As shown in Figure 6, compared with the sham group, the expressions of BMAL1 significantly decreased in the 6-OHDA-lesioned group (51.41 ± 1.97% of the sham saline-lesioned side). Compared with the 6-OHDA-lesioned group, the BMAL1 level was increased (to 70.79 ± 1.82% of the sham saline-lesioned side) in the 6-OHDA-lesioned+L-dopa group (*p* < 0.05) and increased to 60.52 ± 1.49% by D1R agonist SKF38393 (*p* < 0.05). However, SCH23390 significantly reduced the expressions of striatal BMAL1 increased by L-dopa (55.81 ± 2.60% of the sham saline-lesioned side in SCH23390 group vs. 70.79 ± 1.82% in the 6-OHDA-lesioned+L-dopa group, *p* < 0.05) (Figure 6A). The expression of striatal CLOCK protein was reduced to 70.68 ± 1.54% of the sham saline-lesioned side by 6-OHDA, and the L-dopa intervention recovered the level to 99.86 ± 2.95% on the sham saline-lesioned side (*p* < 0.05). Similar to L-dopa, SKF38393 elevated the expressions of CLOCK to 89.42 ± 2.07% of the sham saline-lesioned side, compared with the 6-OHDA-lesioned group (*p* < 0.05). In the SCH23390 group, SCH23390 significantly reduced the expression of striatal CLOCK increased by L-dopa (74.05 ± 1.80% of the sham saline-lesioned side in the SCH23390 group vs. 99.86 ± 2.95% in the 6-OHDA-lesioned+L-dopa group, *p* < 0.05) (Figure 6A).

The hyperactivity of the extracellular signal-regulated kinases 1/2 (ERK1/2) signaling is one of the major molecular changes in PD models treated by L-dopa. As shown in Figure 6B, the level of p-ERK1/2 was significantly higher in the 6-OHDA-lesioned+L-dopa group (140.10 ± 2.98% of the sham saline-lesioned side) and in the SKF38393 group (126.40 ± 2.50% of the sham saline-lesioned side) relative to the 6-OHDA-lesioned group (75.38 ± 2.54% of the sham saline-lesioned side). However, SCH23390 significantly decreased p-ERK1/2 level (109.80 ± 2.31% of the sham saline-lesioned side in SCH23390 group), which was elevated by L-dopa (*p* < 0.05). There was no difference in the expressions of total ERK1/2 among these groups (*p* > 0.05) (Figure 6B).

Ribosomal subunit S6 kinase 1 (S6K1) and the eukaryotic initiation factor 4E binding protein 1 (4E-BP1) are the important downstream effectors of mTOR, which regulate mitogen-stimulated translation initiation (Santini et al., 2009). The expressions of total mTOR, S6K1, and 4E-BP1 were similar among each group (*p* > 0.05) (Figures 6C–E). However, the phosphorylation level of S6K1 was significantly increased in the 6-OHDA-lesioned+L-dopa group (245.10 ± 0.46% of sham saline-lesioned side) and in the SKF38393 group (179.70 ± 5.26% of sham saline-lesioned side), compared with that in the 6-OHDA-lesioned group (141.60 ± 1.39% of the sham saline-lesioned side, *p* < 0.05). The abundance of p-S6K1 in the SCH23390 group (137.20 ± 2.83% of the sham saline-lesioned side) was significantly lower than that in the 6-OHDA-lesioned+L-dopa group (*p* < 0.05) and was close to that in the 6-OHDA-lesioned group (Figure 6D). The same change was observed for the expressions of p-4E-BP1 among these groups. Compared with the 6-OHDA-lesioned group (151.90 ± 4.43% of the sham saline-lesioned side), L-dopa (261.20 ± 4.22% of sham saline-lesioned side) or SKF38393 intervention (147.00 ± 3.24% of the sham saline-lesioned side) significantly increased the abundance of p-4E-BP1 (*p* < 0.05). SCH23390 (77.19 ± 1.53% of the sham saline-lesioned side) could reduce the hyperactive of p-4E-BP1 induced by L-dopa in 6-OHDA-lesioned rats (*p* < 0.05) (Figure 6E).

These data indicated that L-dopa could lead to a hyperactive D1R-mediated ERK1/2-mTOR signaling pathway in 6-OHDA-lesioned rats, which was associated with the changes of striatal BMAL1 and CLOCK protein.

## Discussion

This study confirmed that the circadian rhythm of core clock protein BMAL1 and CLOCK in the striatum was disturbed in 6-OHDA-lesioned rat models. Moreover, our data also indicated that this disturbance could be partially reversed by L-dopa, including the recovery of the protein expression and the variability of rhythms. Furthermore, our results demonstrated that the hyperactivation of the D1R-mediated ERK1/2-mTOR pathway was involved in the changes of striatal BMAL1 and CLOCK protein induced by L-dopa.

Although circadian rhythm activities are related to age, patients with PD frequently experience CRD, which is more severe than typical age-related CRD (Leng et al., 2019). So far, many studies have reported on multiple circadian rhythms in PD. Clinically, both motor and non-motor manifestations of PD show disruptions in typical 24-h oscillations (Li S. et al., 2017). Lin et al. showed reduced expression of BMAL1 in total leukocytes in patients with PD (Lin et al., 2012). Animal model studies also showed changes in circadian behavioral and physiological outputs in PD models and the expression of clock genes in different brain regions (Li S. Y. et al., 2017; De Lazzari et al., 2018; Wang et al., 2018). Consistent with these previous studies, we found that BP in the dark cycle of rats was reduced in rats with 6-OHDA. Moreover, the expressions of BMAL1 and CLOCK protein were downregulated in the striatum of 6-OHDA-lesioned rats at most time points of 24 h. Using the cosine analysis combined with the variability and semi-quantitative expression, our data further indicated that the circadian rhythms were flattened due to the loss of dopaminergic neurons after 6-OHDA, while the rhythm phases remained unchanged. This was consistent with other reports suggesting that the amplitude of circadian rhythm disorder was reduced in PD, and the circadian phases had no significant shift (Berganzo et al., 2013; Zhong et al., 2013; Bolitho et al., 2014; Breen et al., 2014; Tholfsen et al., 2015).

Importantly, the results of this study demonstrated that L-dopa therapy recovered the expression levels of BMAL1 and CLOCK and the variability of rhyme caused by 6-OHDA, without significant alterations of the rhythm phase. Although less information focused on the L-dopa effect on CRD in PD models, recent research (Li S. Y. et al., 2017) has shown that L-dopa treatment exacerbates the disruptions of circadian rhythm in 6-OHDA-lesioned rats, which was not consistent with our results. Another research revealed that L-dopa treatment might improve or accelerate the recovery of circadian rhythmicity (Boulamery et al., 2010). The effects of L-dopa in PD models were contrary to those imposed by 6-OHDA lesions (Ben and Bruguerolle, 2000), and L-dopa was considered to correct to some extent the alterations of parameters of circadian rhythmicity (Pérez-Lloret and Cardinali, 2021). L-dopa is the most effective treatment for PD, while the symptoms related to CRD are important in the spectrum of PD profiles. Therefore, it is necessary to clarify the relationship between L-dopa and CRD symptoms in PD. Our results indicated that L-dopa positively affects circadian rhythm disruption of striatal core clock protein in PD rats. In circadian behavioral parameters, L-dopa treatment could also recover the CV of SBP or DBP reduced by 6-OHDA. These findings imply that the neurodegeneration progression itself might contribute more to the CRD of PD than L-dopa therapy.

Our previous study indicated that chronic L-dopa treatment leads to abnormal overexpression of D1R in the striatum and, in turn, the activation of the D1R-mediated mTOR downstream pathway (Wu et al., 2018). mTOR has a key role in regulating circadian rhythms in various tissues and cells (Hughes et al., 2009; Zhang et al., 2014). Once the mTOR is inhibited, the period of circadian rhythm is extended, and the amplitude is shortened, while its activation shortens the period and increases the amplitude in peripheral clock models of hepatocytes and adipocytes (Ramanathan et al., 2018). Furthermore, the genetic alterations of mTOR and its downstream signaling components have been shown to affect circadian rhythms in *Drosophila* and mice (Zheng and Sehgal, 2010; Cao et al., 2013; Kijak and Pyza, 2017; Liu et al., 2018). Under this background, we hypothesized that L-dopa ameliorated the circadian rhythm disorders of core clock protein in the striatum of the PD model, which might be due to the hyperactivation of the D1R-ERK1/2-mTOR signaling pathway induced by L-dopa.

Our data suggested that L-dopa treatment induced the hyperphosphorylation of ERK1/2, S6K1, and 4E-BP1 in the 6-OHDA-lesioned striatum. The latter two proteins were the important downstream effectors of the mTOR pathway regulating mitogen-stimulated translation initiation (Liu et al., 2018). This hyperactivation was similar to the effects of D1R agonist SKF38393, while the coadministration of L-dopa and D1R antagonist SCH23390 did not induce the hyperphosphorylation of these proteins. These data demonstrated that L-dopa administration in the PD model could lead to the overactivation of signal molecules in the D1R-mediated ERK1/2-mTOR pathway, which was consistent with the previous studies (Santini et al., 2009; Decressac and Björklund, 2013; Wu et al., 2018). Interestingly, the change of the expression of BMAL1 and CLOCK in 6-OHDA-lesioned striatum+L-dopa was consistent with the change of main factors in the mTOR pathway mediated by D1R. This indicated that core clock protein was related to the mTOR pathway. There is considerable evidence for the role of mTOR signaling in the regulation of the circadian clock (Ramanathan et al., 2018). The mTOR activities have been found to show daily rhythms in various tissues, and a certain level of mTOR activities is required to maintain normal circadian rhythms (Cao and Obrietan, 2010). In the SCN, the activity of the mTOR pathway was reported to be stimulated by light, while its downstream protein 4E-BP regulated the circadian timing (Cao et al., 2013). The mTOR promoted the expression of BMAL1 protein and proteostasis, while the circadian rhythms of core clock genes were altered when the mTOR pathway was abnormal (Lipton et al., 2017). However, it remained unclear how mTOR signaling regulated BMAL1. As previous reports showed that BMAL1 was phosphorylated by S6K1 to promote translation and protein synthesis (Lipton et al., 2015), we supposed that the hyperactivation of mTOR signaling (e.g., the overexpression of p-S6K1) regulated the BMAL1 phosphorylation and further promoted its translation, elevated its protein expressions, and enhanced its function. Nonetheless, future studies are needed to further elucidate these mechanisms. The presented data suggested that the hyperactivity of the D1R-ERK1/2-mTOR pathway induced by the D1R activation contributed to the recovery of circadian rhythms of clock protein caused by dopamine deficiency.

This study has a few limitations. First, we only observed the BP and temperature as CRD symptoms; thus, other CRD-related symptoms need to be analyzed to clarify the response to L-dopa. Second, the manifestations of CRD in the 6-OHDA rat model may not fully simulate that of patients with PD. Further clinical and experimental evidence of CRD response to L-dopa in PD is needed to support our results.

## Conclusion

Our data indicated that L-dopa could recover CRD in PD rats by regulating the D1R-ERK1/2-mTOR pathway. These results may be useful for the treatment of PD. L-dopa is still considered the most effective therapy for PD. L-dopa combined with rhythm modulation therapy might be the potential method for restoring circadian arrhythmicity and ameliorating some CRD. Still, further molecular evidence and studies on the L-dopa effect on circadian dysregulation in PD are needed.

## Data Availability Statement

The original contributions presented in the study are included in the article/Supplementary Material, further inquiries can be directed to the corresponding authors.

## Ethics Statement

The animal study was reviewed and approved by the Institutional Review Board of Xinhua Hospital affiliated to Jiaotong University, School of Medicine, Shanghai.

## Author Contributions

JG and ZheL designed the research and searched databases. SY, ZhiL, and YL performed the research and wrote the paper. YW, LS, NW, and JZ analyzed the data and revised the manuscript. All authors read and approved the final manuscript.

## Conflict of Interest

The authors declare that the research was conducted in the absence of any commercial or financial relationships that could be construed as a potential conflict of interest.

## Publisher's Note

All claims expressed in this article are solely those of the authors and do not necessarily represent those of their affiliated organizations, or those of the publisher, the editors and the reviewers. Any product that may be evaluated in this article, or claim that may be made by its manufacturer, is not guaranteed or endorsed by the publisher.
