# Supplementary material for: L-3,4-Dihydroxyphenylalanine Recovers Circadian Rhythm Disturbances in the Rat Models of Parkinson's Disease by Regulating the D1R-ERK1/2-mTOR Pathway
Source: Front Aging Neurosci. 2021 Aug 19;13:719885. doi: 10.3389/fnagi.2021.719885 (PMC8417416; doi:10.3389/fnagi.2021.719885)
Supplement: Supplementary file 1 [file Image_1.pdf]

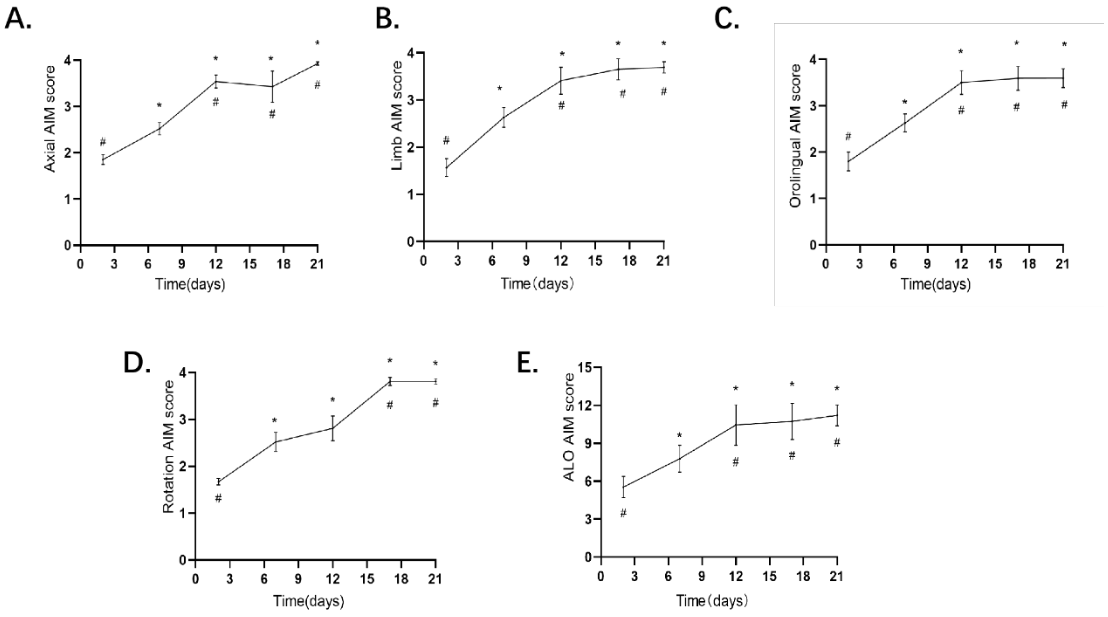

Figure Supplementary 1. The AIM scores assessment. In 6-OHDA-lesined + L-dopa group, AIM scores were assessed on day2， day7， day12， day17 and day21 of L-dopa treatment, scored every 20 min on a scale of 0-4 for 120 min.(A-E): Correlation analysis of the axial, limb, orolingual and the sum of scores relative to time of L-dopa treatment. (F): Correlation analysis of sum score of axial, limb and orolingual (ALO) abnormal involuntary movement (AIM) relative to time of L-dopa treatment. All values were shown in Mean± SEM; *p<0.05 vs day2, # p<0.05 vs day7. n=24.
