# Supplementary material for: L-3,4-Dihydroxyphenylalanine Recovers Circadian Rhythm Disturbances in the Rat Models of Parkinson's Disease by Regulating the D1R-ERK1/2-mTOR Pathway
Source: Front Aging Neurosci. 2021 Aug 19;13:719885. doi: 10.3389/fnagi.2021.719885 (PMC8417416; doi:10.3389/fnagi.2021.719885)
Supplement: Supplementary file 2 [file Table_1.DOCX]

| **Systolic blood pressure (SBP)** | | | | | | |
| --- | --- | --- | --- | --- | --- | --- |
| Time | Sham | 6-OHDA | 6-OHDA+  L-dopa | P_1_ | P_2_ | P_3_ |
| 08:00 | 97.95±2.55 | 104.39±2.11 | 104.80±2.03 | 0.2071 | 0.2003 | 0.9946 |
| 10:00 | 101.17±1.82 | 105.00±1.95 | 106.71±1.90 | 0.7803 | 0.6764 | 0.9464 |
| 12:00 | 106.17±1.07 | 104.39±2.65 | 111.53±2.49 | 0.9541 | 0.6156 | 0.301 |
| 14:00 | 112.00±8.38 | 106.85±2.68 | 113.20±2.27 | 0.4831 | 0.9664 | 0.2381 |
| 16:00 | 120.83±5.30 | 104.44±2.20 | 106.53±2.08 | 0.0055* | 0.0017* | 0.921 |
| 18:00 | 130.44±5.52 | 105.33±1.94 | 113.00±2.19 | <0.0001* | 0.0001* | 0.1523 |
| 20:00 | 132.88±3.15 | 119.13±3.13 | 131.33±2.72 | 0.0004* | 0.9269 | 0.0134* |
| 22:00 | 144.81±2.65 | 109.47±1.73 | 106.00±2.12 | <0.0001* | <0.0001* | 0.6924 |
| 24:00 | 147.14±3.00 | 102.59±2.02 | 110.36±1.95 | <0.0001* | <0.0001* | 0.1558 |
| 02:00 | 146.32±3.00 | 100.78±1.81 | 111.00±2.64 | <0.0001* | <0.0001* | 0.0433* |
| 04:00 | 144.86±3.29 | 99.00±1.73 | 112.40±3.10 | <0.0001* | <0.0001* | 0.0031* |
| 06:00 | 103.50±1.32 | 104.89±2.02 | 108.29±1.72 | 0.9477 | 0.5475 | 0.6954 |

| **Diastolic blood pressure (DBP)** | | | | | | | | | | | | |  |  |
| --- | --- | --- | --- | --- | --- | --- | --- | --- | --- | --- | --- | --- | --- | --- |
| Time | | Sham | | 6-OHDA | | 6-OHDA+  L-dopa | | P_1_ | P_2_ | | | P_3_ |  |  |
| 08:00 | | 53.48±3.02 | | 60.65±2.29 | | 43.53±2.28 | | 0.2206 | 0.0734 | | | 0.0008* |  |  |
| 10:00 | | 60.67±2.30 | | 54.29±1.96 | | 51.14±1.06 | | 0.5874 | 0.4094 | | | 0.8652 |  |  |
| 12:00 | | 66.17±1.35 | | 52.39±3.34 | | 59.53±2.99 | | 0.1162 | 0.562 | | | 0.3942 |  |  |
| 14:00 | | 78.20±7.00 | | 46.55±3.51 | | 50.07±3.02 | | <0.0001* | <0.0001* | | | 0.7093 |  |  |
| 16:00 | | 84.33±4.34 | | 47.25±2.51 | | 52.13±3.79 | | <0.0001* | <0.0001* | | | 0.7068 |  |  |
| 18:00 | | 98.13±3.64 | | 46.11±3.58 | | 57.67±3.88 | | <0.0001* | <0.0001* | | | 0.0375* |  |  |
| 20:00 | | 101.29±2.58 | | 66.38±3.40 | | 81.83±4.76 | | <0.0001* | 0.0001* | | | 0.0049* |  |  |
| 22:00 | | 105.38±3.22 | | 65.47±2.94 | | 68.87±2.40 | | <0.0001* | <0.0001* | | | 0.7608 |  |  |
| 24:00 | | 107.14±2.61 | | 54.94±3.94 | | 56.21±2.85 | | <0.0001* | <0.0001* | | | 0.9619 |  |  |
| 02:00 | | 109.32±4.01 | | 48.22±2.62 | | 54.87±2.84 | | <0.0001* | <0.0001* | | | 0.353 |  |  |
| 04:00 | | 102.18±4.04 | | 52.13±3.33 | | 49.20±3.36 | | <0.0001* | <0.0001* | | | 0.8026 |  |  |
| 06:00 | | 45.08±1.73 | | 52.44±1.17 | | 45.43±3.00 | | 0.3141 | 0.9975 | | | 0.3043 |  |  |
|  | |  | |  | |  | |  |  | | |  |  |  |
| **Temperature** | | | | | | | | | | | | | | |
| Time | | Sham | | 6-OHDA | | 6-OHDA+L-dopa | | P_1_ | | | P_2_ | P_3_ | | |
| 08:00 | | 36.34±0.16 | | 37.62±0.11 | | 35.96±0.65 | | 0.0067* | | | 0.6656 | 0.0014* | | |
| 10:00 | | 36.34±0.09 | | 36.73±0.12 | | 35.88±0.48 | | 0.6759 | | | 0.6122 | 0.162 | | |
| 12:00 | | 36.40±0.07 | | 36.00±0.50 | | 36.44±0.07 | | 0.6669 | | | 0.9963 | 0.6128 | | |
| 14:00 | | 36.10±0.07 | | 36.26±0.56 | | 35.6±0.52 | | 0.9421 | | | 0.5603 | 0.3659 | | |
| 16:00 | | 35.65±0.19 | | 36.74±0.09 | | 36.42±0.32 | | 0.0122* | | | 0.1634 | 0.7539 | | |
| 18:00 | | 36.36±0.36 | | 36.76±0.27 | | 37.025±0.62 | | 0.6051 | | | 0.2882 | 0.8368 | | |
| 20:00 | | 36.64±0.28 | | 36.04±0.38 | | 36.08±0.42 | | 0.3385 | | | 0.4256 | 0.9959 | | |
| 22:00 | | 36.49±0.43 | | 37.13±0.29 | | 36.42±0.48 | | 0.259 | | | 0.9883 | 0.2552 | | |
| 24:00 | | 36.37±0.56 | | 36.67±0.37 | | 36.58±0.28 | | 0.7632 | | | 0.9033 | 0.9823 | | |
| 02:00 | | 36.15±0.05 | | 37.62±0.10 | | 36.54±0.21 | | 0.0099* | | | 0.6862 | 0.0277* | | |
| 04:00 | | 36.1±0.15 | | 37.40±0.11 | | 36.46±0.09 | | 0.0025* | | | 0.6182 | 0.0408* | | |
| 06:00 | | 36.15±0.13 | | 36.70±0.14 | | 36.36±0.36 | | 0.423 | | | 0.8814 | 0.7644 | | |

Table Supplementary 1. Details of SBP, DBP and Temperature in Sham group, 6-OHDA-lesioned group and 6-OHDA-lesioned + L-dopa group. P1: Sham vs. 6-OHDA-lesioned group, P2: Sham vs.6-OHDA-lesioned + L -dopa group, P3: 6-OHDA-lesioned group vs. 6-OHDA-lesioned + L -dopa group.

| BMAL1 | | | CLOCK | | |
| --- | --- | --- | --- | --- | --- |
| Group | Amplitude  （95% confidence interval） | MESOR | Group | Amplitude  （95% confidence interval） | MESOR |
| Sham | 0.1051 (-0.0089 to 0.2192) | 0.7483 | Sham | 0.2293 (0.1308 to 0.3277) | 0.7265 |
| 6-OHDA | 0.1084 (-0.0074 to 0.2243) | 0.7011 | 6-OHDA | 0.1323(0.0116 to 0.2530) | 0.6391 |
| 6-OHDA  +L-dopa | 0.1887 (-0.0387 to 0.3387) | 0.7641 | 6-OHDA  +L-dopa | 0. 0854(-0.0404 to 0.2113) | 0.6086 |

Table Supplementary 2. The amplitude (95% confidence interval) and mesor of BMAL1 and CLOCK protein expression in Sham group, 6-OHDA-lesioned group and 6-OHDA-lesioned + L-dopa group. The 95% confidence interval of the amplitude does not overlap the MESOR demonstrates that the data conform to about a 24-h rhythmic pattern (Refinetti, Lissen et al. 2007).

Refinetti, R., G. C. Lissen and F. Halberg (2007). "Procedures for numerical analysis of circadian rhythms." Biol Rhythm Res **38**(4): 275-325. doi: 10.1080/09291010600903692.
