# Supplementary figures and images for: L-3,4-Dihydroxyphenylalanine Recovers Circadian Rhythm Disturbances in the Rat Models of Parkinson's Disease by Regulating the D1R-ERK1/2-mTOR Pathway
Source: Front Aging Neurosci. 2021 Aug 19;13:719885. doi: 10.3389/fnagi.2021.719885 (PMC8417416; doi:10.3389/fnagi.2021.719885)

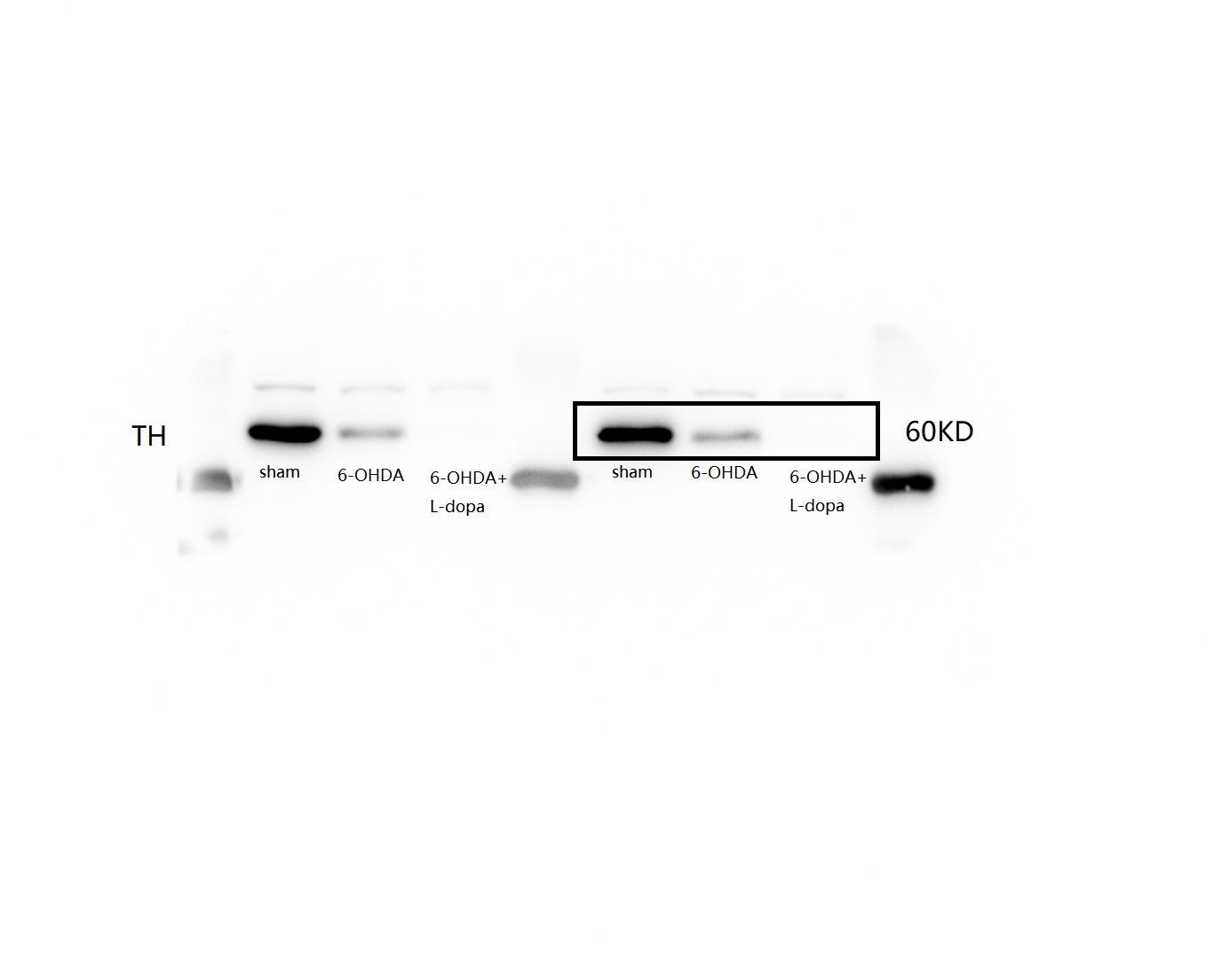

Supplement: Supplementary file 3 [file Data_Sheet_1.ZIP › full scan of the entire original gels/Figure2/TH.tif]

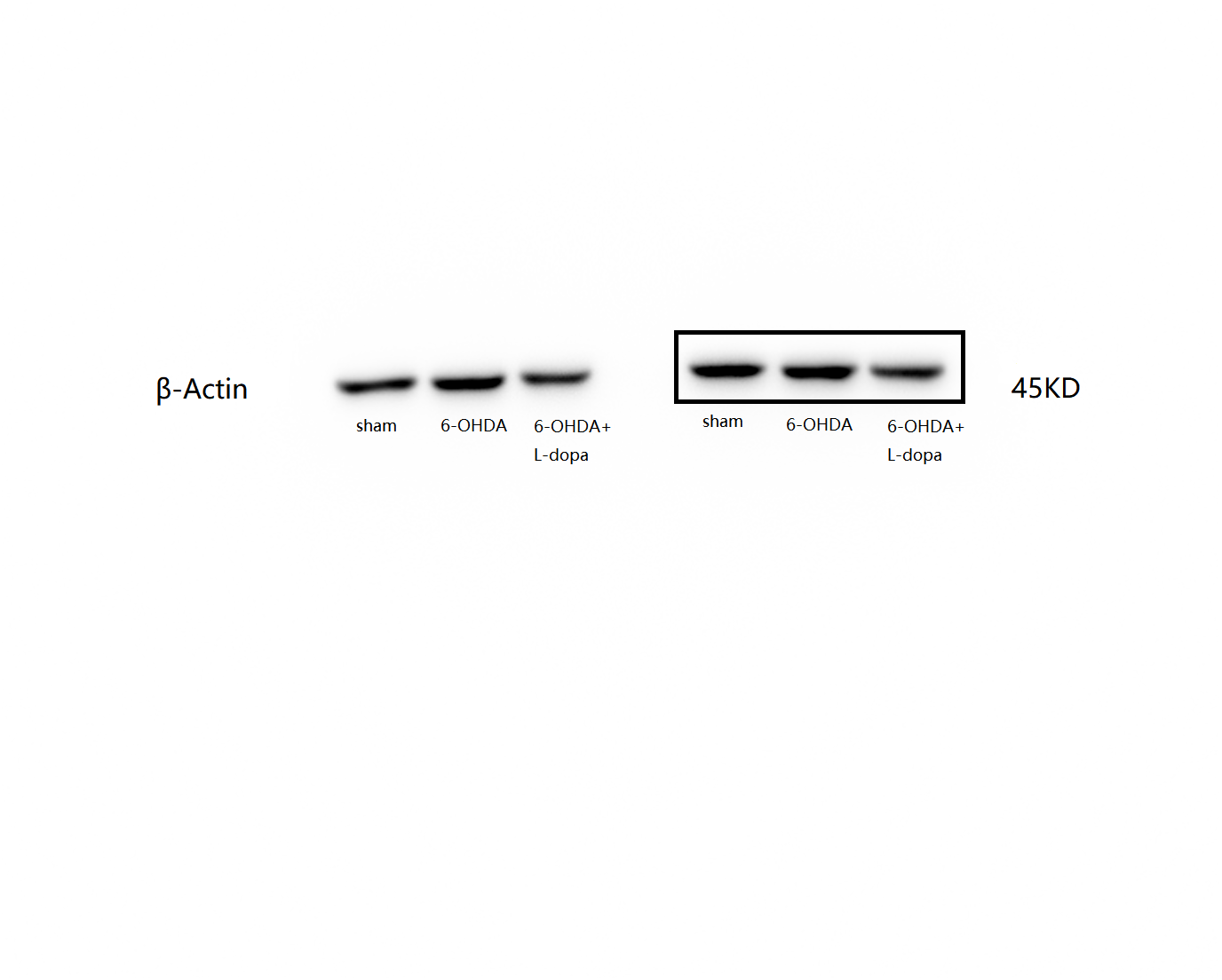

Supplement: Supplementary file 3 [file Data_Sheet_1.ZIP › full scan of the entire original gels/Figure2/β-ACTIN.tif]

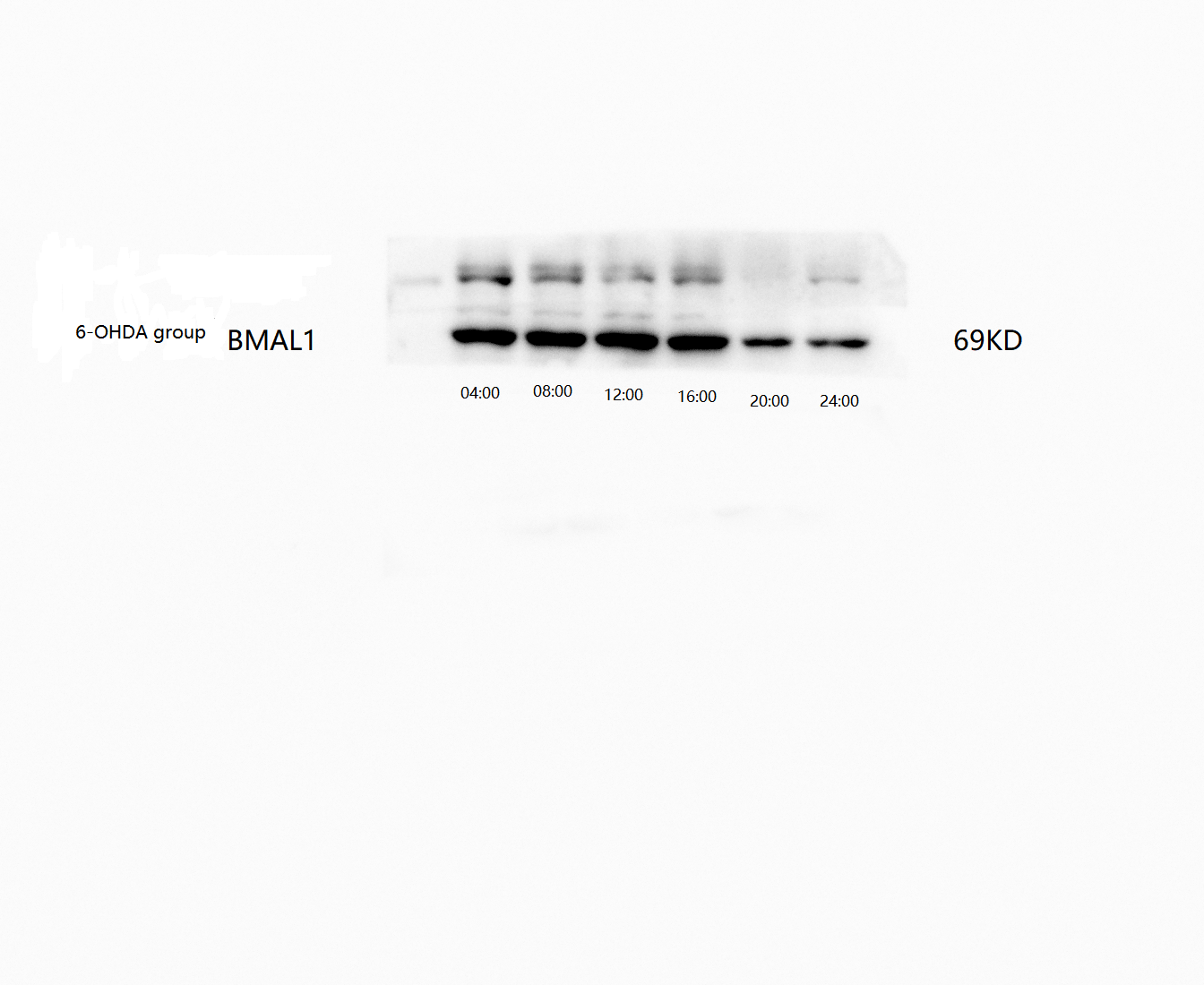

Supplement: Supplementary file 3 [file Data_Sheet_1.ZIP › full scan of the entire original gels/Figure4vs5/BMAL1/A/6-OHDA/BMAL1-6-OHDA.tif]

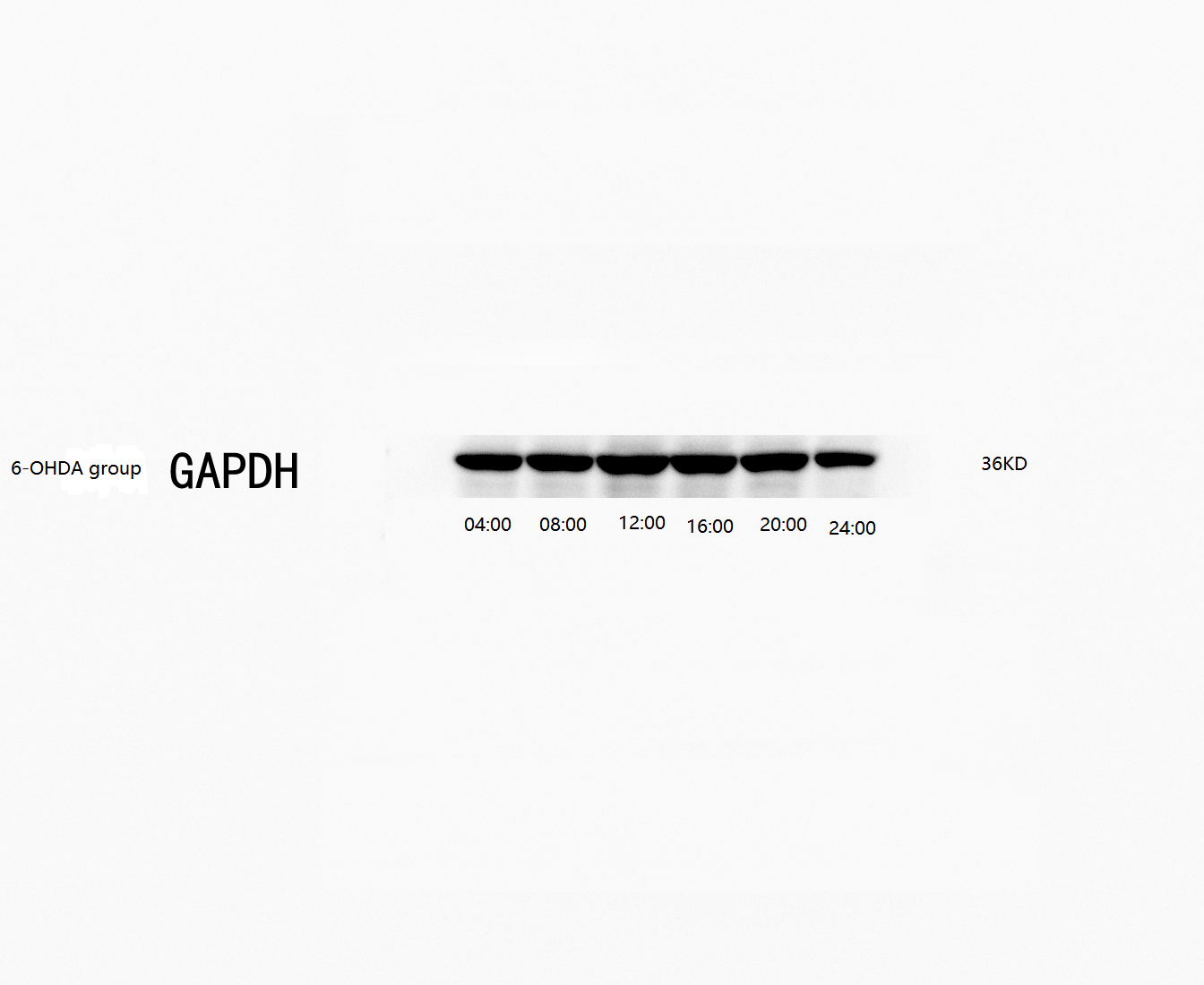

Supplement: Supplementary file 3 [file Data_Sheet_1.ZIP › full scan of the entire original gels/Figure4vs5/BMAL1/A/6-OHDA/GAPDH-6-OHDA.tif]

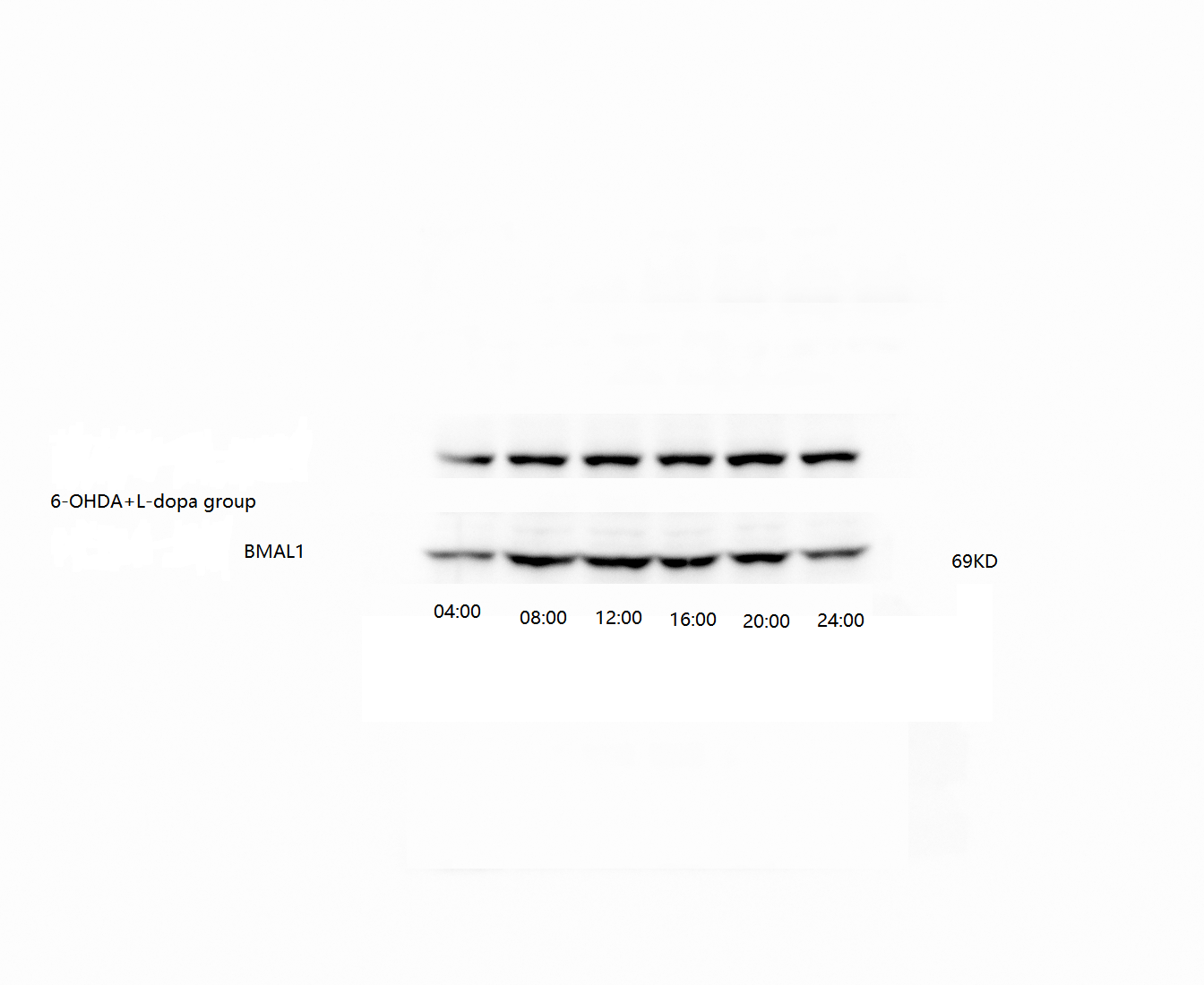

Supplement: Supplementary file 3 [file Data_Sheet_1.ZIP › full scan of the entire original gels/Figure4vs5/BMAL1/A/6-OHDA+L-dopa/6-OHDA+L-dopa-BMAL1.tif]

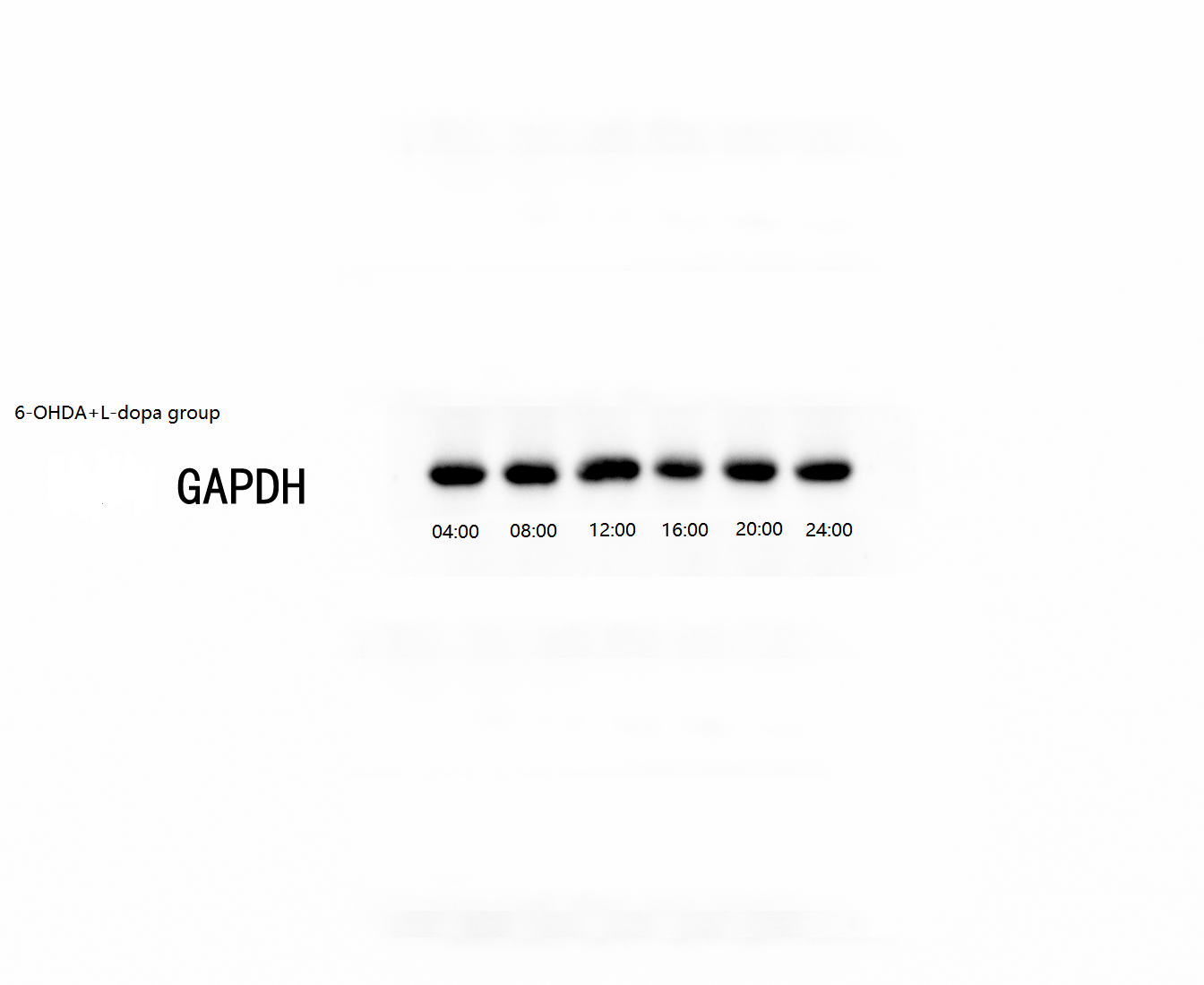

Supplement: Supplementary file 3 [file Data_Sheet_1.ZIP › full scan of the entire original gels/Figure4vs5/BMAL1/A/6-OHDA+L-dopa/6-OHDA+L-dopa-GAPDH.tif]

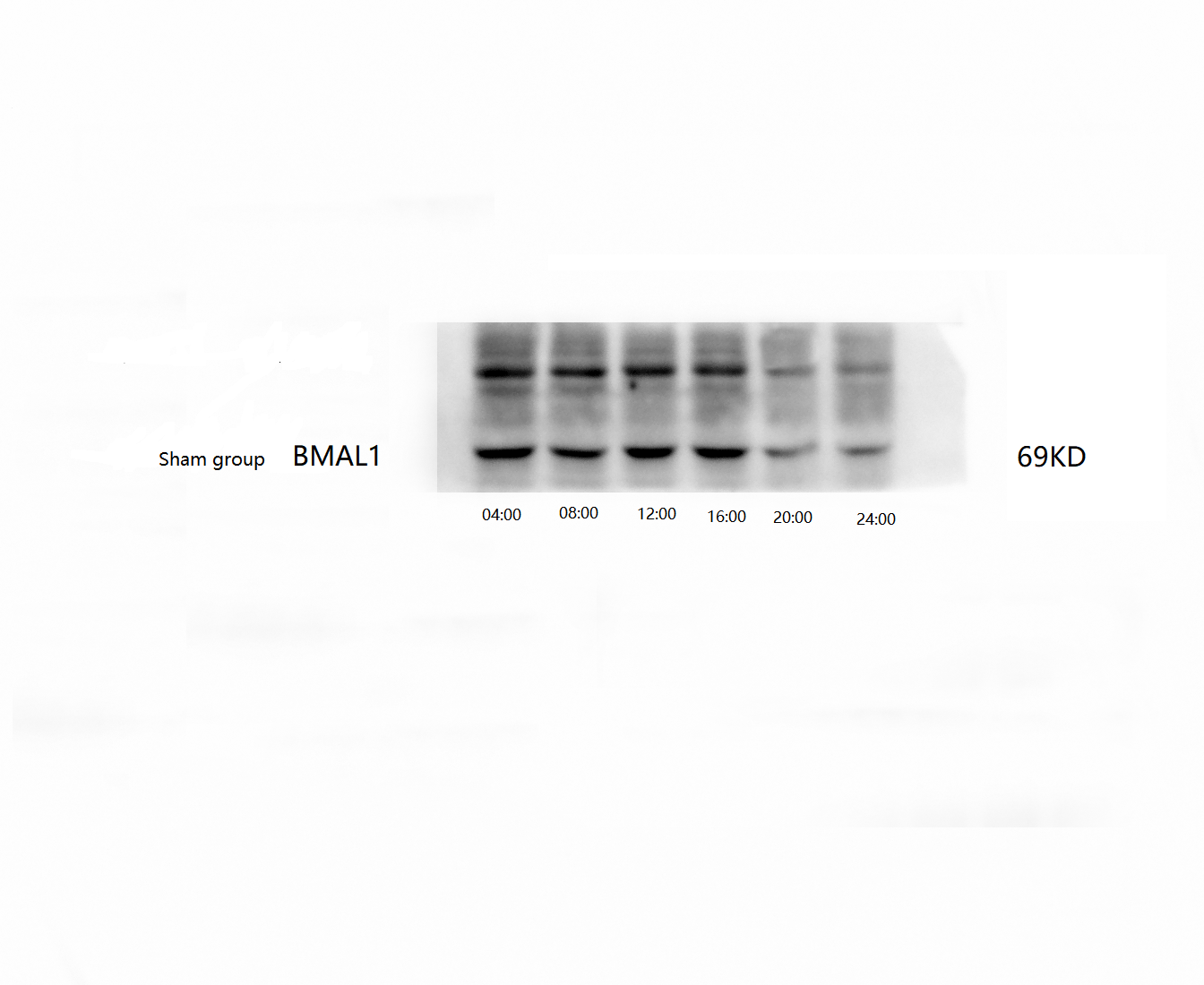

Supplement: Supplementary file 3 [file Data_Sheet_1.ZIP › full scan of the entire original gels/Figure4vs5/BMAL1/A/SHAM/BMAL1-SHAM.tif]

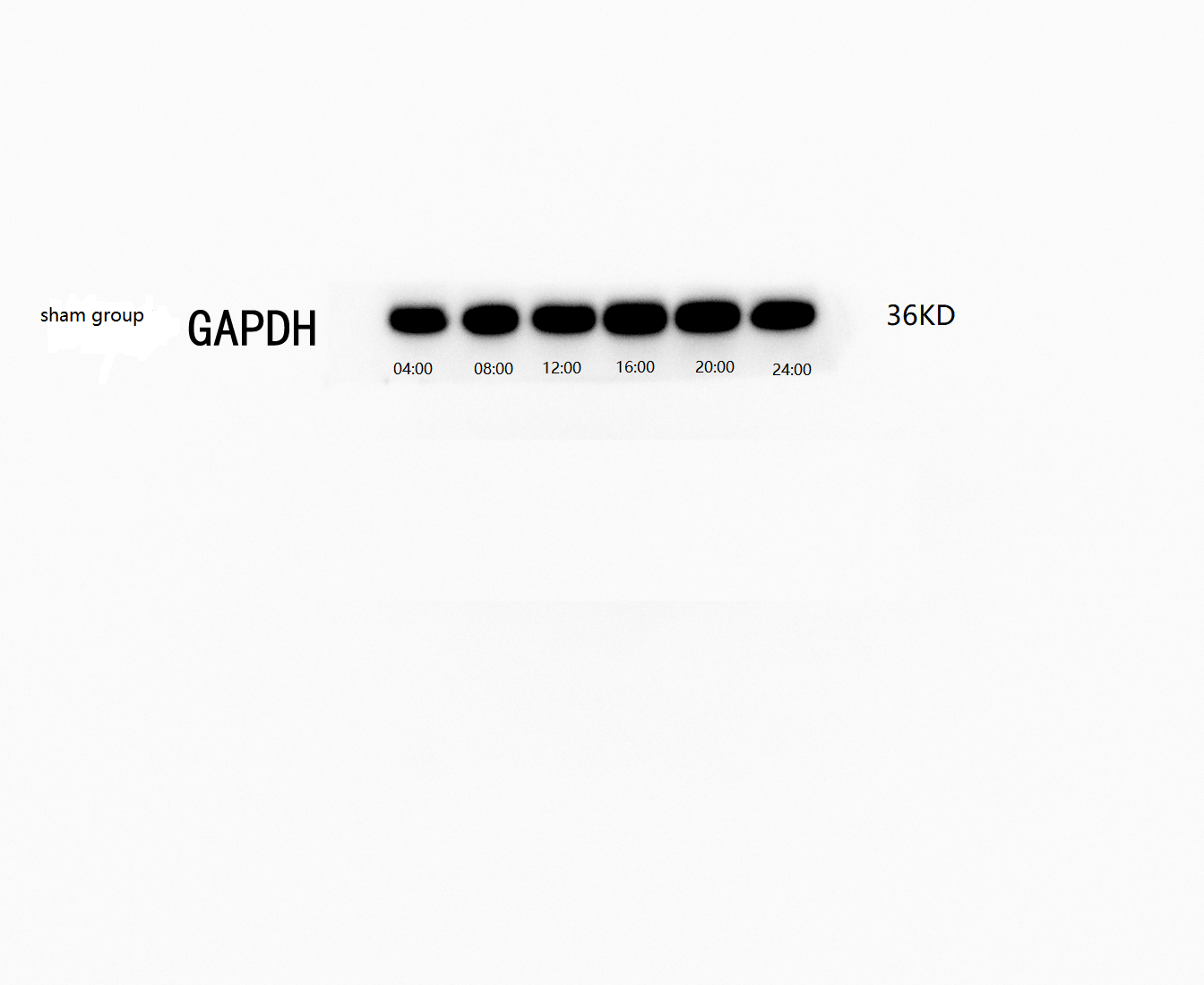

Supplement: Supplementary file 3 [file Data_Sheet_1.ZIP › full scan of the entire original gels/Figure4vs5/BMAL1/A/SHAM/GAPDH-SHAM.tif]

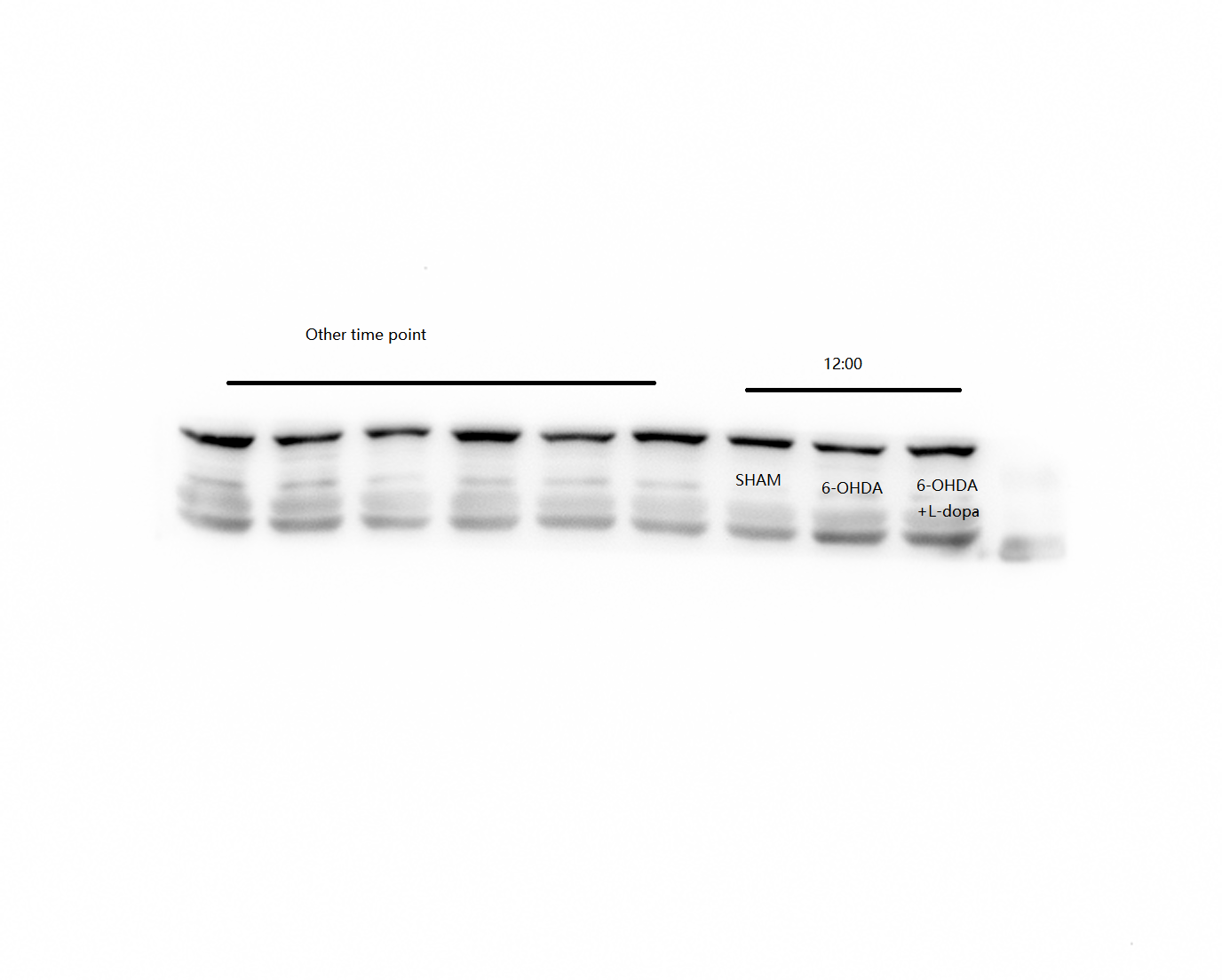

Supplement: Supplementary file 3 [file Data_Sheet_1.ZIP › full scan of the entire original gels/Figure4vs5/BMAL1/B/12H/bmal1.tif]

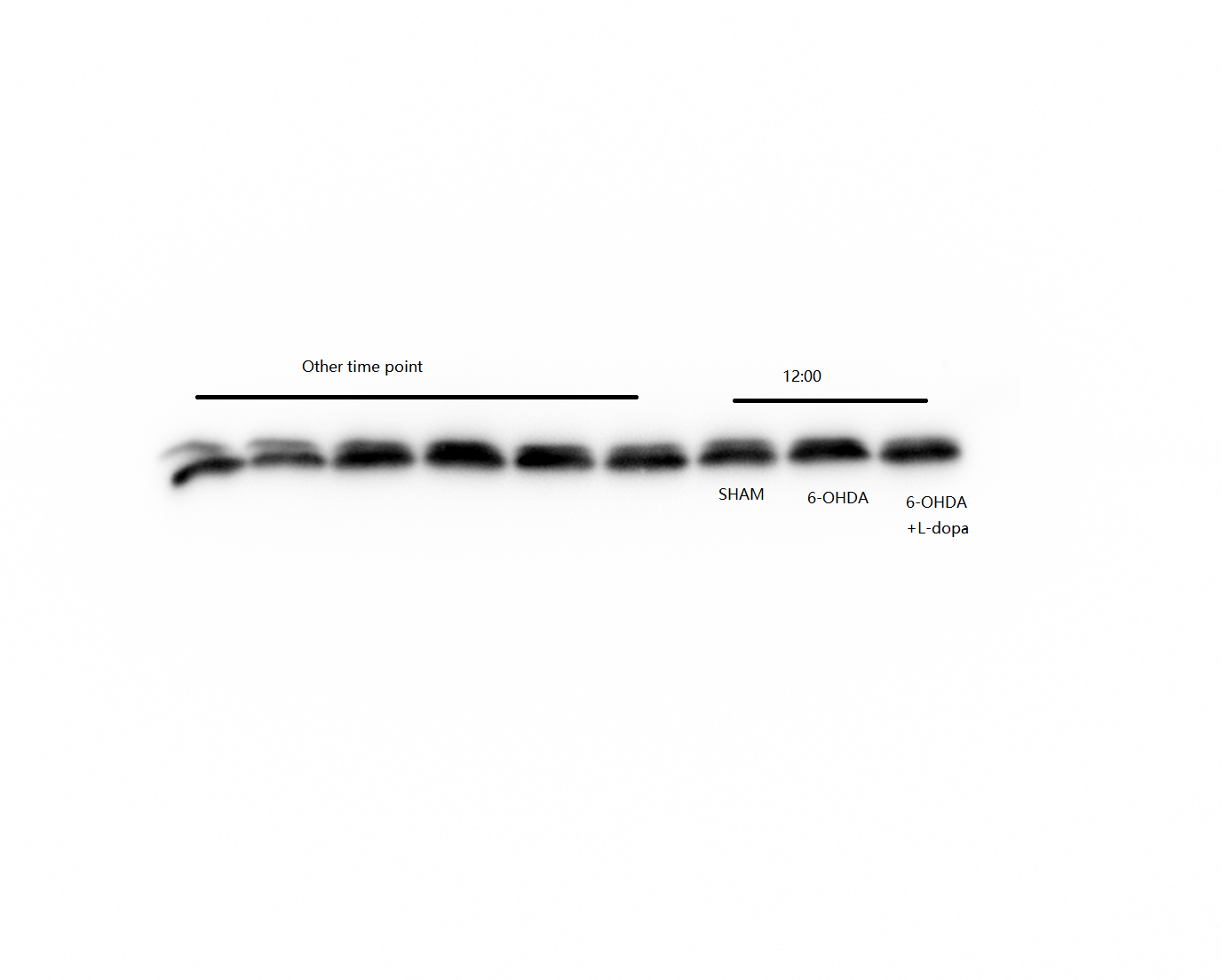

Supplement: Supplementary file 3 [file Data_Sheet_1.ZIP › full scan of the entire original gels/Figure4vs5/BMAL1/B/12H/GAPDH.tif]

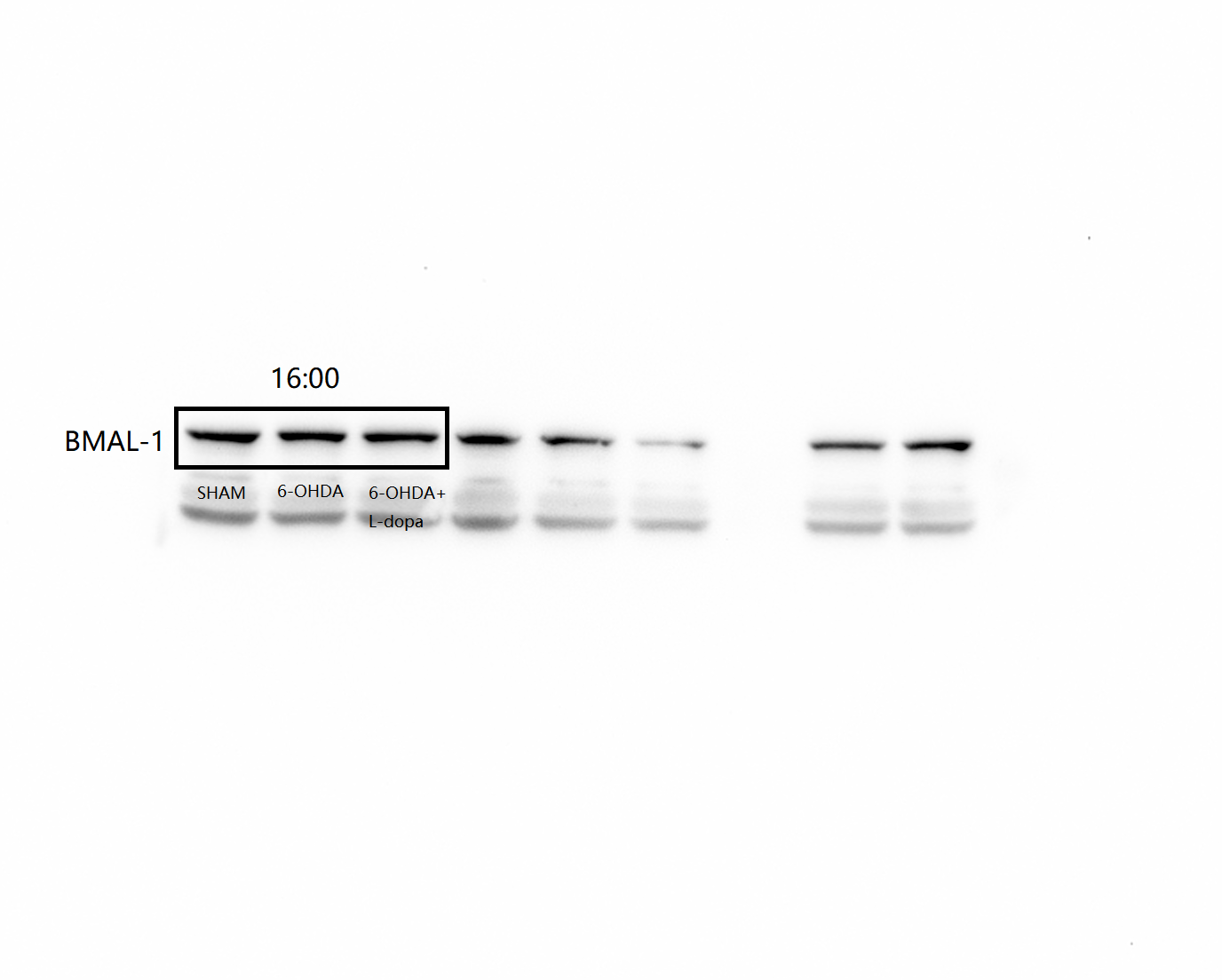

Supplement: Supplementary file 3 [file Data_Sheet_1.ZIP › full scan of the entire original gels/Figure4vs5/BMAL1/B/16H/BMAL1.tif]

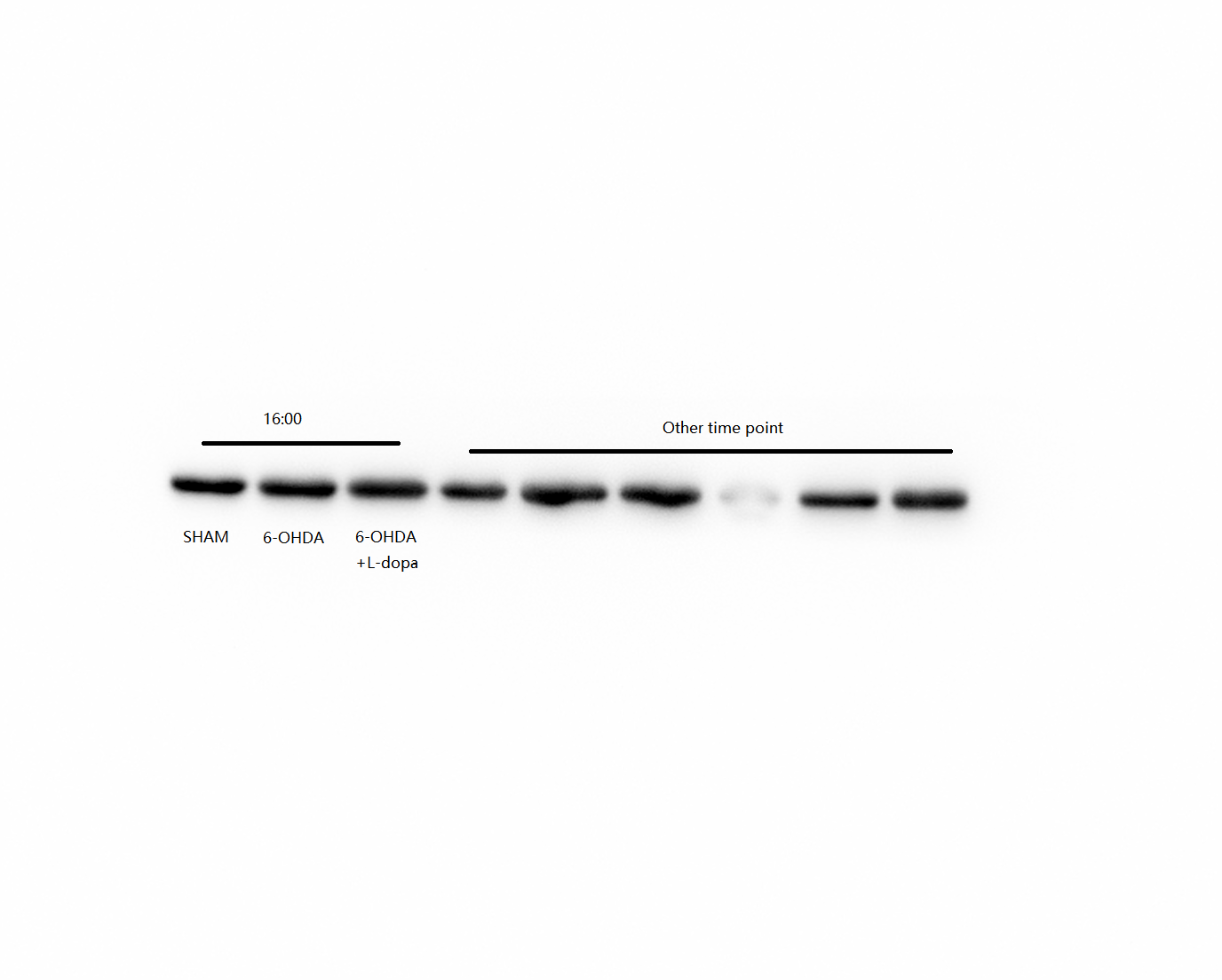

Supplement: Supplementary file 3 [file Data_Sheet_1.ZIP › full scan of the entire original gels/Figure4vs5/BMAL1/B/16H/GAPDH.tif]

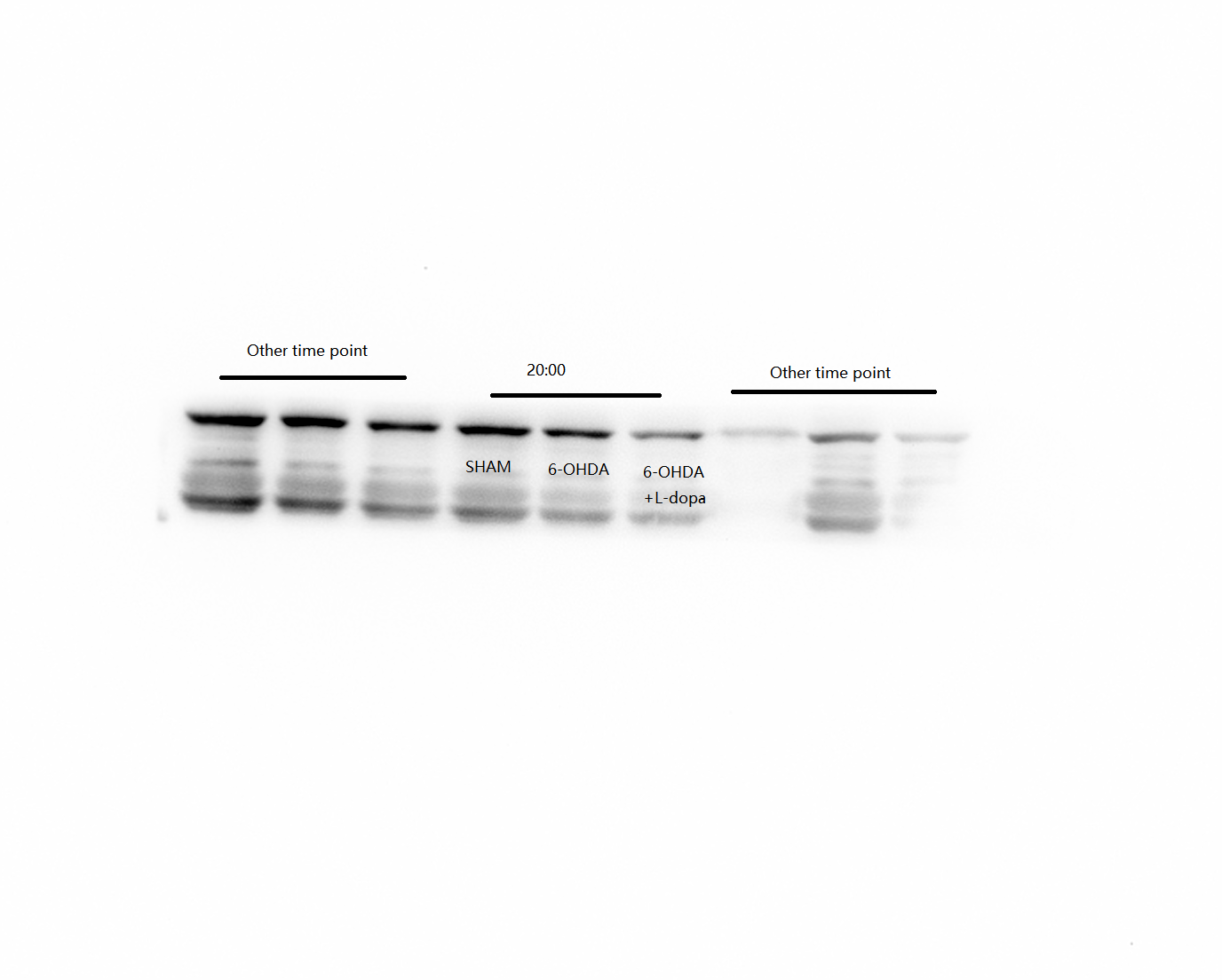

Supplement: Supplementary file 3 [file Data_Sheet_1.ZIP › full scan of the entire original gels/Figure4vs5/BMAL1/B/20H/BMAL1.tif]

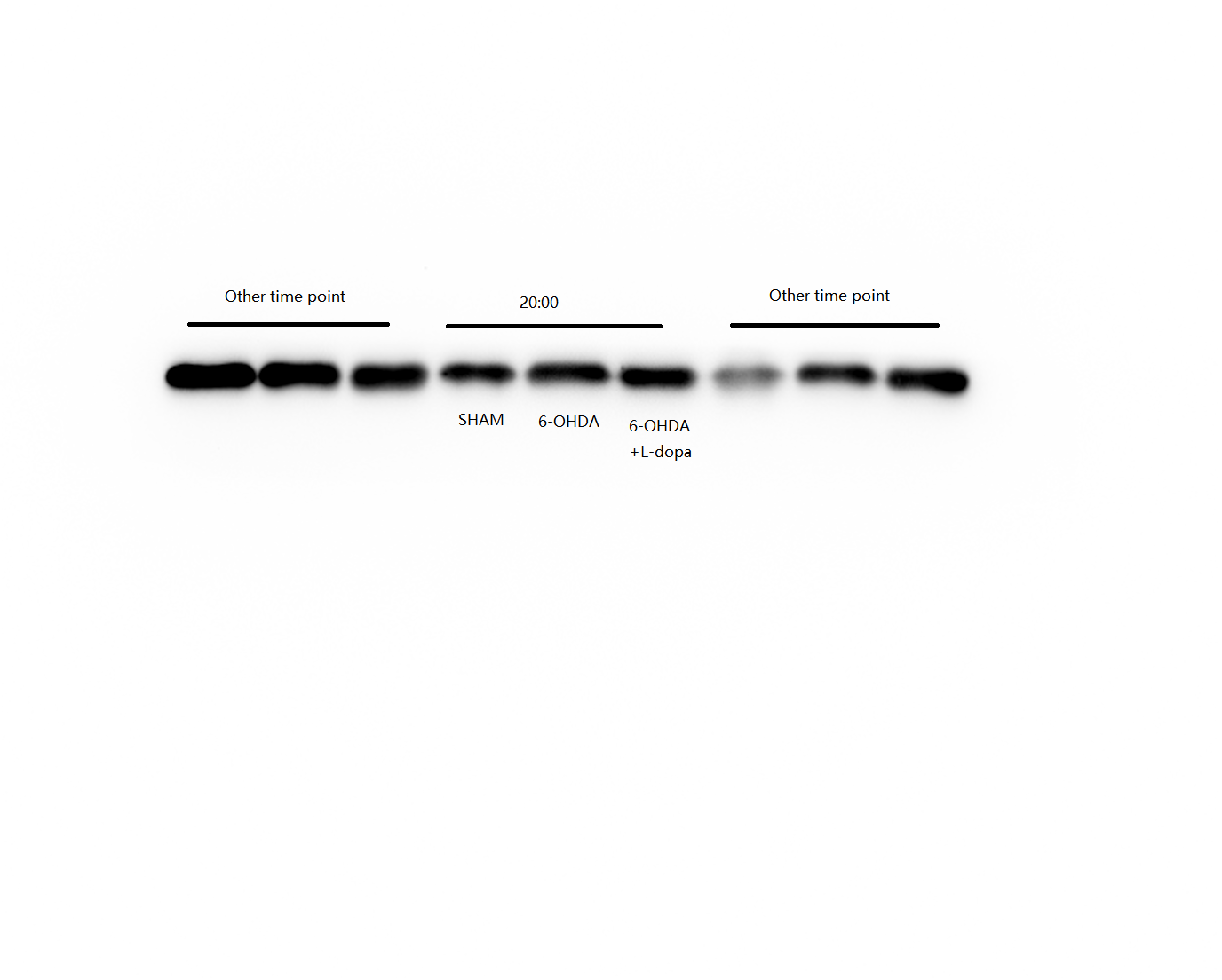

Supplement: Supplementary file 3 [file Data_Sheet_1.ZIP › full scan of the entire original gels/Figure4vs5/BMAL1/B/20H/GAPDH.tif]

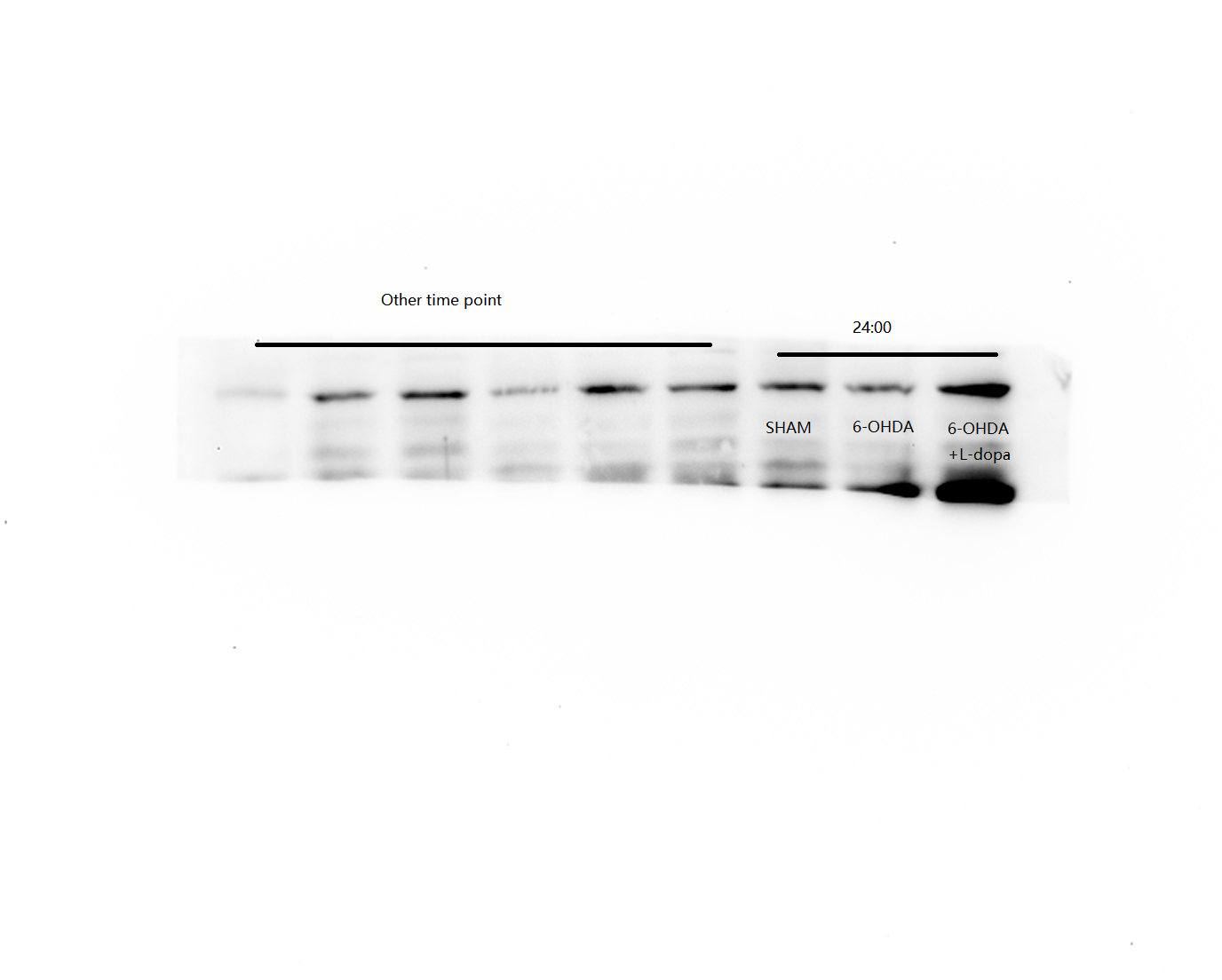

Supplement: Supplementary file 3 [file Data_Sheet_1.ZIP › full scan of the entire original gels/Figure4vs5/BMAL1/B/24H/BMAL1.tif]

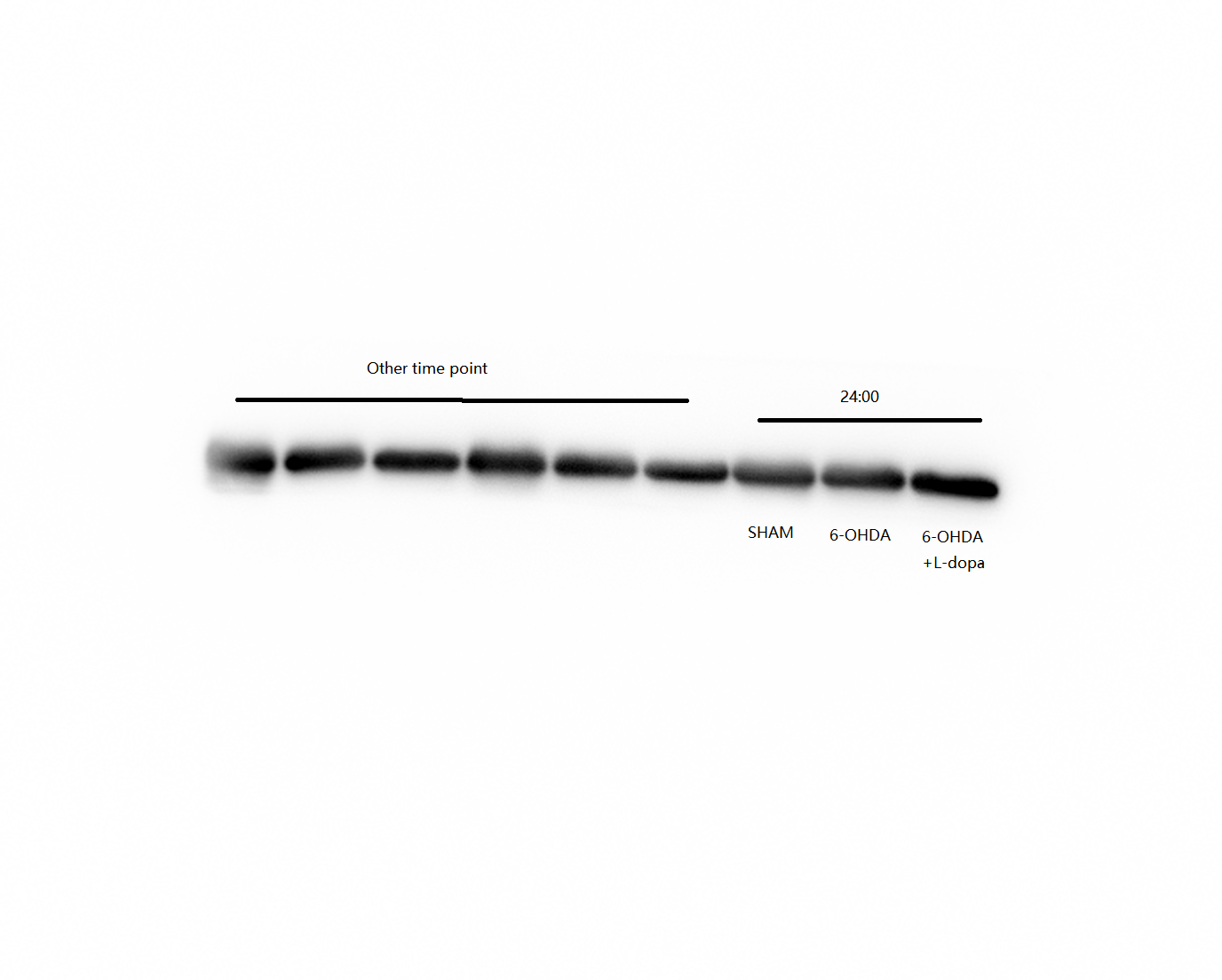

Supplement: Supplementary file 3 [file Data_Sheet_1.ZIP › full scan of the entire original gels/Figure4vs5/BMAL1/B/24H/GAPDH.tif]

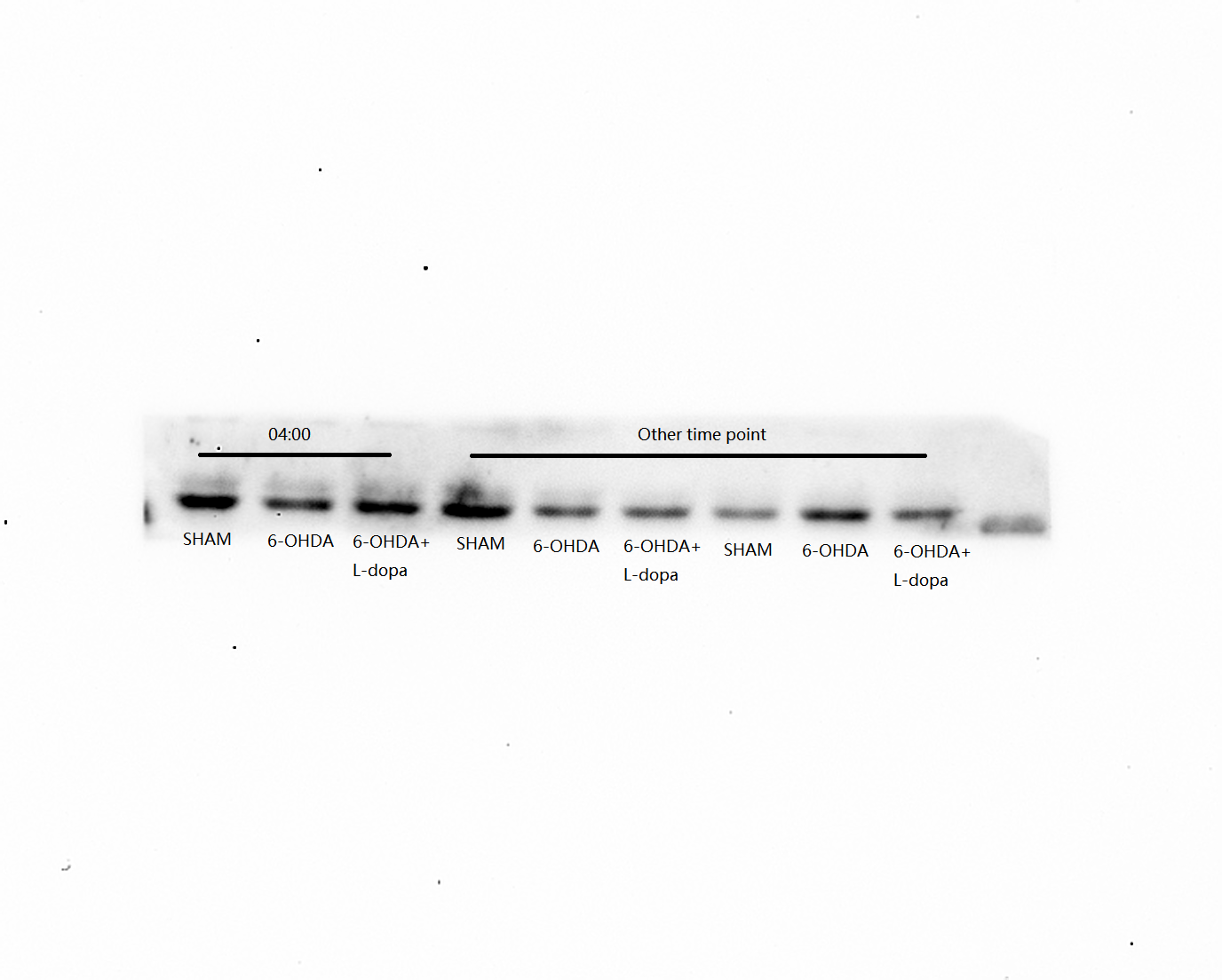

Supplement: Supplementary file 3 [file Data_Sheet_1.ZIP › full scan of the entire original gels/Figure4vs5/BMAL1/B/4H/bmal4-12-0913.tif]

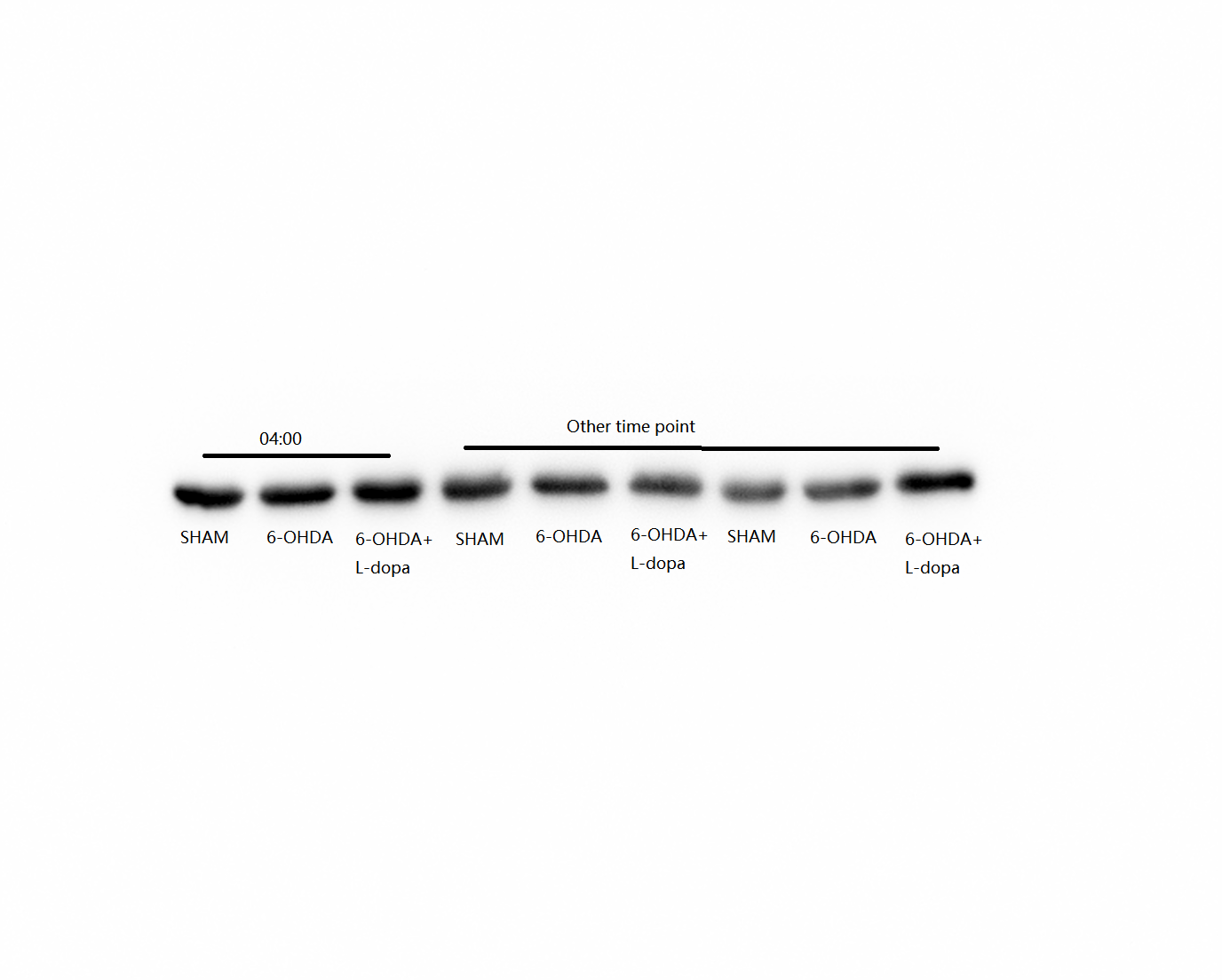

Supplement: Supplementary file 3 [file Data_Sheet_1.ZIP › full scan of the entire original gels/Figure4vs5/BMAL1/B/4H/gap4-16c-0913.tif]

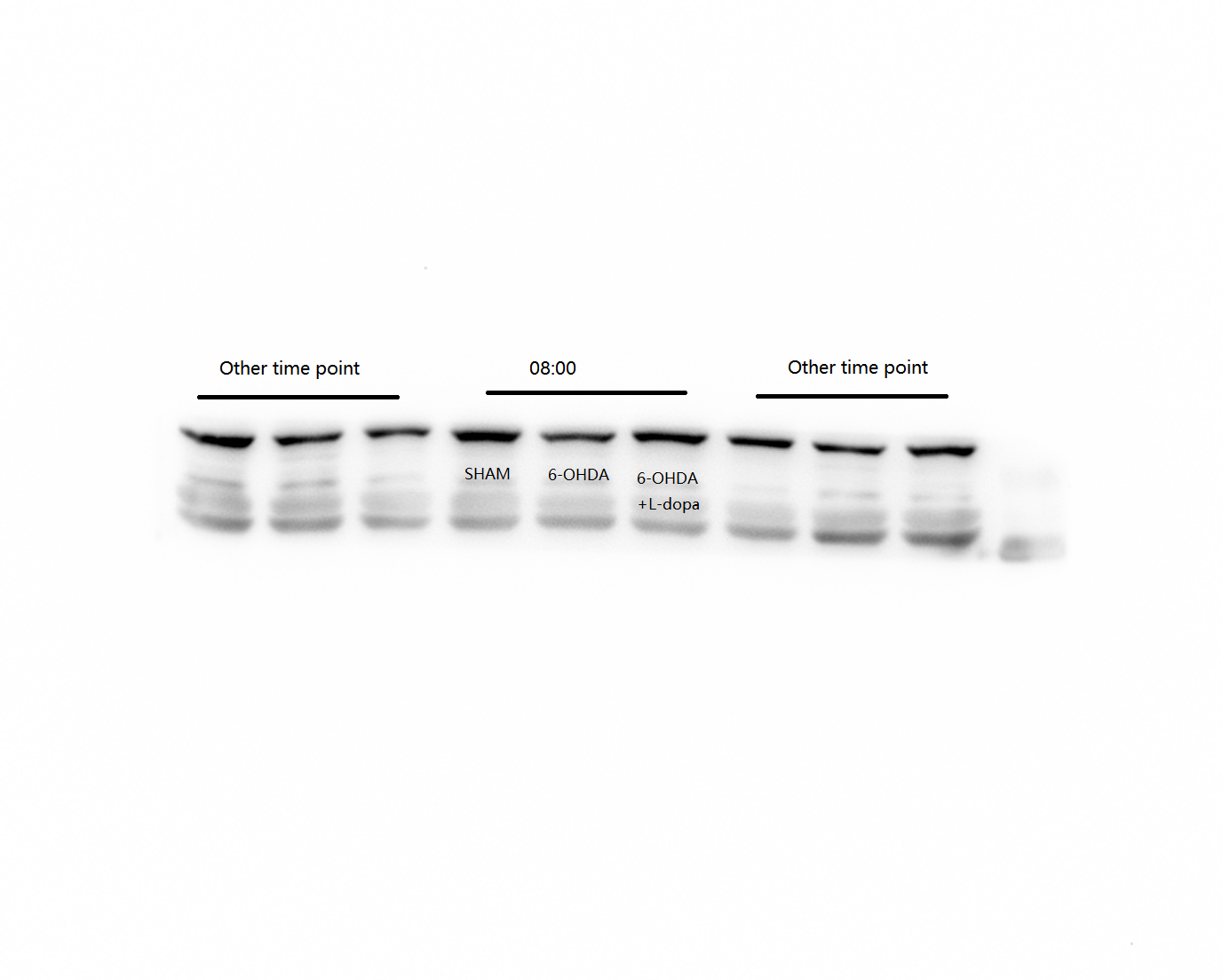

Supplement: Supplementary file 3 [file Data_Sheet_1.ZIP › full scan of the entire original gels/Figure4vs5/BMAL1/B/8H/BMAL1.tif]

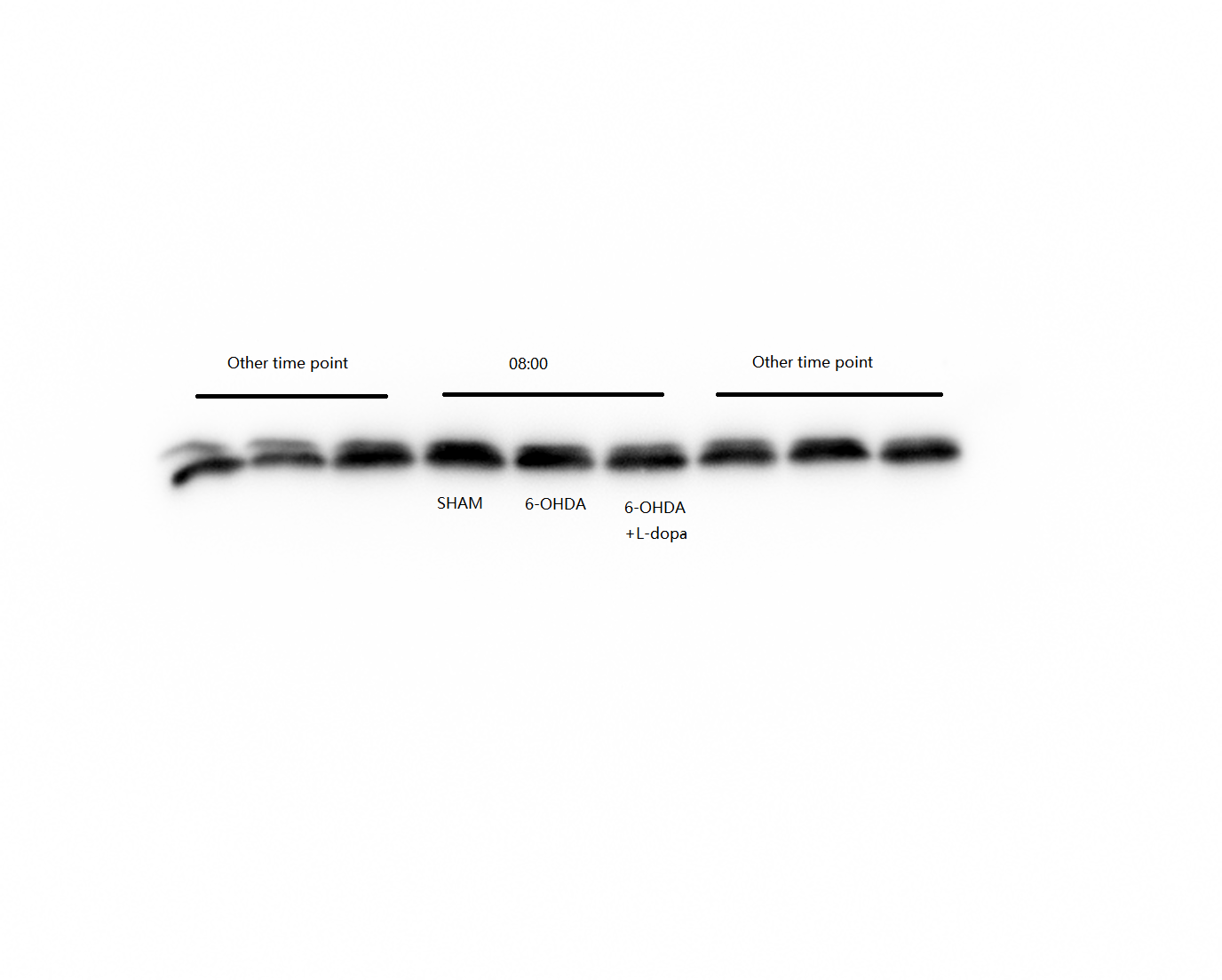

Supplement: Supplementary file 3 [file Data_Sheet_1.ZIP › full scan of the entire original gels/Figure4vs5/BMAL1/B/8H/GAPDH.tif]

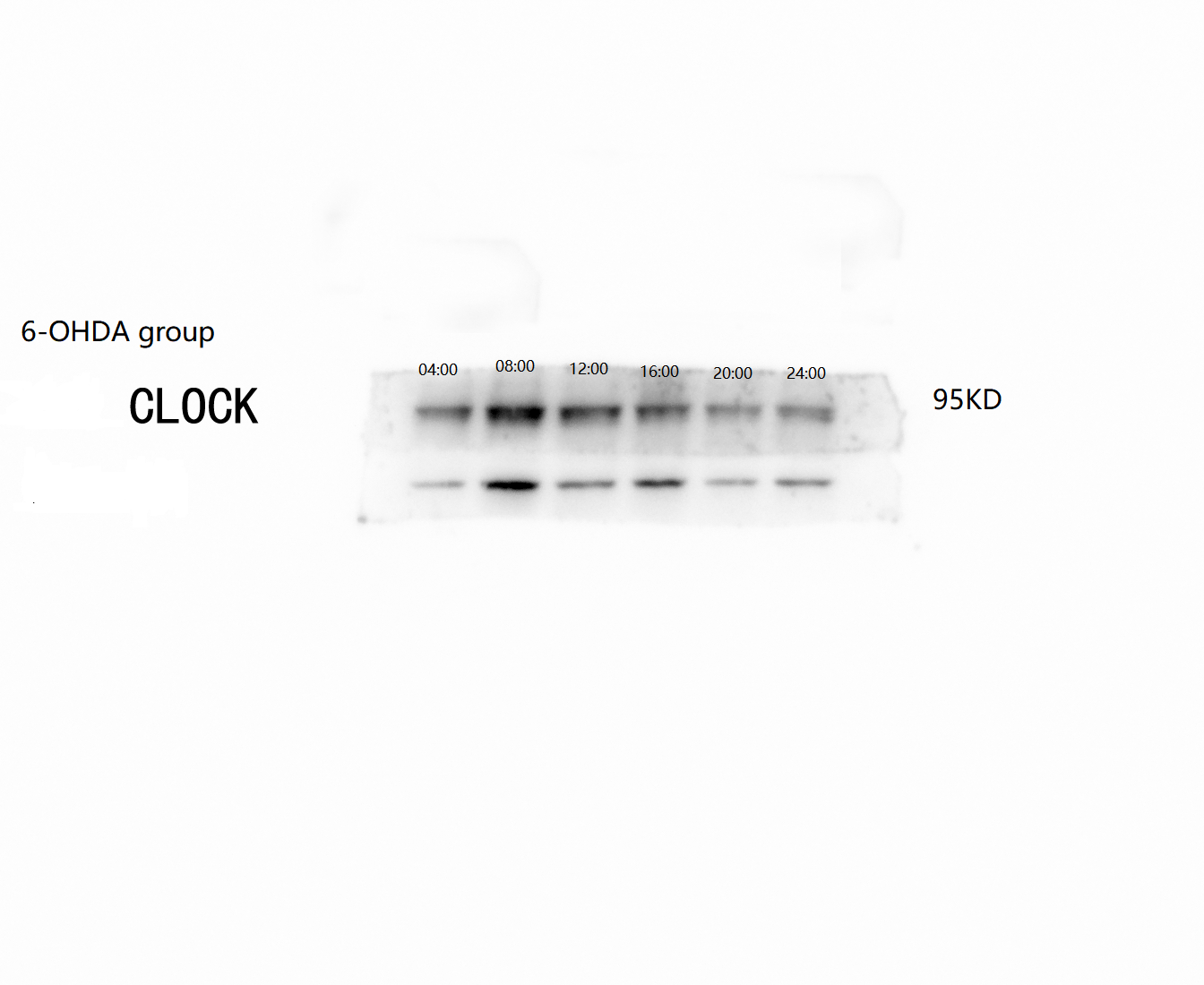

Supplement: Supplementary file 3 [file Data_Sheet_1.ZIP › full scan of the entire original gels/Figure4vs5/CLOCK/A/6-OHDA/CLOCK-6-OHDA.tif]

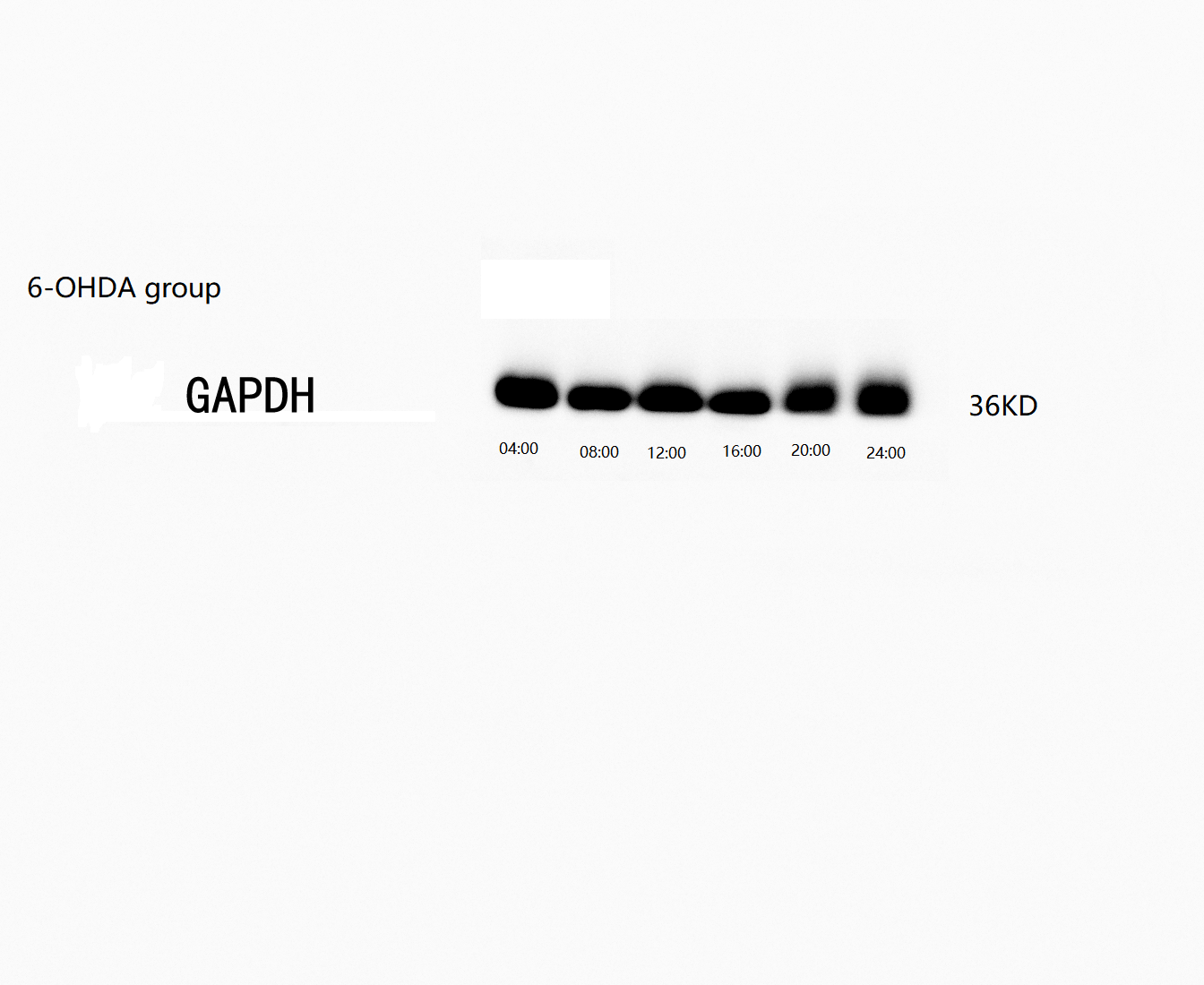

Supplement: Supplementary file 3 [file Data_Sheet_1.ZIP › full scan of the entire original gels/Figure4vs5/CLOCK/A/6-OHDA/GAPDH-6-OHDA.tif]

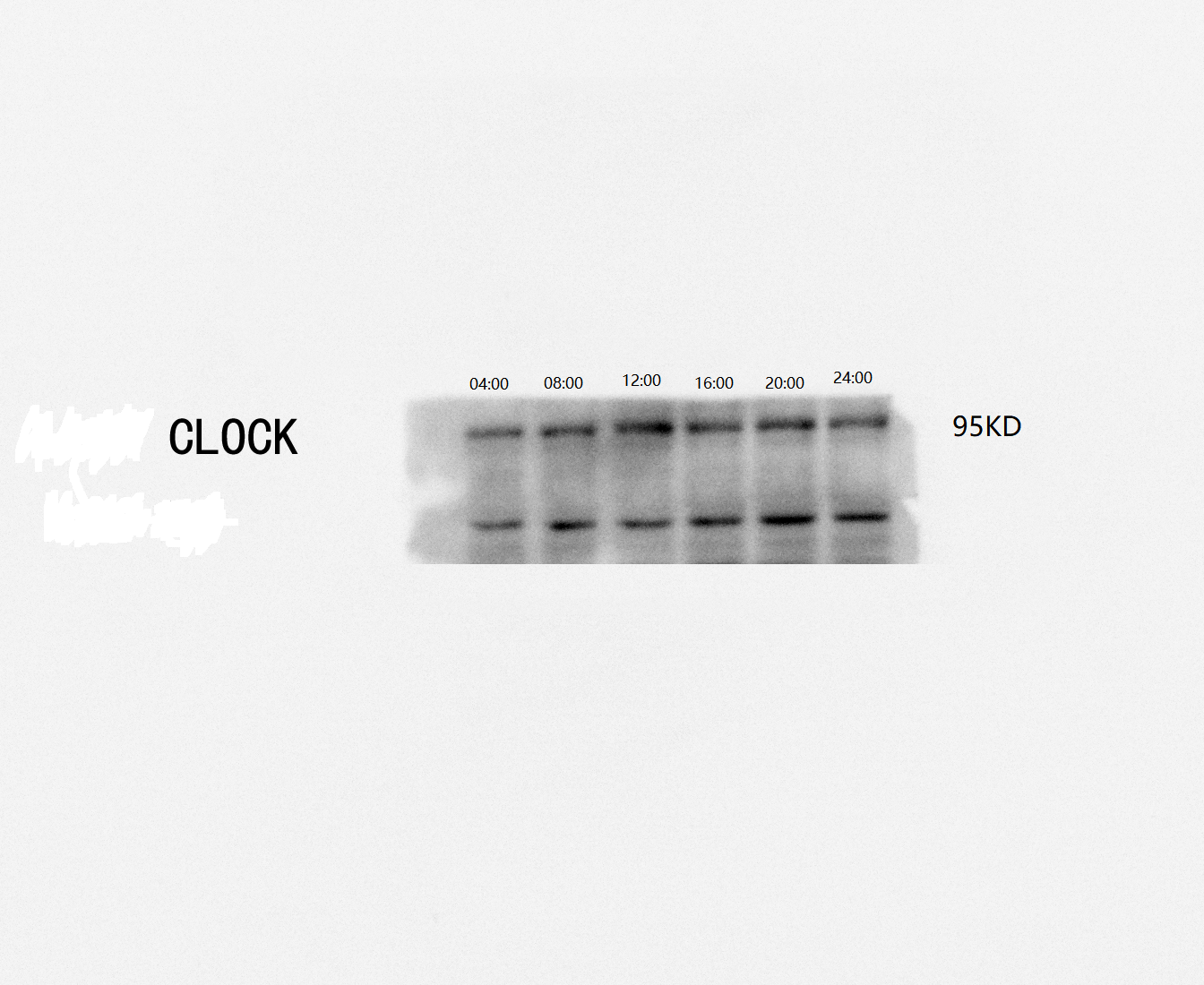

Supplement: Supplementary file 3 [file Data_Sheet_1.ZIP › full scan of the entire original gels/Figure4vs5/CLOCK/A/6-OHDA+L-dopa/6-OHDA+L-dopa-CLOCK.tif]

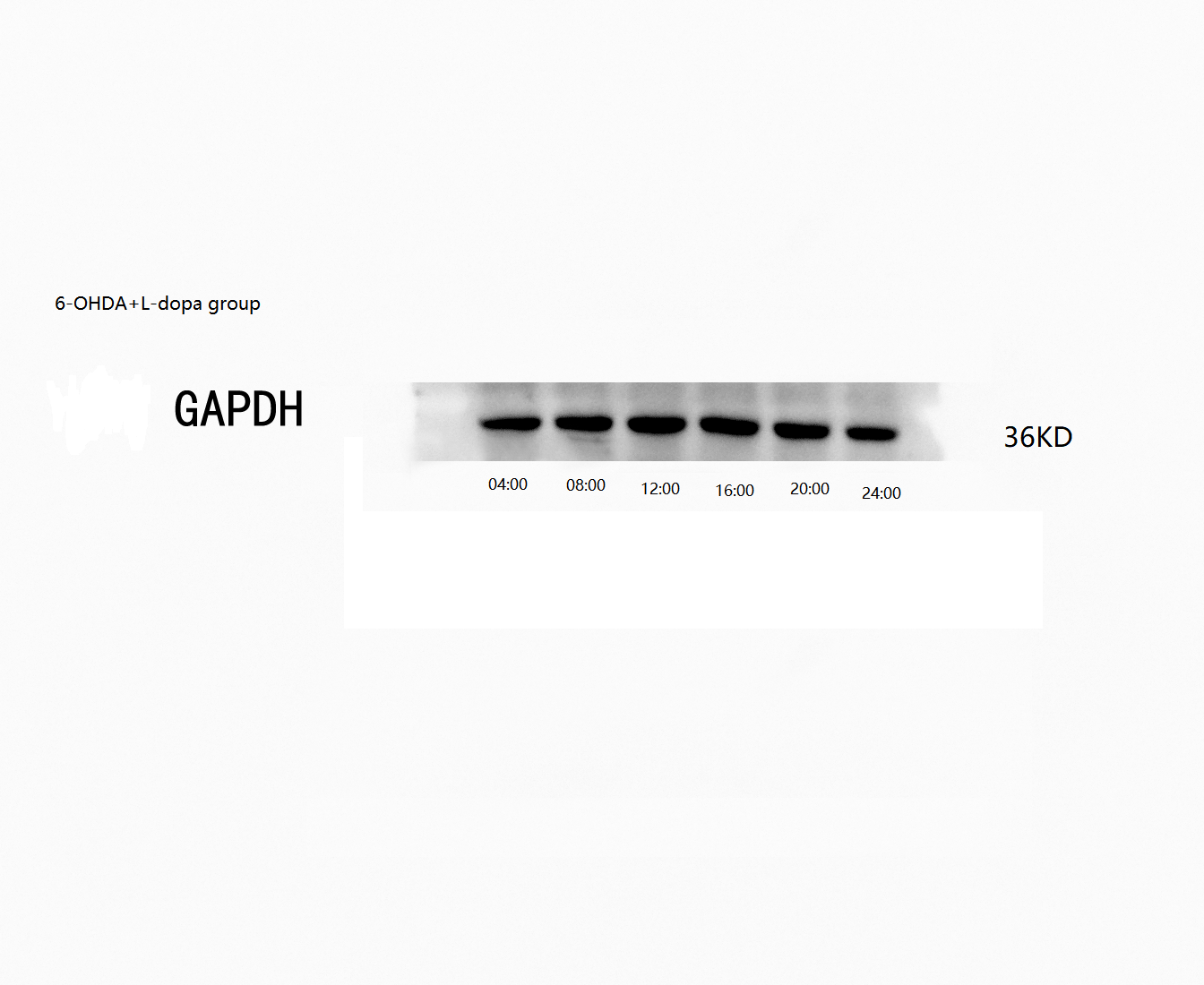

Supplement: Supplementary file 3 [file Data_Sheet_1.ZIP › full scan of the entire original gels/Figure4vs5/CLOCK/A/6-OHDA+L-dopa/6-OHDA+L-dopa-GAPDH.tif]

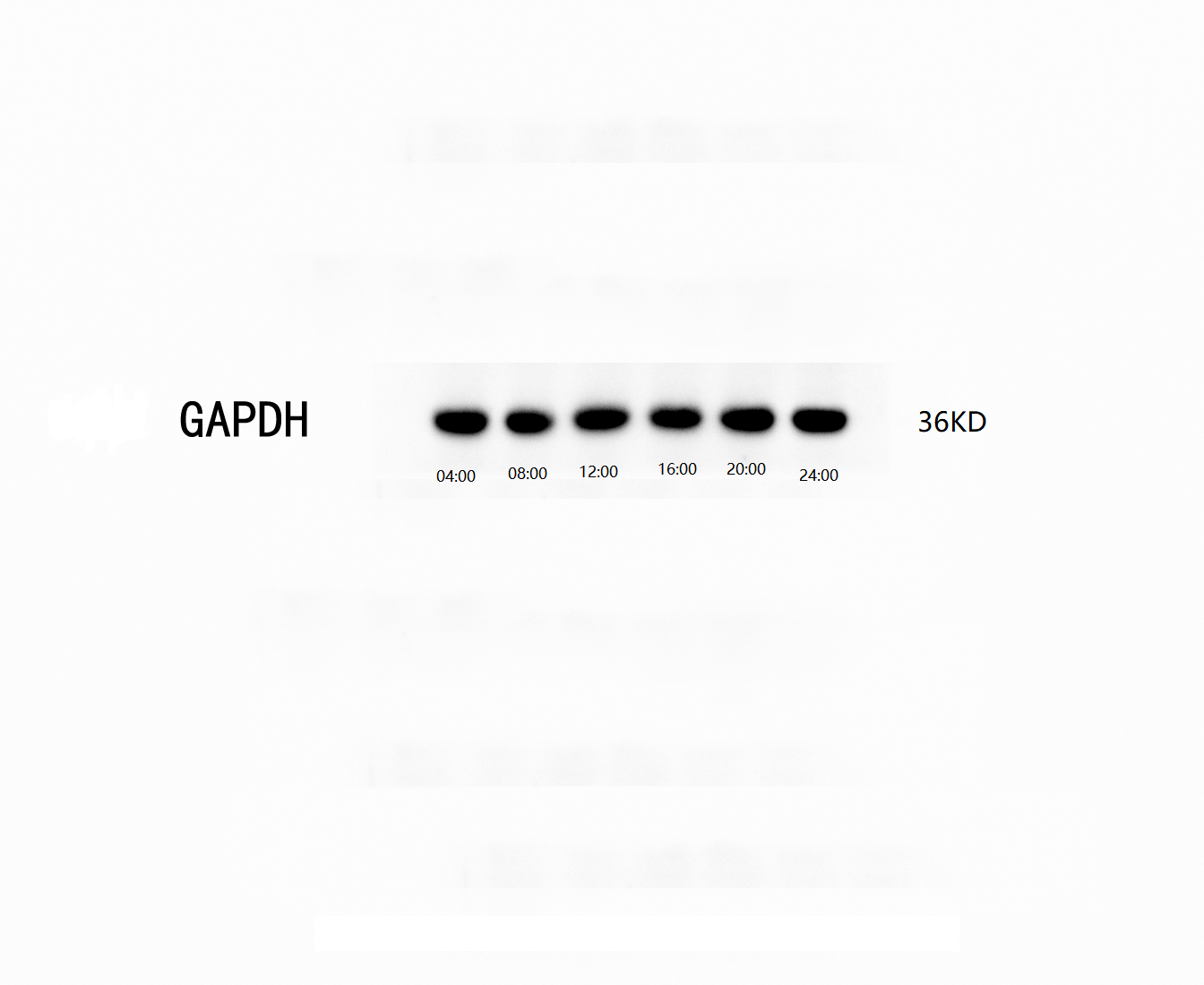

Supplement: Supplementary file 3 [file Data_Sheet_1.ZIP › full scan of the entire original gels/Figure4vs5/CLOCK/A/SHAM/GAPDH-SHAM.tif]

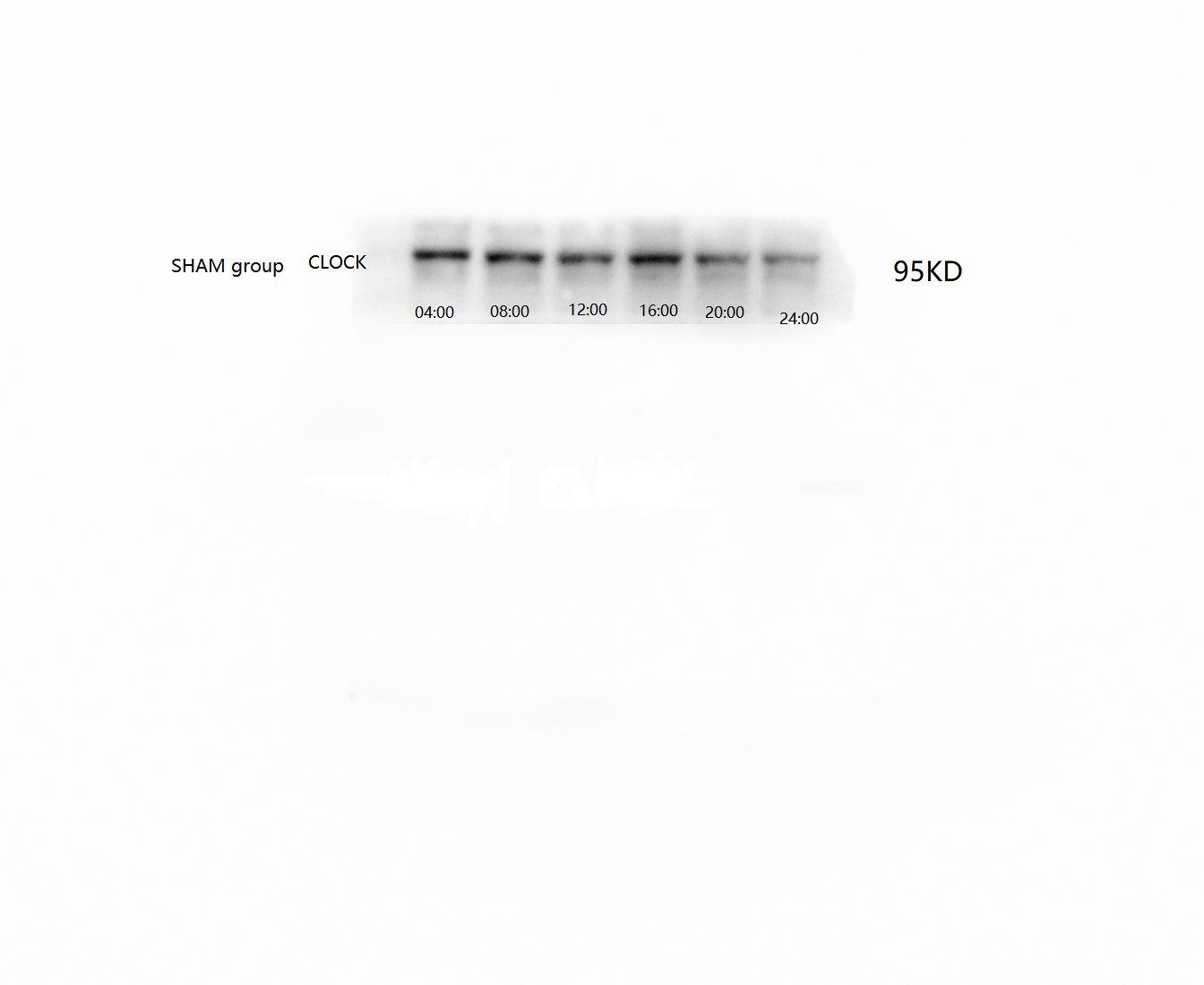

Supplement: Supplementary file 3 [file Data_Sheet_1.ZIP › full scan of the entire original gels/Figure4vs5/CLOCK/A/SHAM/sham-clock.tif]

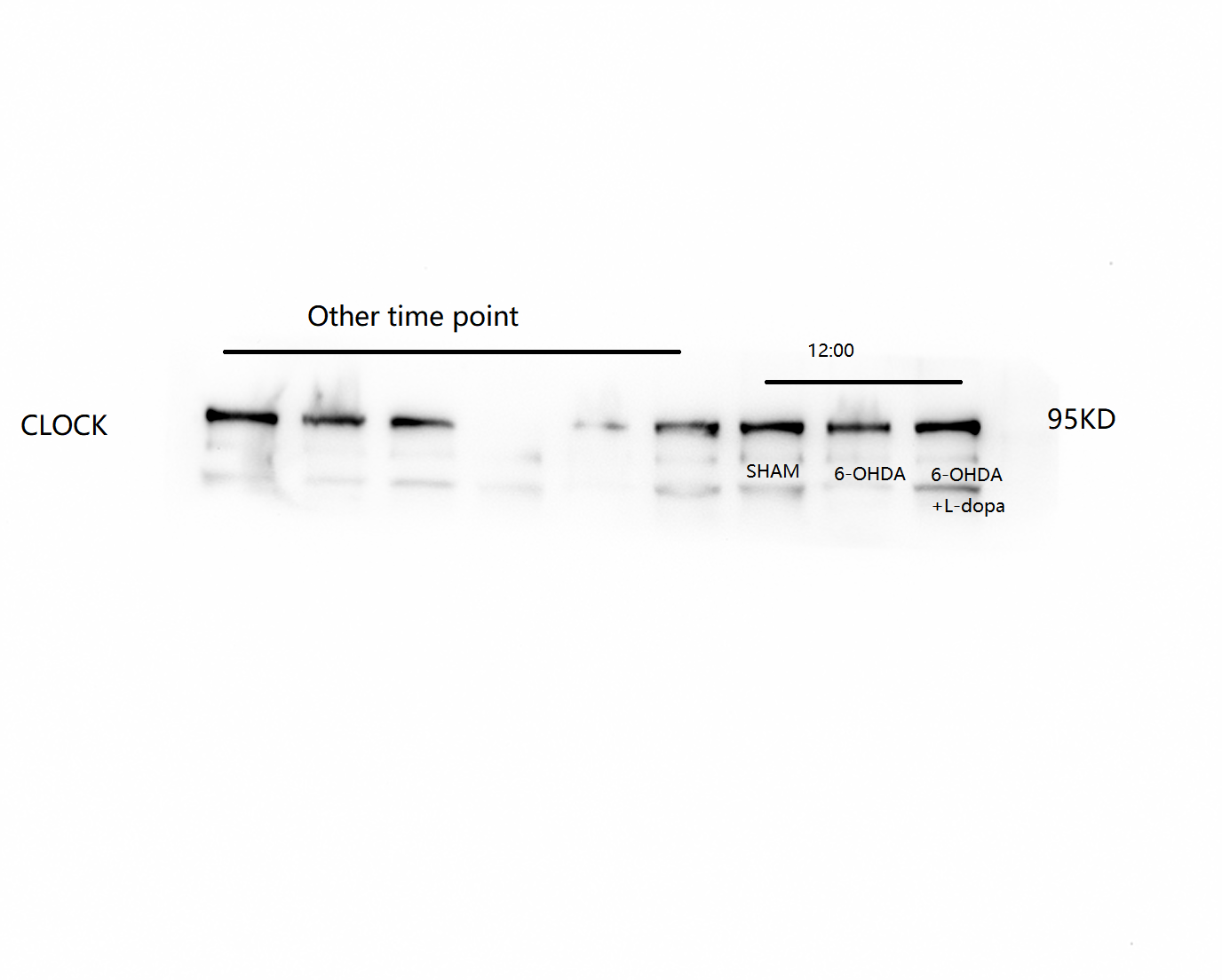

Supplement: Supplementary file 3 [file Data_Sheet_1.ZIP › full scan of the entire original gels/Figure4vs5/CLOCK/B/12H/CLOCK.tif]

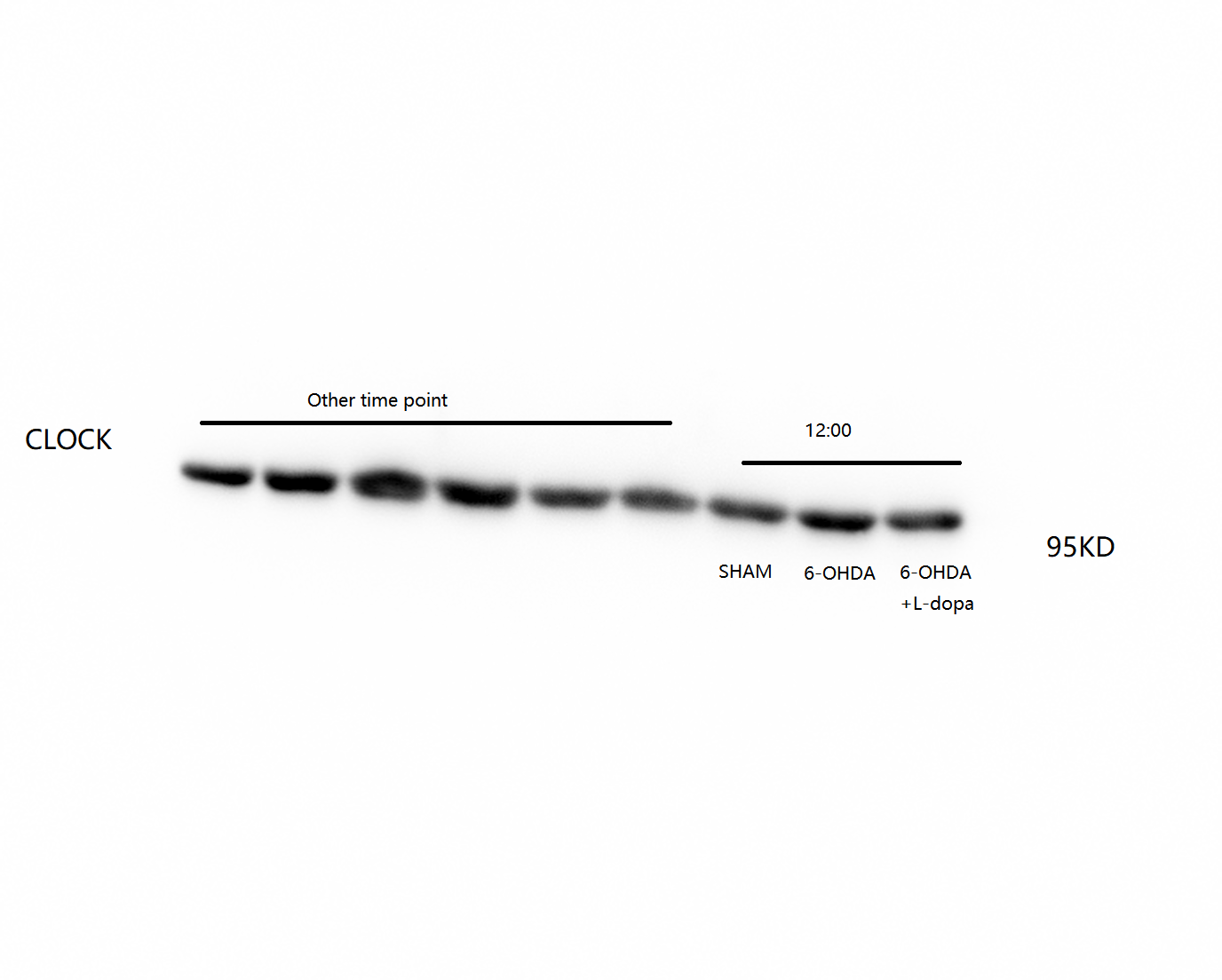

Supplement: Supplementary file 3 [file Data_Sheet_1.ZIP › full scan of the entire original gels/Figure4vs5/CLOCK/B/12H/GAPDH.tif]

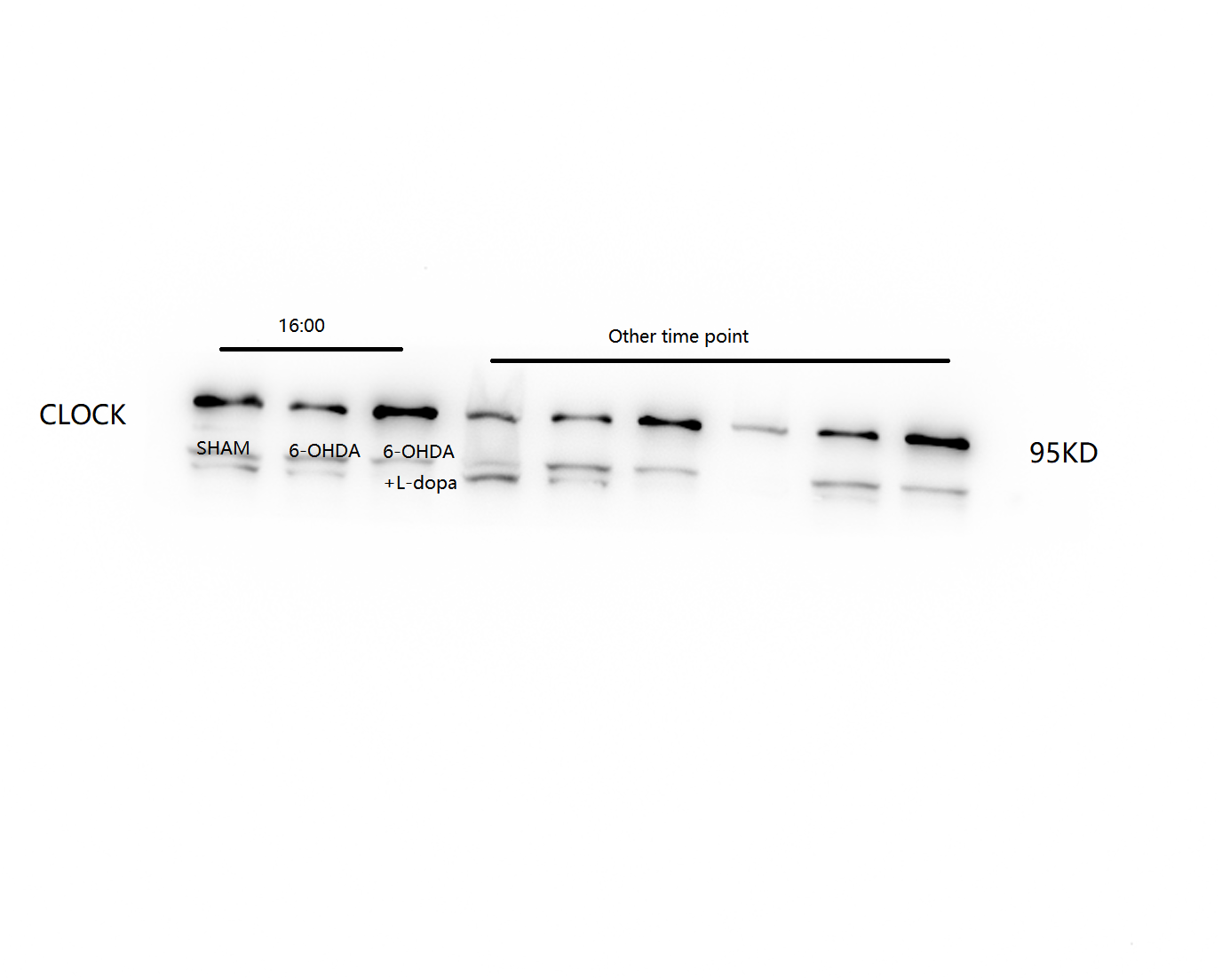

Supplement: Supplementary file 3 [file Data_Sheet_1.ZIP › full scan of the entire original gels/Figure4vs5/CLOCK/B/16H/CLOCK.tif]

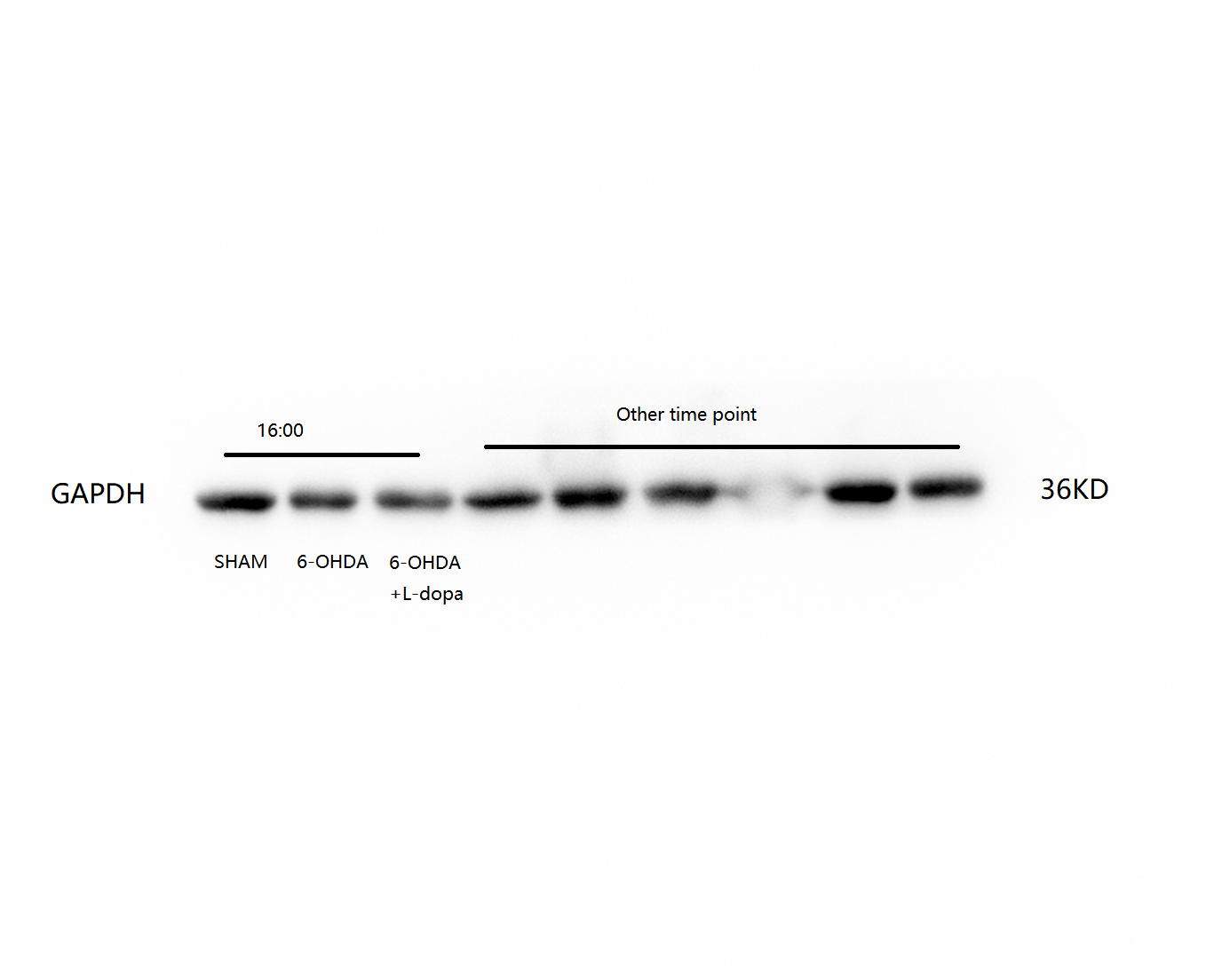

Supplement: Supplementary file 3 [file Data_Sheet_1.ZIP › full scan of the entire original gels/Figure4vs5/CLOCK/B/16H/GAPDH.tif]

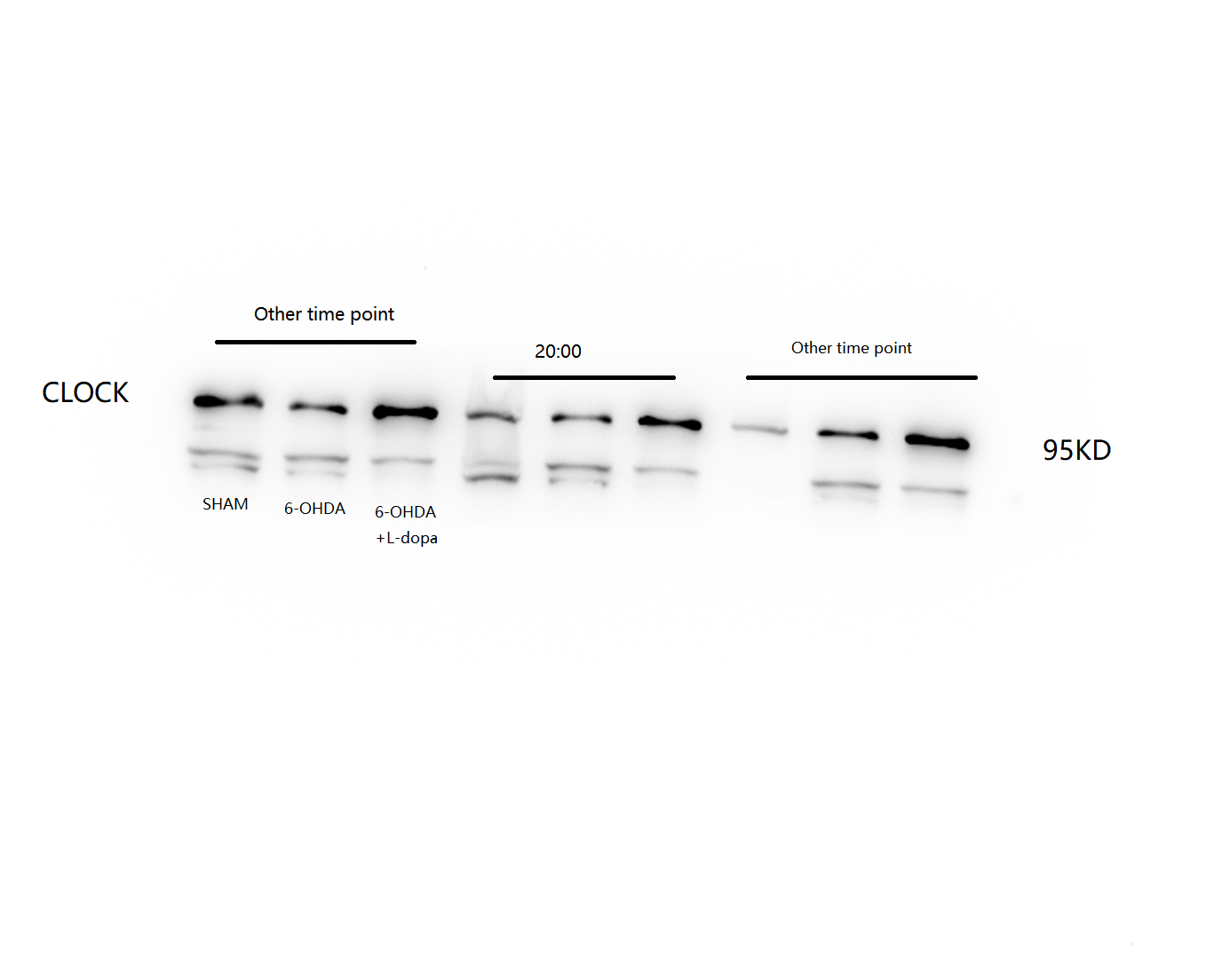

Supplement: Supplementary file 3 [file Data_Sheet_1.ZIP › full scan of the entire original gels/Figure4vs5/CLOCK/B/20H/CLOCK.tif]

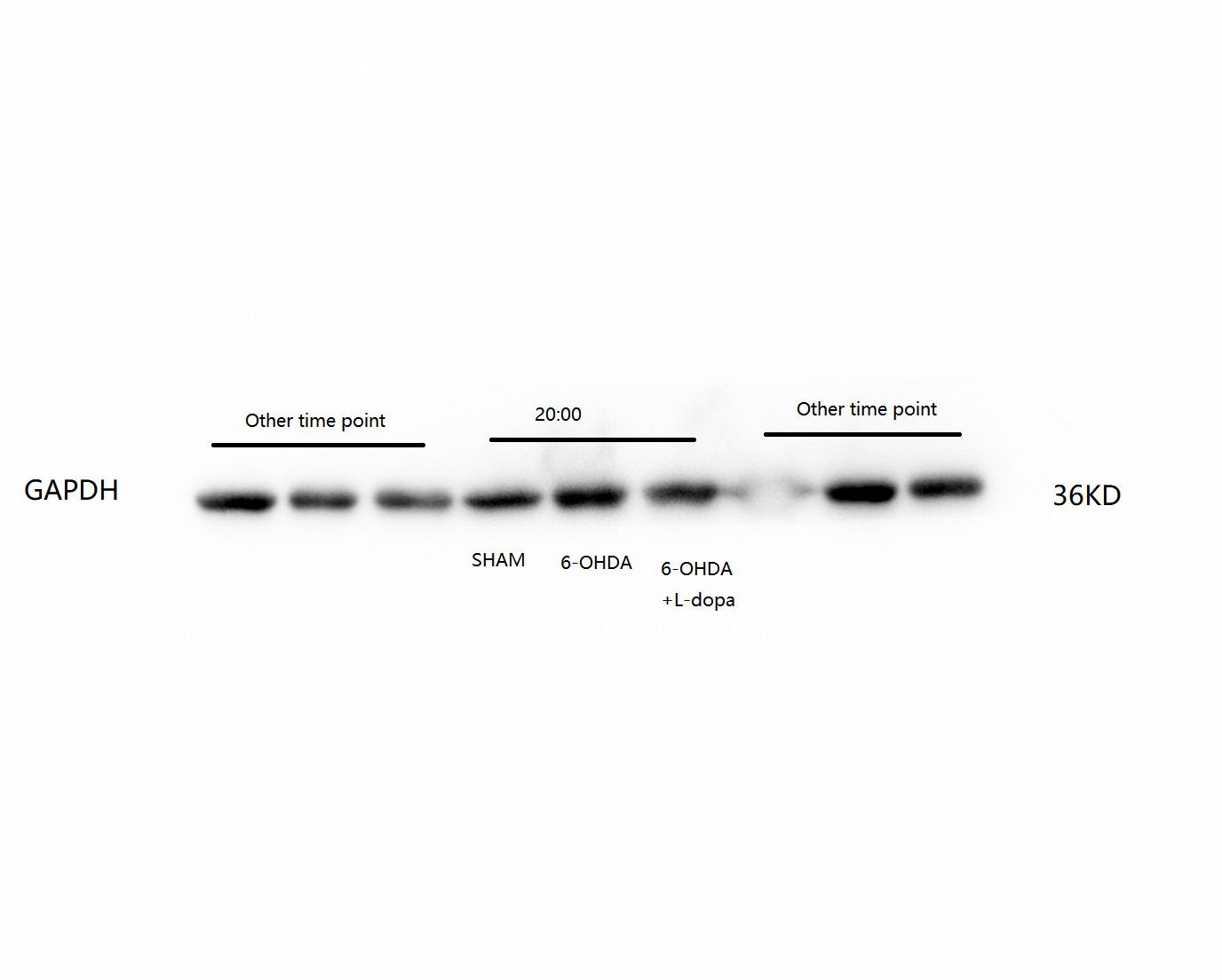

Supplement: Supplementary file 3 [file Data_Sheet_1.ZIP › full scan of the entire original gels/Figure4vs5/CLOCK/B/20H/GAPDH.tif]

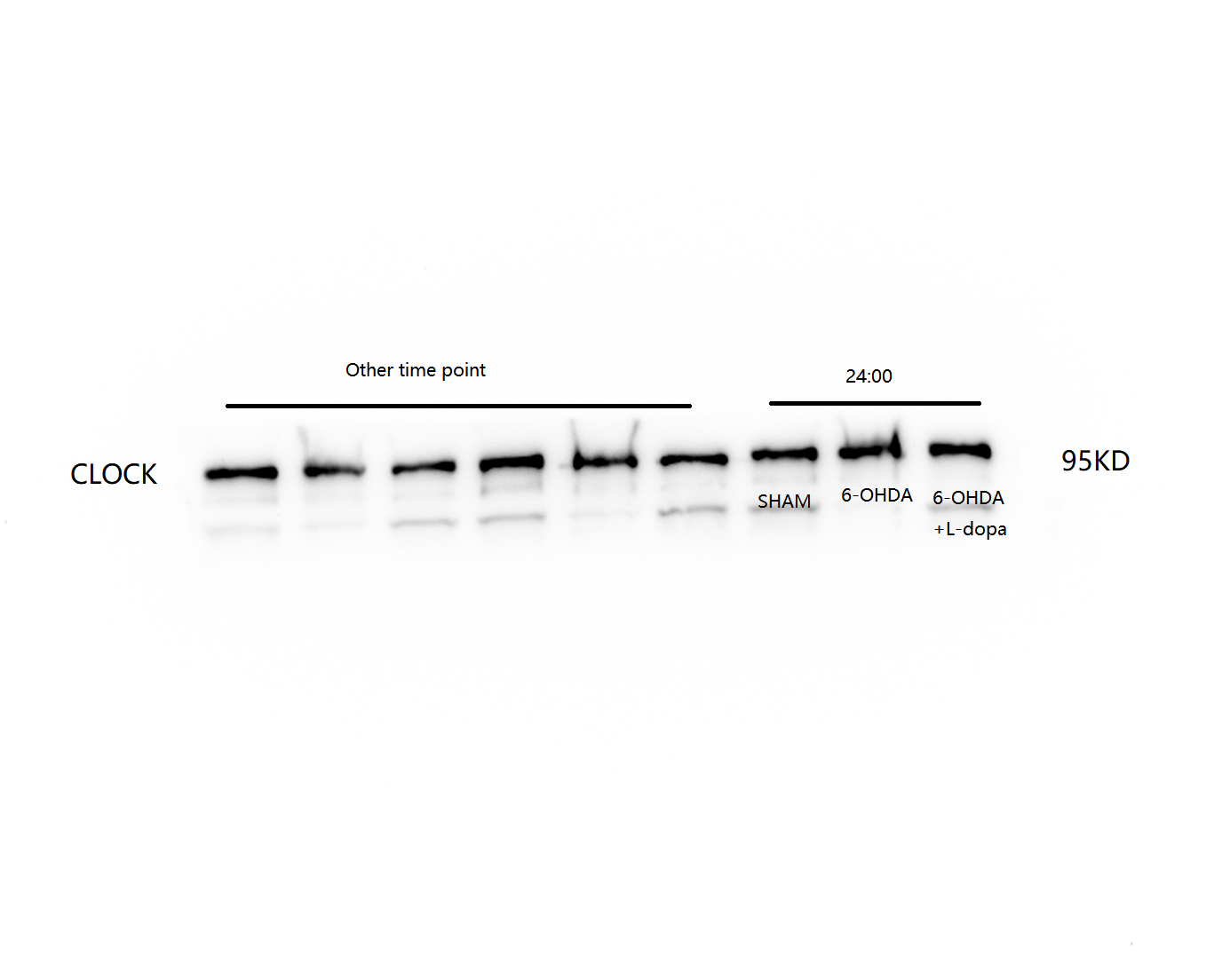

Supplement: Supplementary file 3 [file Data_Sheet_1.ZIP › full scan of the entire original gels/Figure4vs5/CLOCK/B/24H/CLOCK.tif]

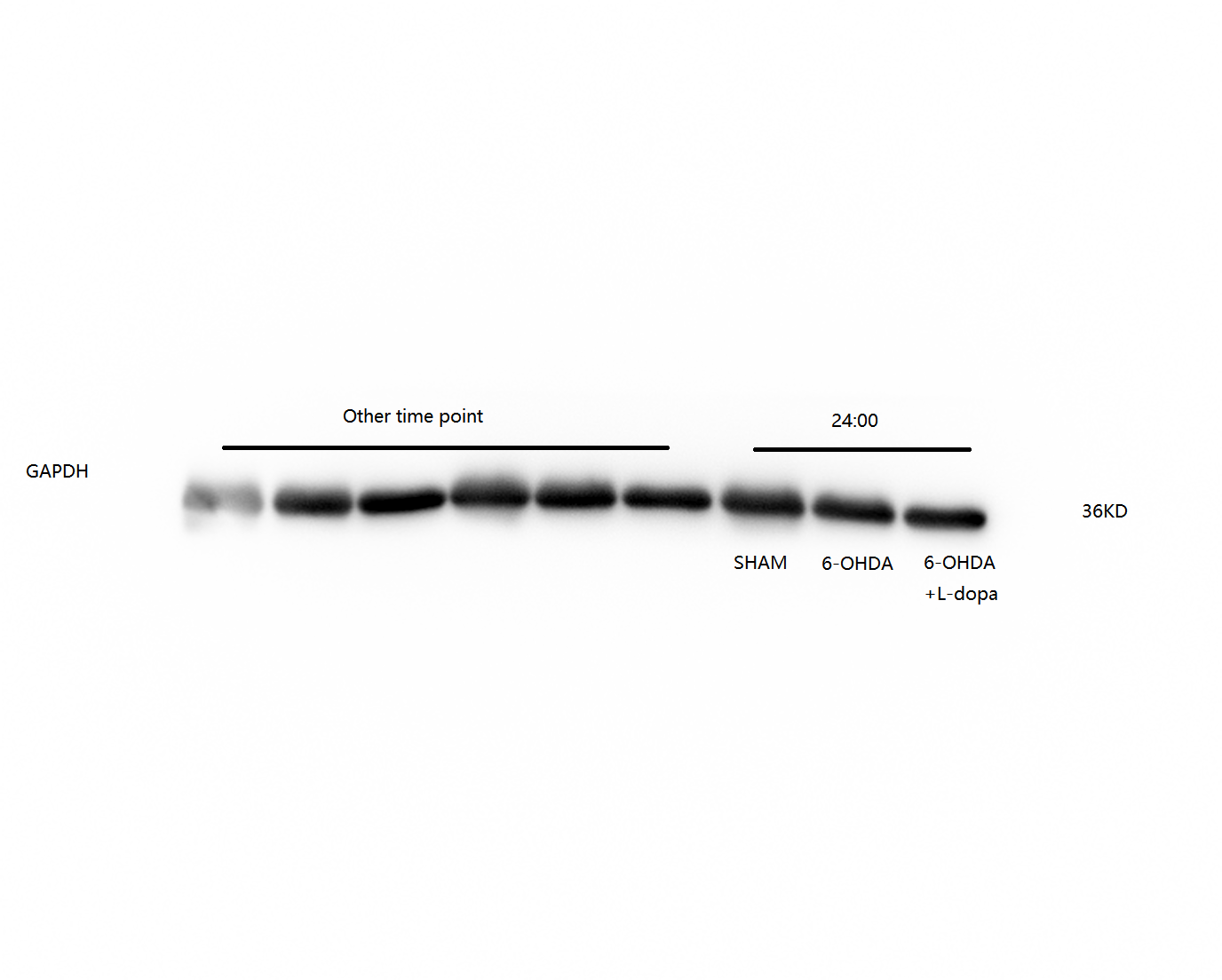

Supplement: Supplementary file 3 [file Data_Sheet_1.ZIP › full scan of the entire original gels/Figure4vs5/CLOCK/B/24H/GAPDH.tif]

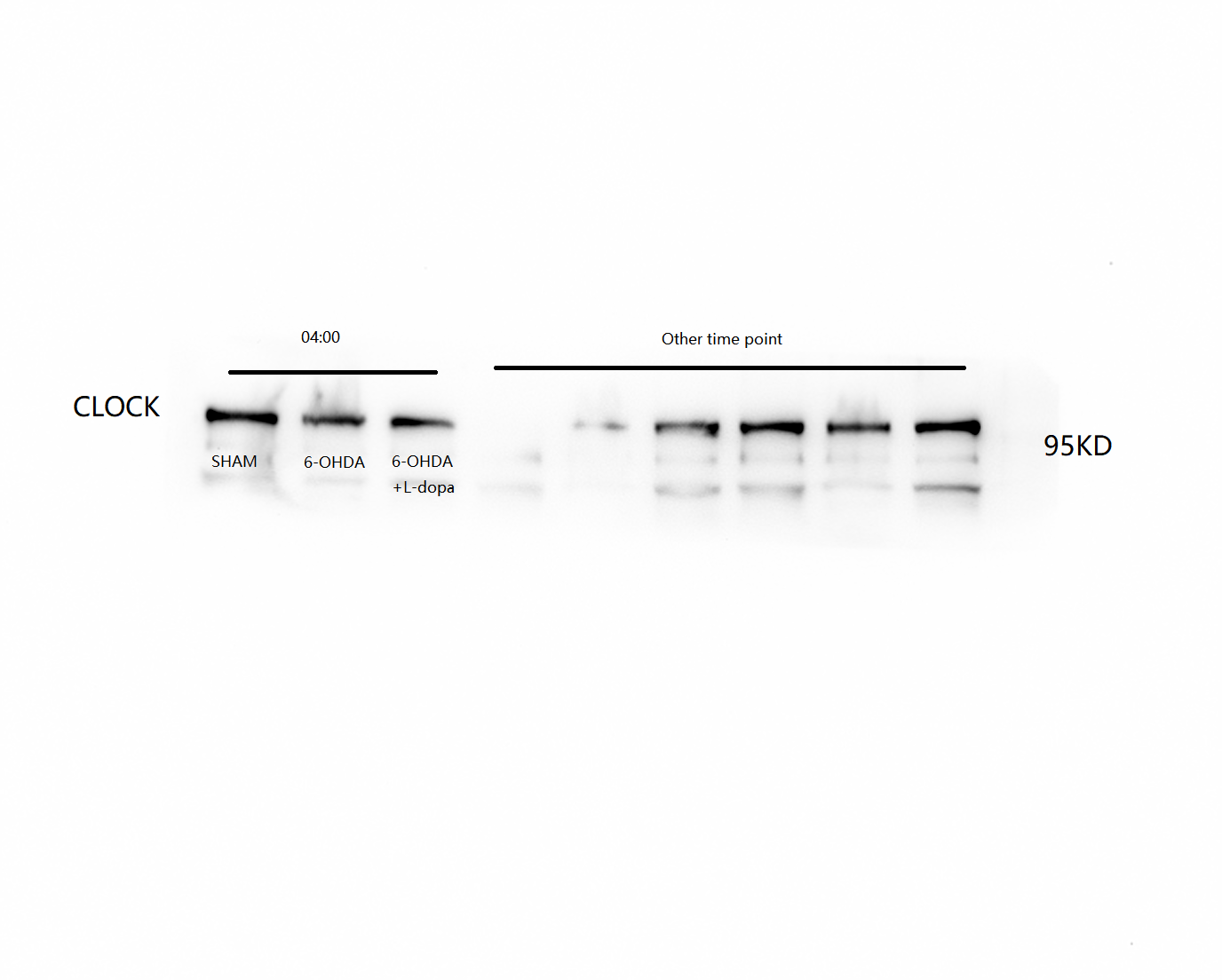

Supplement: Supplementary file 3 [file Data_Sheet_1.ZIP › full scan of the entire original gels/Figure4vs5/CLOCK/B/4H/CLOCK.tif]

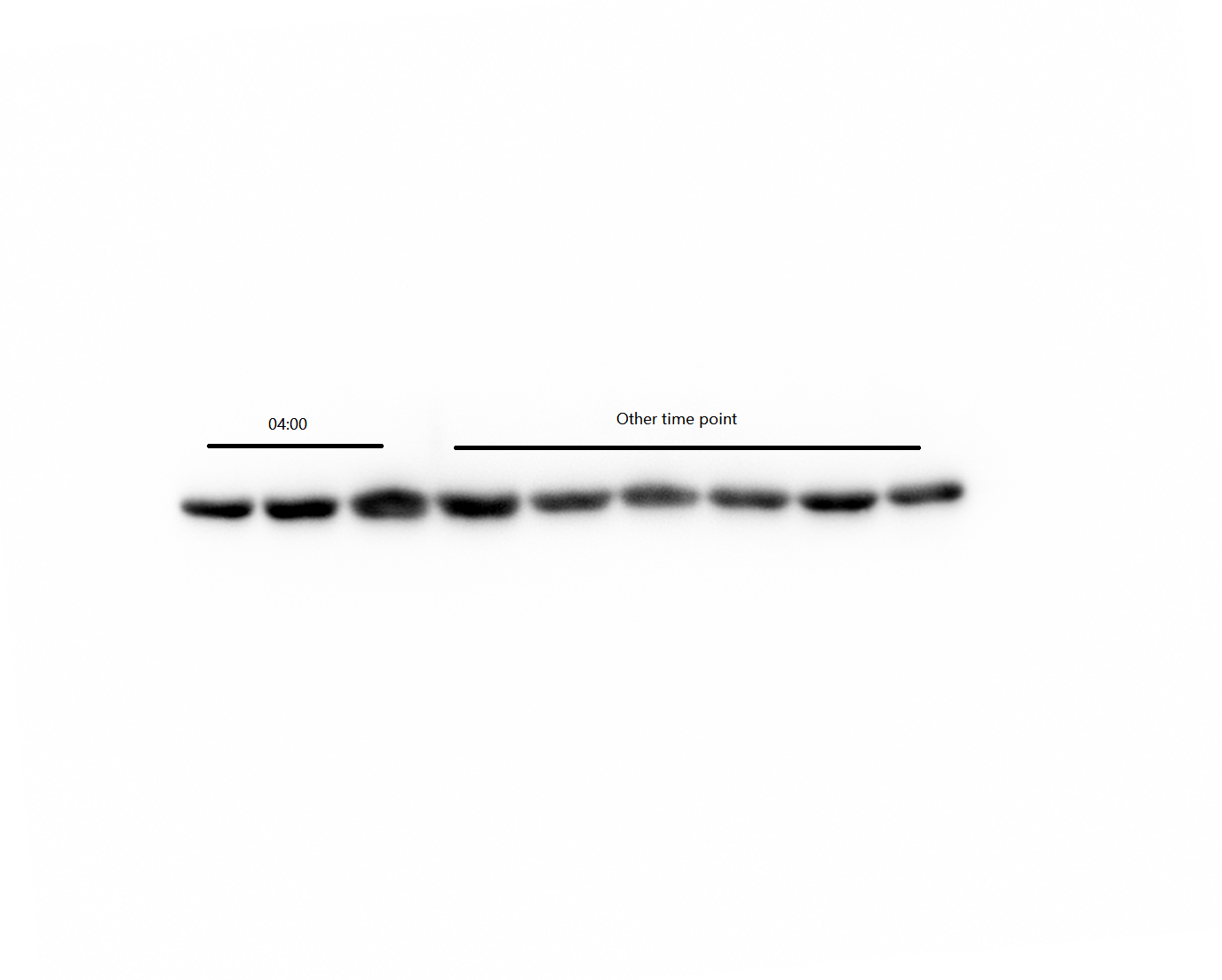

Supplement: Supplementary file 3 [file Data_Sheet_1.ZIP › full scan of the entire original gels/Figure4vs5/CLOCK/B/4H/GAPDH.tif]

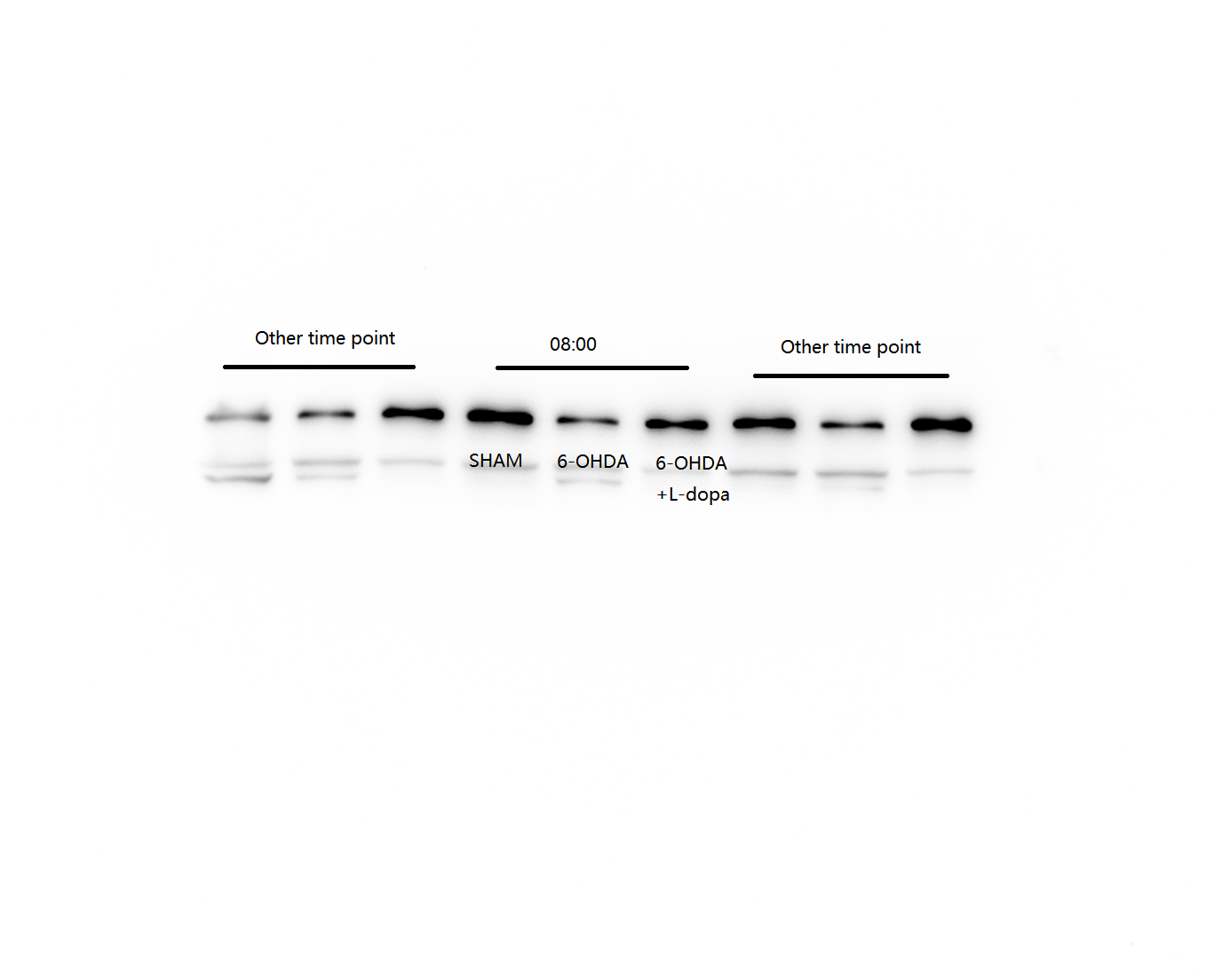

Supplement: Supplementary file 3 [file Data_Sheet_1.ZIP › full scan of the entire original gels/Figure4vs5/CLOCK/B/8H/CLOCK.tif]

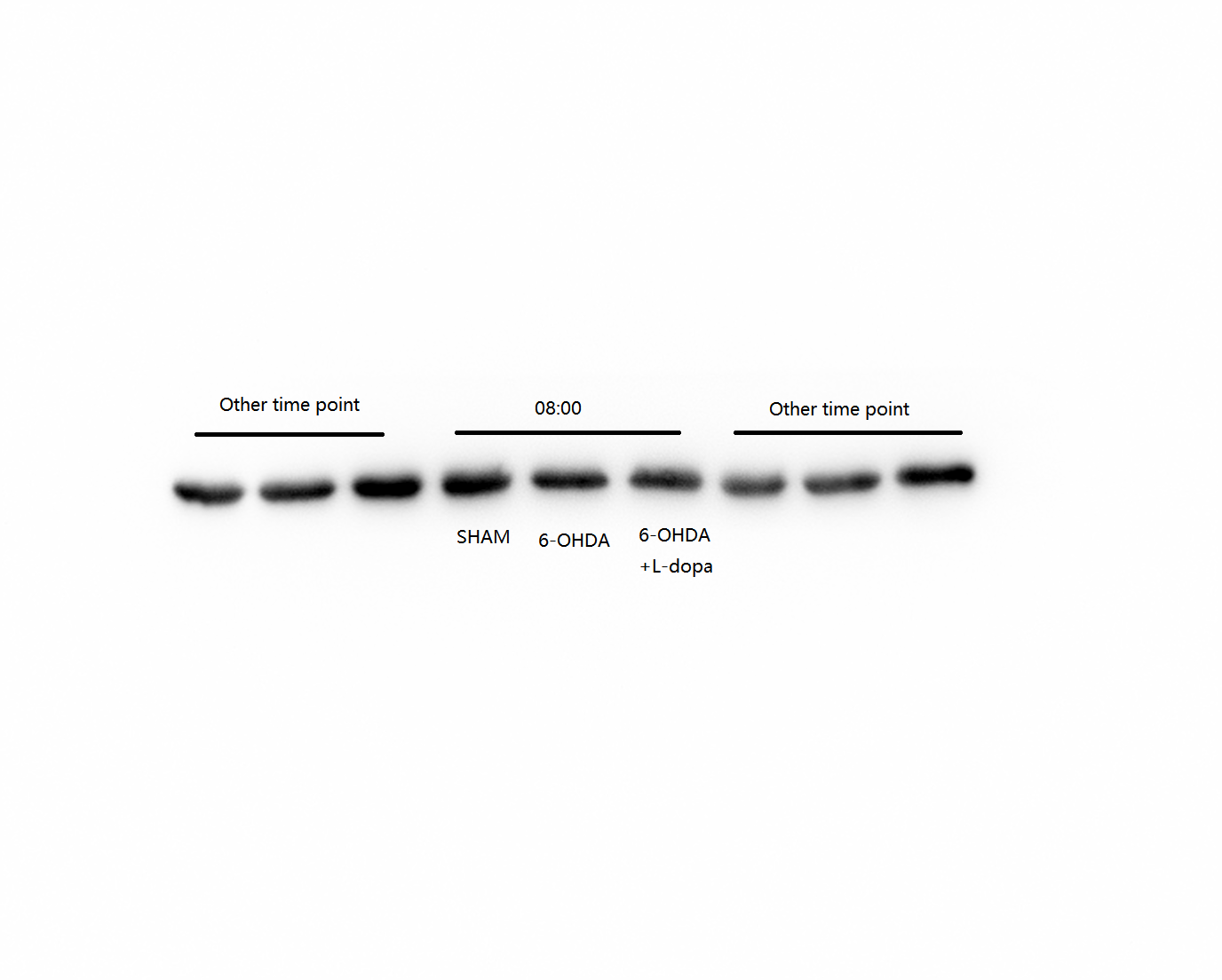

Supplement: Supplementary file 3 [file Data_Sheet_1.ZIP › full scan of the entire original gels/Figure4vs5/CLOCK/B/8H/GAPDH.tif]

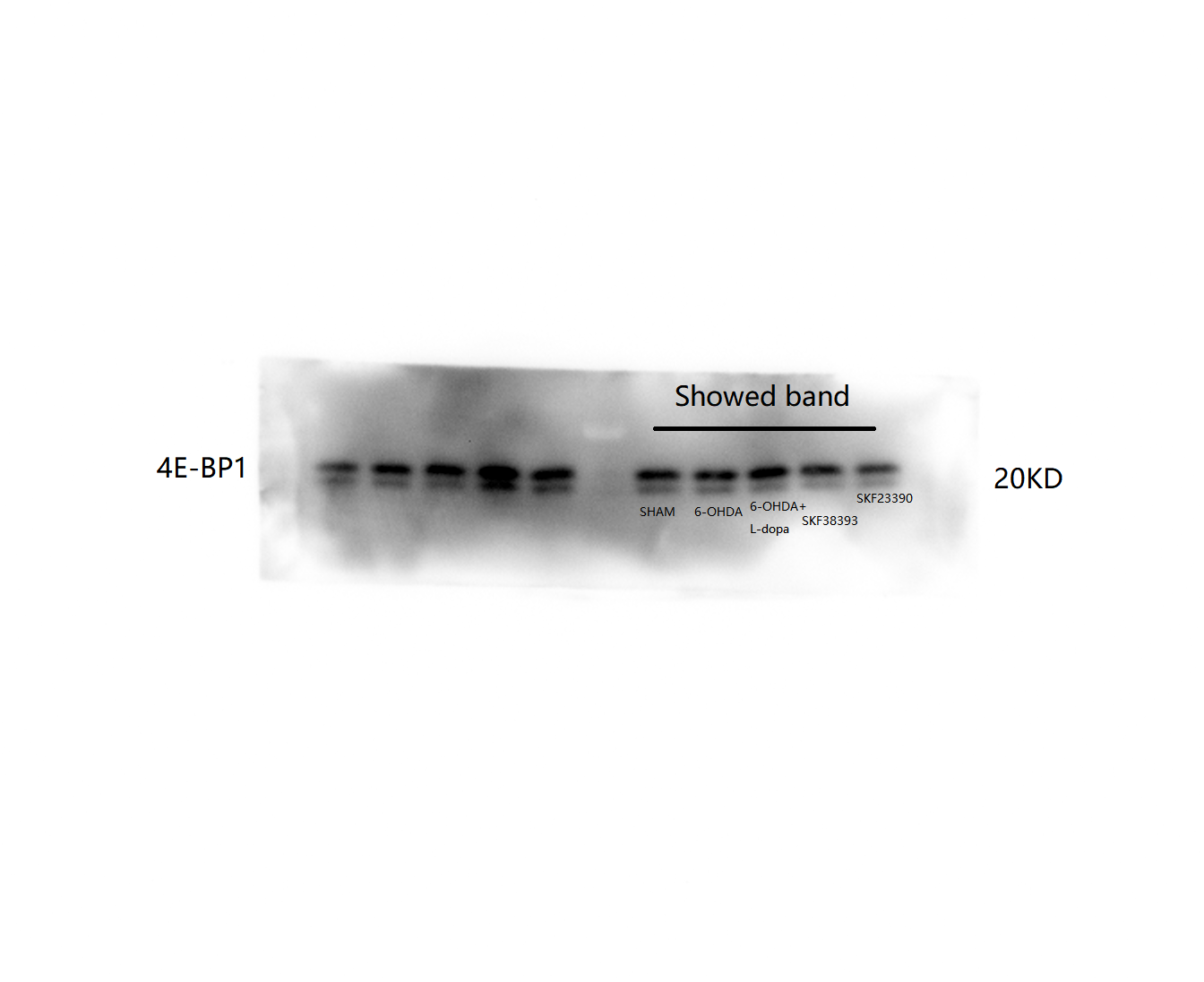

Supplement: Supplementary file 3 [file Data_Sheet_1.ZIP › full scan of the entire original gels/Figure6/4E-BP1/4EBP1.Tif]

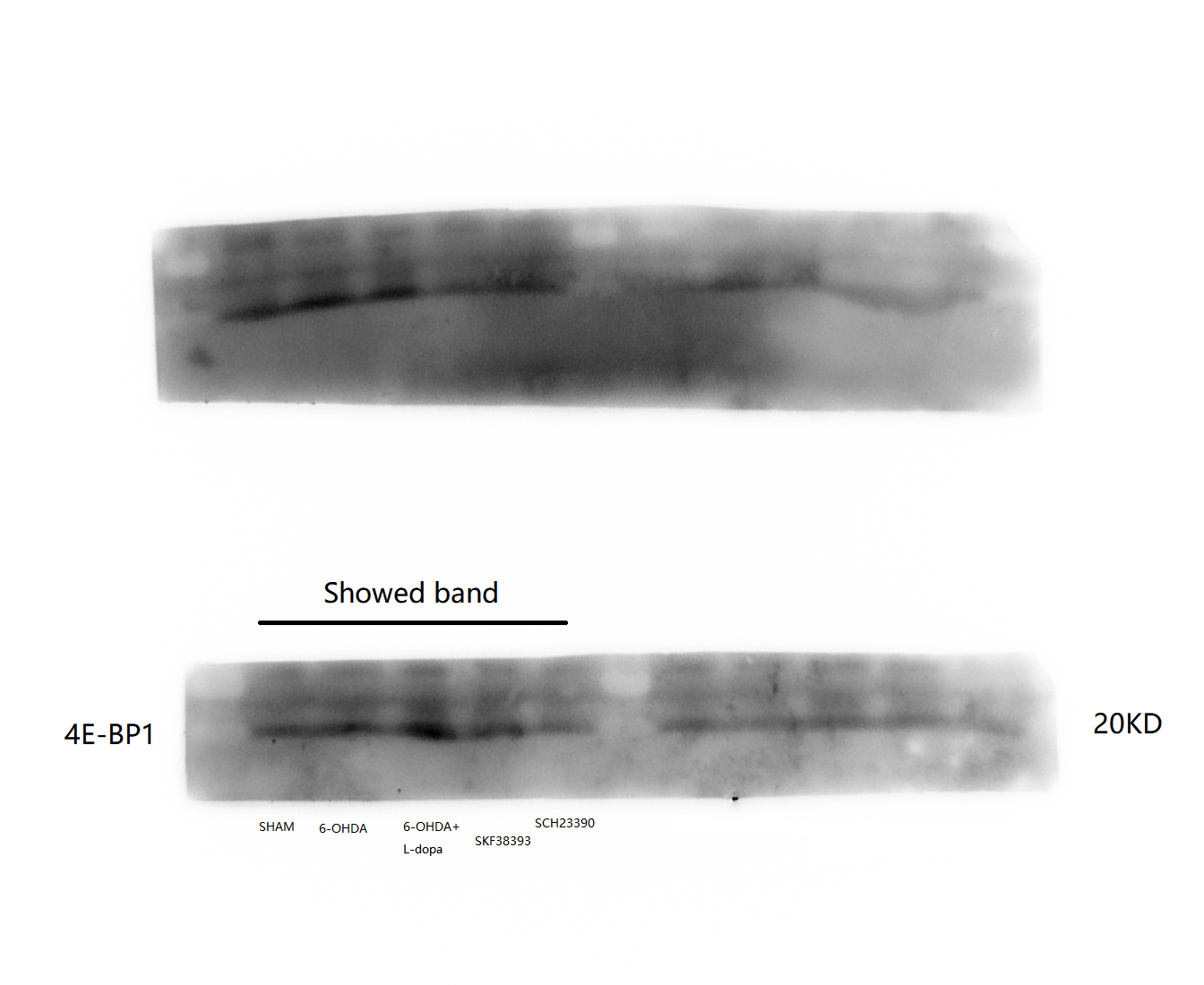

Supplement: Supplementary file 3 [file Data_Sheet_1.ZIP › full scan of the entire original gels/Figure6/4E-BP1/p-4EBP1.tif]

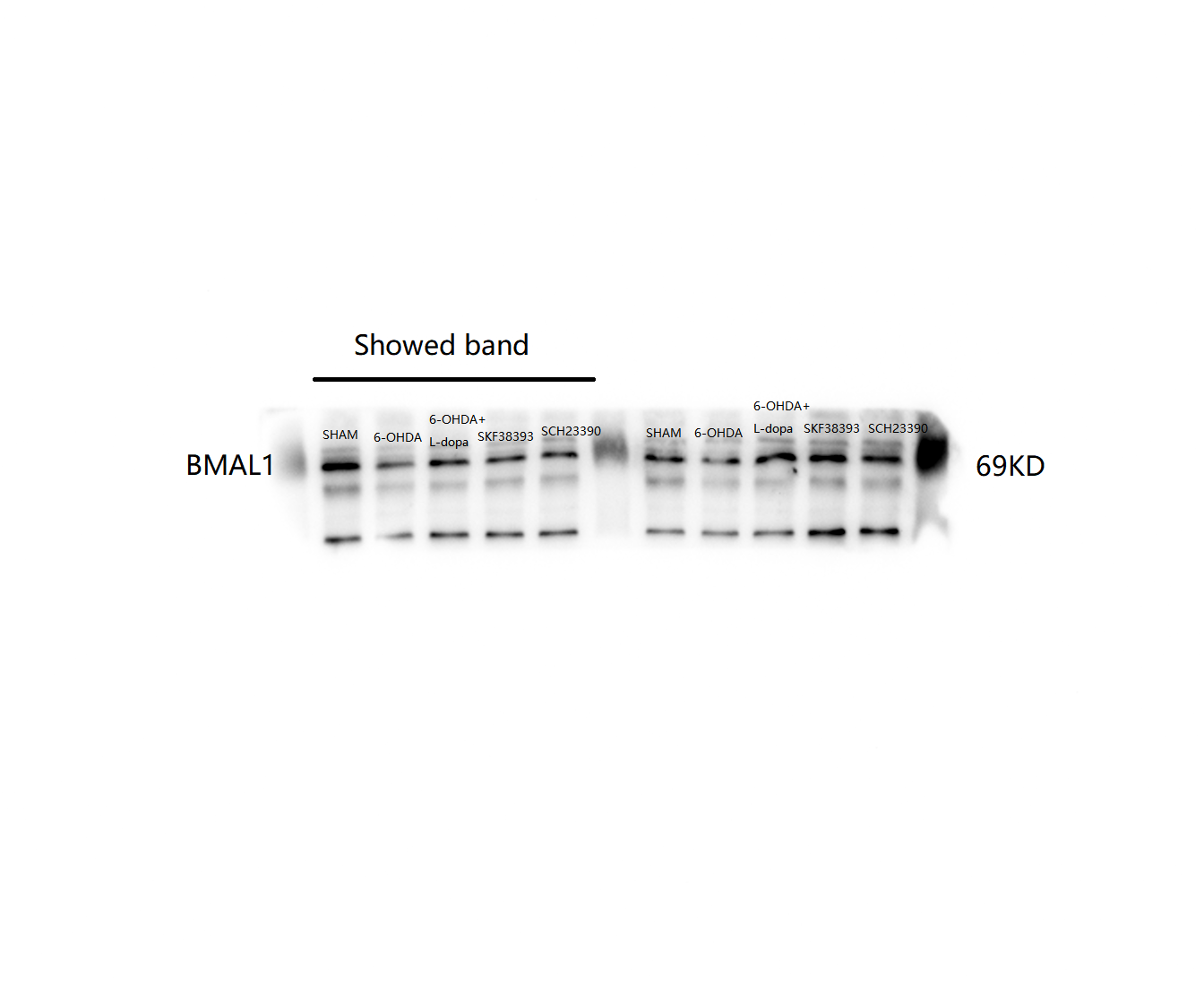

Supplement: Supplementary file 3 [file Data_Sheet_1.ZIP › full scan of the entire original gels/Figure6/BMAL1.Tif]

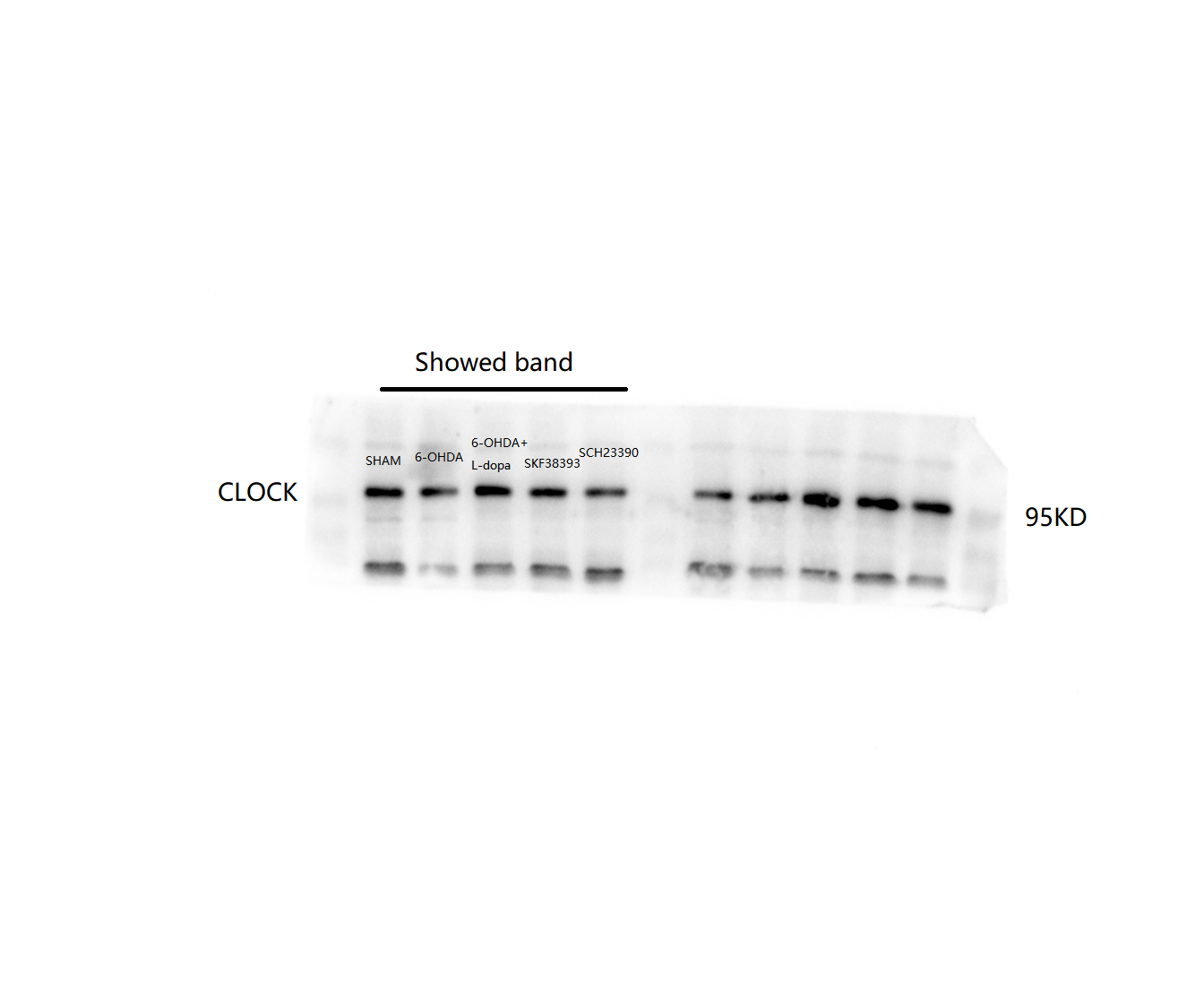

Supplement: Supplementary file 3 [file Data_Sheet_1.ZIP › full scan of the entire original gels/Figure6/CLOCK.Tif]

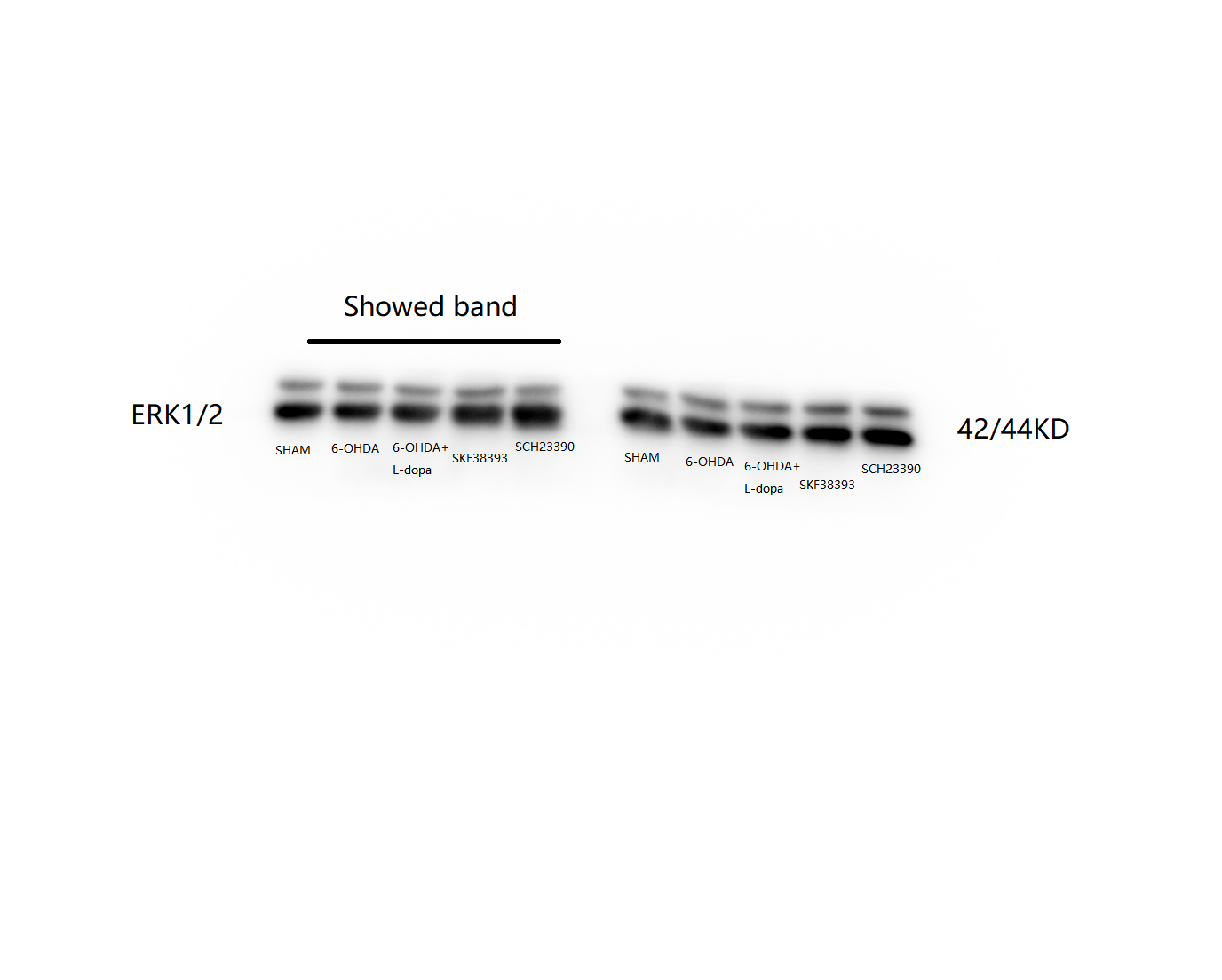

Supplement: Supplementary file 3 [file Data_Sheet_1.ZIP › full scan of the entire original gels/Figure6/ERK/ERK.tif]

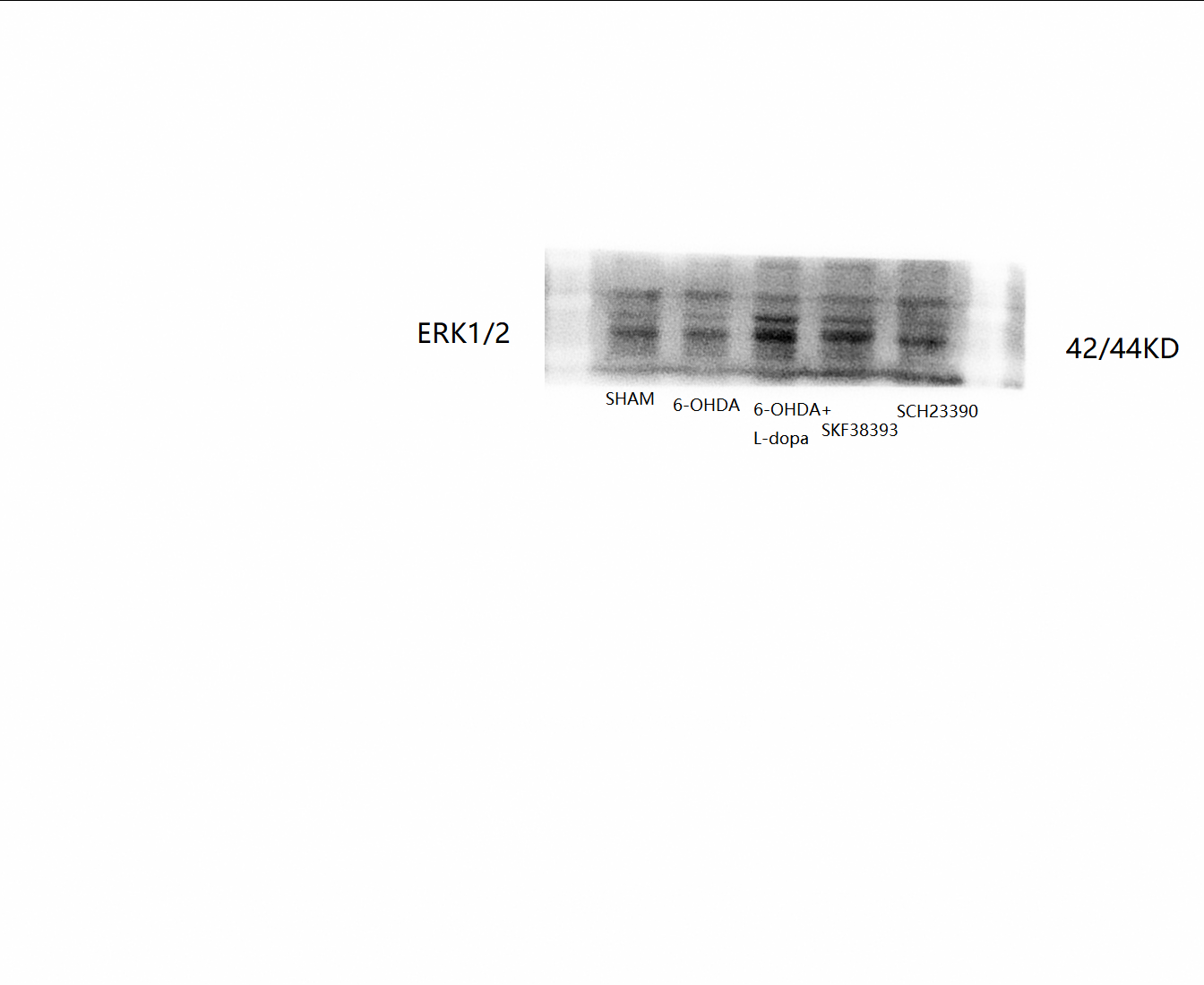

Supplement: Supplementary file 3 [file Data_Sheet_1.ZIP › full scan of the entire original gels/Figure6/ERK/P-ERK.tif]

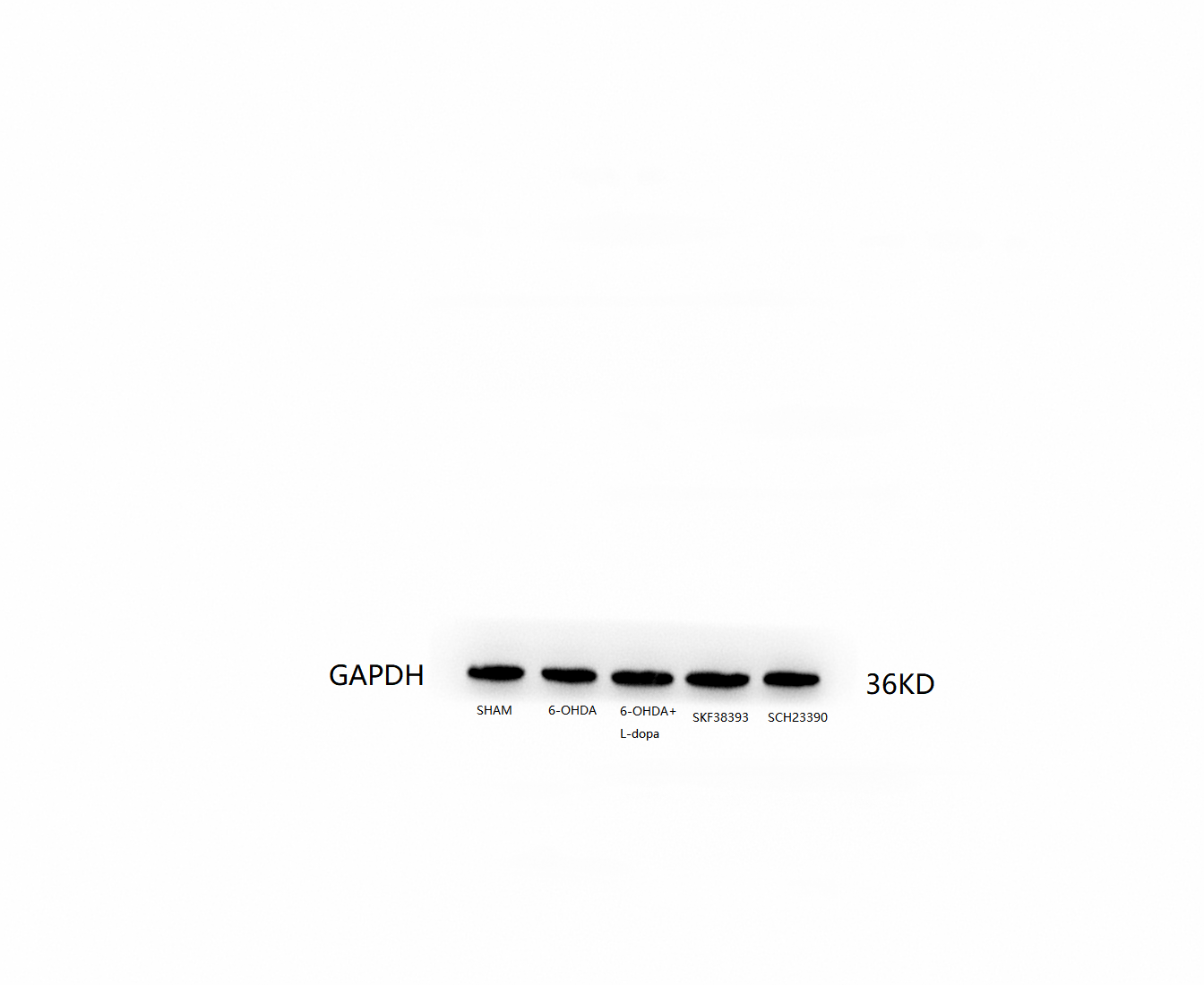

Supplement: Supplementary file 3 [file Data_Sheet_1.ZIP › full scan of the entire original gels/Figure6/GAP.tif]

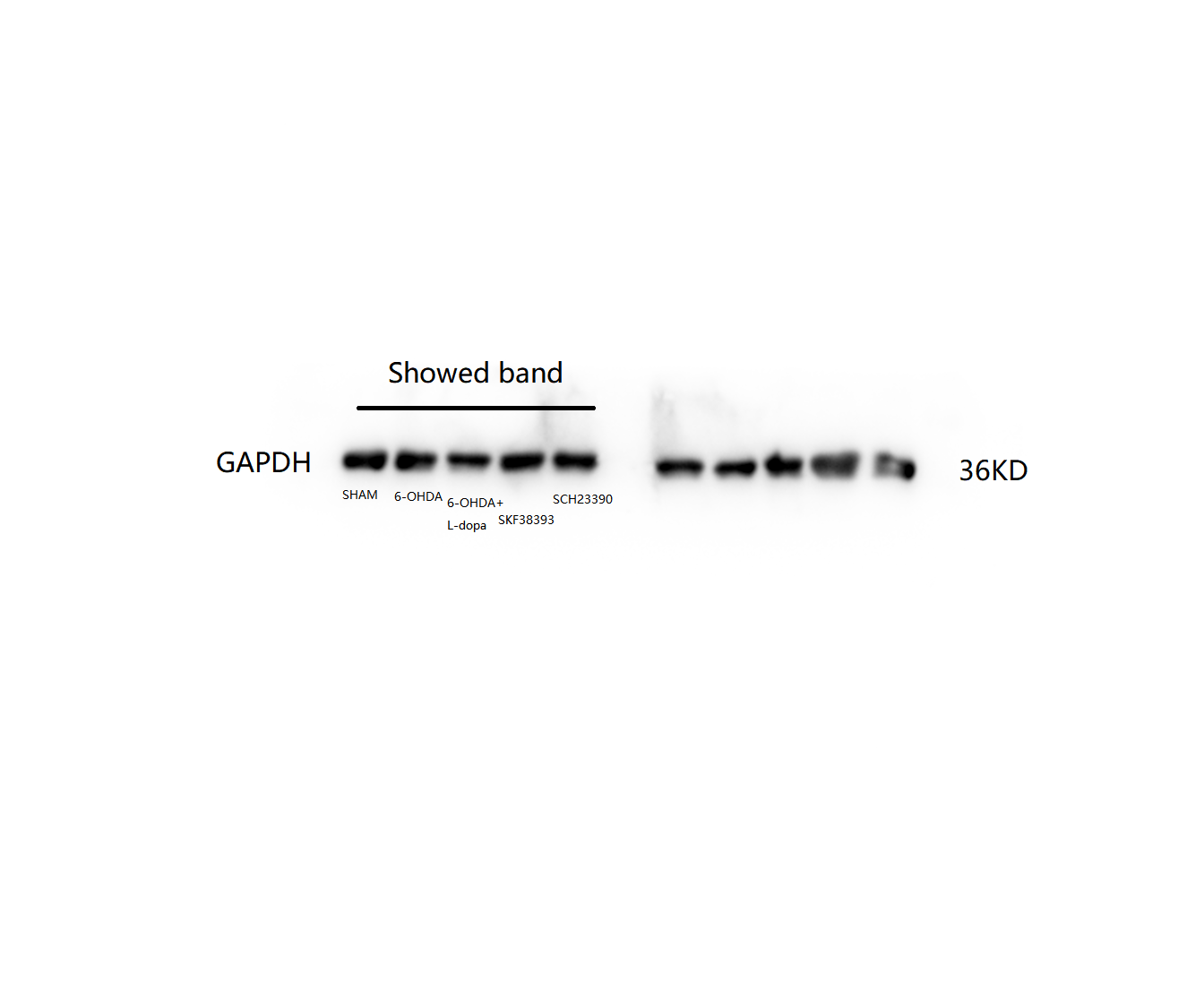

Supplement: Supplementary file 3 [file Data_Sheet_1.ZIP › full scan of the entire original gels/Figure6/mTOR/GAPDH.Tif]

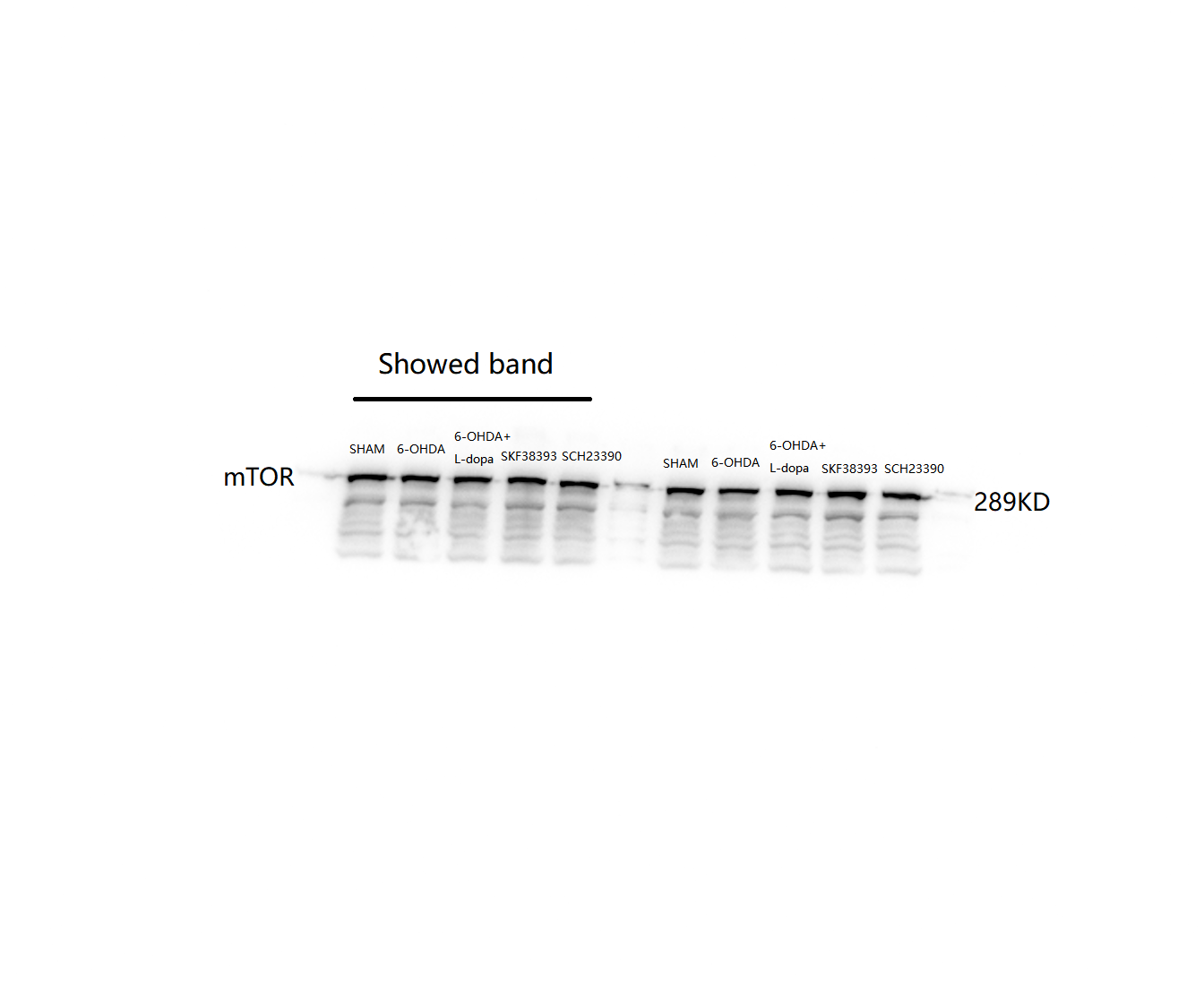

Supplement: Supplementary file 3 [file Data_Sheet_1.ZIP › full scan of the entire original gels/Figure6/mTOR/mTOR.Tif]

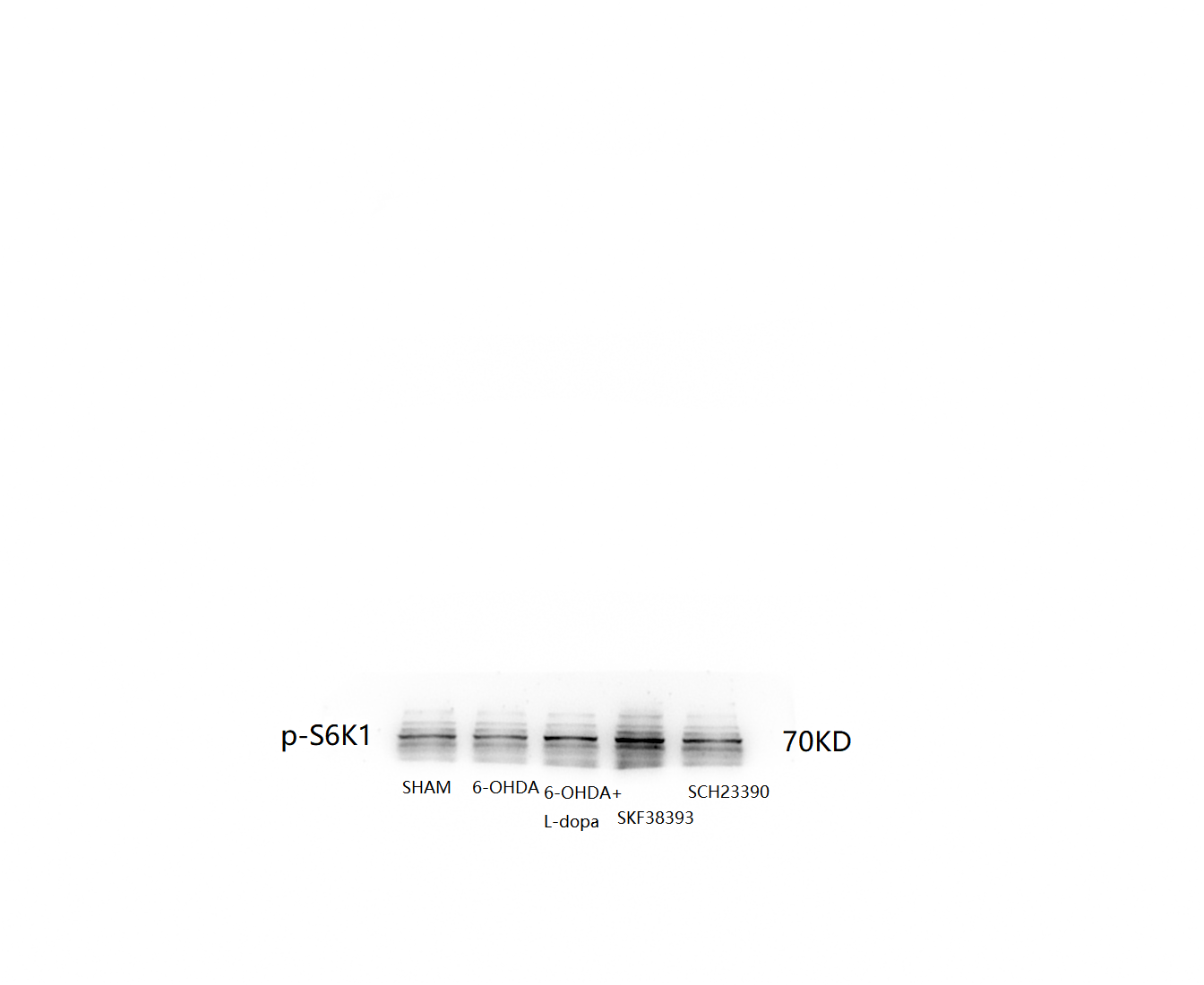

Supplement: Supplementary file 3 [file Data_Sheet_1.ZIP › full scan of the entire original gels/Figure6/S6K1/p-S6K1.tif]

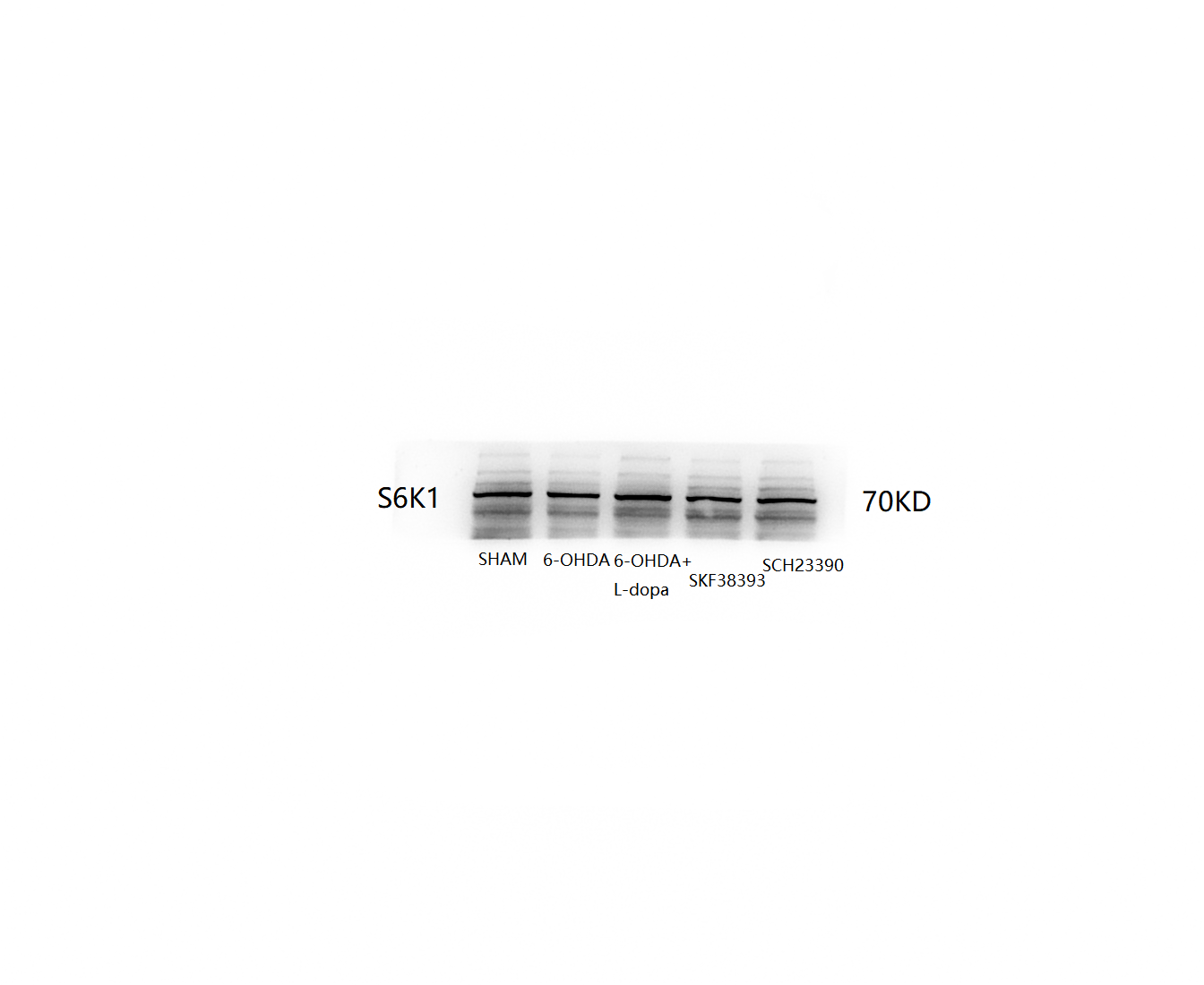

Supplement: Supplementary file 3 [file Data_Sheet_1.ZIP › full scan of the entire original gels/Figure6/S6K1/S6K1.tif]
